# Targeting *Plasmodium falciparum* IspD in the Methyl-d-erythritol Phosphate Pathway: Urea-Based Compounds with Nanomolar Potency on Target and Low-Micromolar Whole-Cell Activity

**DOI:** 10.1021/acs.jmedchem.4c00212

**Published:** 2024-09-20

**Authors:** Daan Willocx, Lorenzo Bizzarri, Alaa Alhayek, Deepika Kannan, Patricia Bravo, Boris Illarionov, Katharina Rox, Jonas Lohse, Markus Fischer, Andreas M. Kany, Hannes Hahne, Matthias Rottmann, Matthias Witschel, Audrey Odom John, Mostafa M. Hamed, Eleonora Diamanti, Anna K. H. Hirsch

**Affiliations:** † Helmholtz Institute for Pharmaceutical Research (HIPS)-Helmholtz Centre for Infection Research (HZI), Campus E8.1, 66123 Saarbrücken, Germany; ‡ Department of Pharmacy, Saarland University, Campus E8.1, 66123 Saarbrücken, Germany; § OmicScouts GmbH, Lise-Meitner-Straße 30, 85354 Freising, Germany; ∥ Department of Pediatrics, Children’s Hospital of Philadelphia, Philadelphia, Pennsylvania 19104, United States; ⊥ Swiss Tropical and Public Health Institute, Kreuzstrasse 2, 4123 Allschwil, Switzerland; # Department of Chemical Biology, Helmholtz Centre for Infection Research (HZI), Inhoffenstraße 7, 38124 Braunschweig, Germany; ∇ German Center for Infection Research (DZIF), Partner Site Hannover-Braunschweig, Inhoffenstraße 7, 38124 Braunschweig, Germany; ○ Universität Basel, Petersplatz 1, 4003 Basel, Switzerland; ◆ Hamburg School of Food Science, University of Hamburg, Grindelallee 117, 20146 Hamburg, Germany; ¶ BASF-SE, Carl-Bosch-Strasse 38, 67056 Ludwigshafen, Germany; †† Perelman School of Medicine, University of Pennsylvania, Philadelphia, Pennsylvania 19104, United States

## Abstract

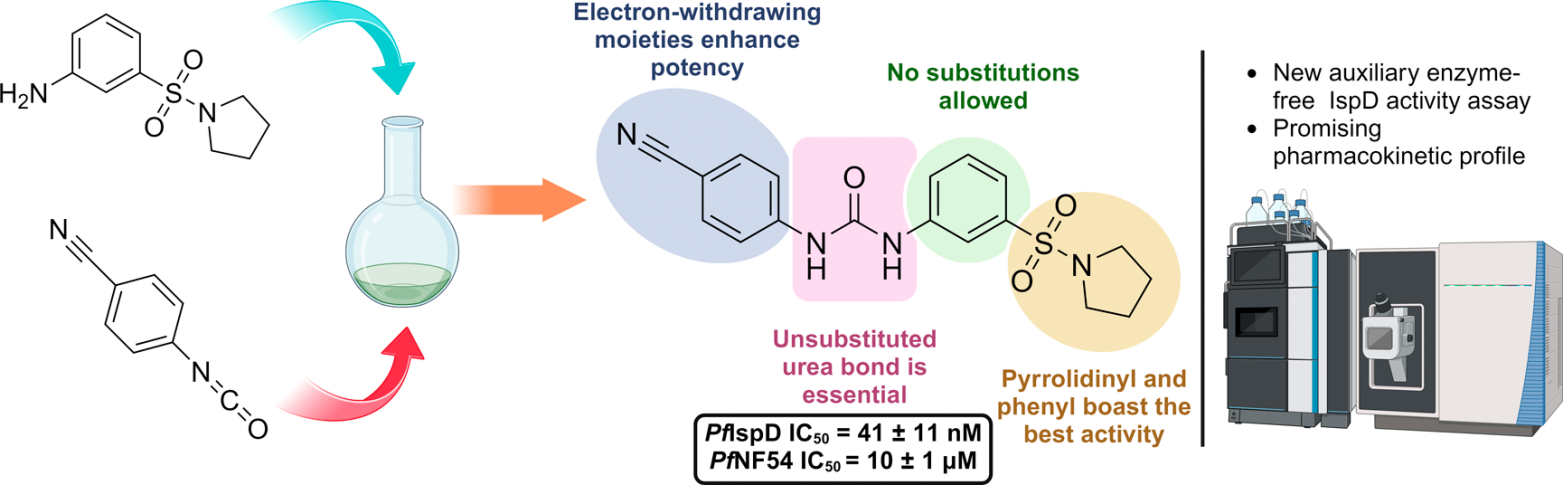

The methyl-d-erythritol phosphate (MEP) pathway has emerged as an interesting target in the fight against antimicrobial resistance. The pathway is essential in many human pathogens, including *Plasmodium falciparum* (*Pf*), but is absent in human cells. In the present study, we report on the discovery of a new chemical class targeting IspD, the third enzyme in the pathway. Exploration of the structure–activity relationship yielded inhibitors with potency in the low-nanomolar range. Moreover, we investigated the whole-cell activity, mode of inhibition, metabolic, and plasma stability of this compound class, and conducted *in vivo* pharmacokinetic profiling on selected compounds. Lastly, we disclosed a new mass spectrometry (MS)-based enzymatic assay for direct IspD activity determination, circumventing the need for auxiliary enzymes. In summary, we have identified a readily synthesizable compound class, demonstrating excellent activity and a promising profile, positioning it as a valuable tool compound for advancing research on IspD.

## Introduction

**Figure 1:**
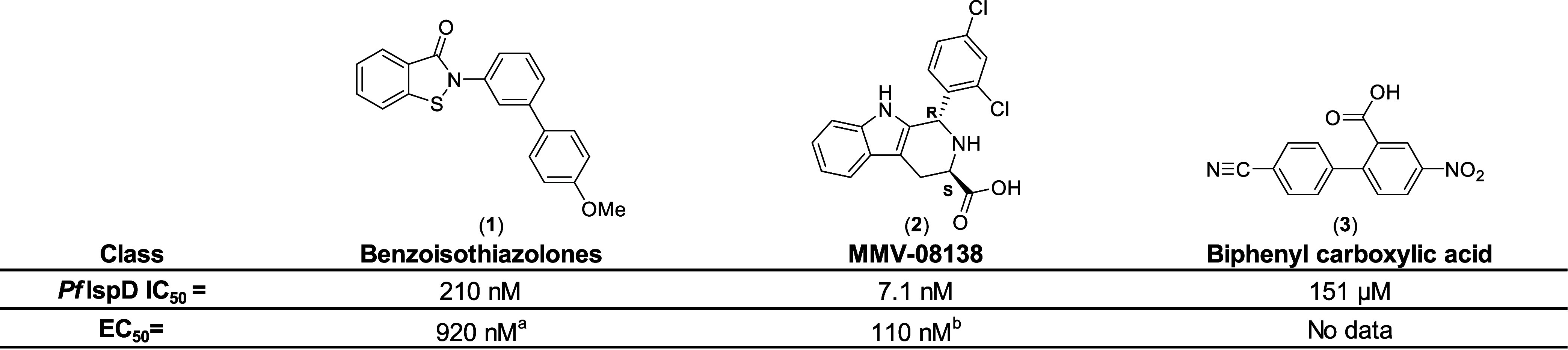
Currently known classes of inhibitors showing enzymatic activity against *Pf*IspD. EC_50_ values were measured against different strains of *Plasmodium falciparum*. ^a^: strain = 3D7; ^b^: strain = W2; Benzoisothiazolones^10^ (**1**), MMV-08138^11−14^ (**2**), Biphenylcarboxylic acid^5^ (**3**).

**Figure 2:**
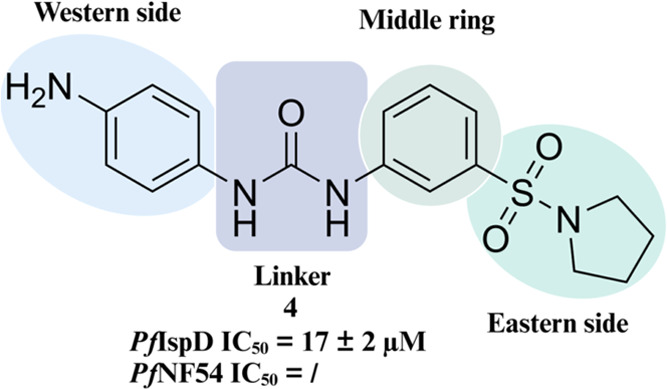
Initial hit compound **4** with an overview of the performed SAR study.

**Scheme 1:**
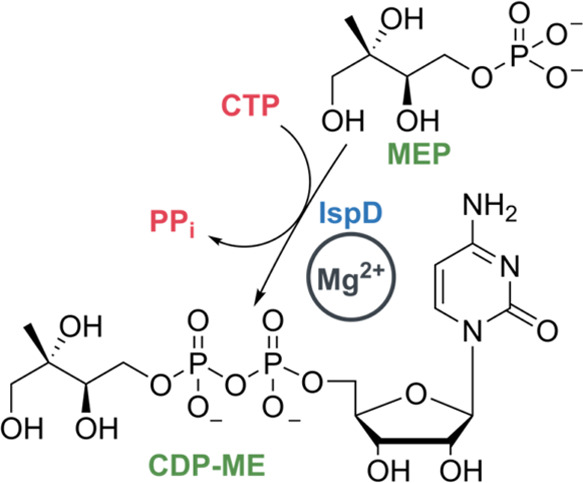
Reaction Catalyzed by IspD Starting from MEP and CTP Affording CDP-ME Mg^2+^ is the cofactor in the reaction.

Since the commercialization of penicillin in the 1940s, Sir Alexander Fleming warned the public about the dangers of antimicrobial resistance (AMR), resulting from overand misuse of anti-infectives. Now, decades later, his warnings are more relevant than ever with AMR reaching alarming levels.^1,2^ A recent example of newly developed resistance is the discovery of artemisinin-resistant strains of *Plasmodium falciparum* (*Pf*), one of the parasites that causes malaria, in Africa, Southeast Asia, the Pacific islands, and Latin America. This discovery is significant as artemisinin-based treatments have been the ‘gold standard’ for malaria treatments for many years, and resistance will have disastrous effects for malaria-prone regions.^3^ The 2-*C*-methylerythritol-d-erythritol-4-phosphate (MEP) pathway, needed for the biosynthesis of the isoprenoid precursors isopentenyl diphosphate (IDP) and dimethylallyl diphosphate (DMADP), is an essential biosynthetic pathway in most Gram-negative bacteria, *Mycobacterium tuberculosis*, and the *Plasmodium parasites*. Furthermore, the MEP pathway is absent in human cells, mitigating the risk of off-target side effects, hence making it a source of promising drug targets.^4,5^ Validation of the MEP pathway enzymes as drug target is based on fosmidomycin, an inhibitor of the second protein, IspC or DXR, of the MEP pathway. Several clinical trials have investigated fosmidomycin in combination therapy for malaria; however, none have achieved cure rates meeting the standards set by the World Health Organization. Nonetheless, a meta-analysis conducted by Fernandes *et al.* revealed a promising 85% cure rate in children, indicating significant potential for fosmidomycin in combination therapy and serving as incentive for further research in this field.^6−8^ Within the present study, we focused on targeting the third enzyme in the MEP pathway, known as, IspD, MEP cytidyltransferase, or *ygbp*. IspD catalyzes the formation of 4-diphosphocytidyl-2-*C*-methylerythritol (CDP-ME) from MEP and cytidine triphosphate (CTP) in the presence of Mg^2+^, releasing inorganic diphosphate (PP_i_) (Scheme 1).^9^ Previously reported inhibitors targeting *Pf*IspD can be subdivided into three chemical classes, namely, benzoisothiazolones **1**, identified by a combined approach of cheminformatics and high-throughput enzymatic screening, MMV008138 **2** recognized through phenotypic screening of the library of GlaxoSmithKline and last, a biphenyl carboxylic acid fragment **3** recently discovered by our group in collaboration with BASF (Figure 1). Despite the potential of IspD as a drug target, the number of IspD inhibitors reported is rather low. Furthermore, the reported inhibitors are rather challenging to synthesize or lack whole-cell activity.^5,10−14^ A possible cause might be the lack of a crystal structure of *Pf*IspD available in the Protein Data Bank (PDB). Here, we report the structure–activity relationship (SAR) study of a new urea-based compound class targeting *Pf*IspD with low-nanomolar activity *in vitro*. Its synthesis is straightforward, in one step from the corresponding aniline and isocyanate. A high-throughput screening (HTS) approach on *P. vivax* IspD and subsequent confirmation of hits concomitantly on *Pf*IspD and *Pf*NF54 led to the discovery of the initial hit (**4**, Figure 2) endowed with an IC_50_ of 17 ± 2 μM against *Pf*IspD but lacking whole-cell activity. Synthesis of a total of 34 derivatives shed a light on the SAR of this newly discovered class reaching IC_50_ values as low as 41 nM with a whole-cell activity in the low micromolar range. Throughout our study, we made efforts to maintain the easily synthesizable core of the urea class, ensuring the molecule’s accessibility, of particular improtance for antimalarials that are predominantly utilized in low- and middle-income countries.

## Results and Discussion

### SAR Exploration

**Scheme 2:**
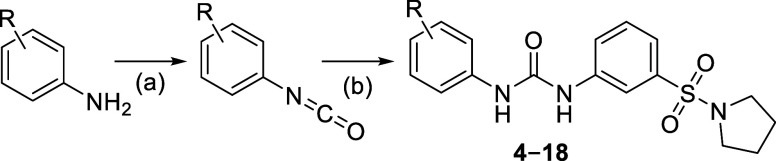
General Synthetic Procedure Followed for the Synthesis of **4–18** Reagents and conditions: (a) triphosgene, Et_3_N, DCM, 0 °C to room temperature, 3 h, used without purification in the next reaction step; (b) 3-(pyrrolidin-1-ylsulfonyl)aniline, DMF, room temperature, overnight, 8–95% yield.

**Scheme 3:**
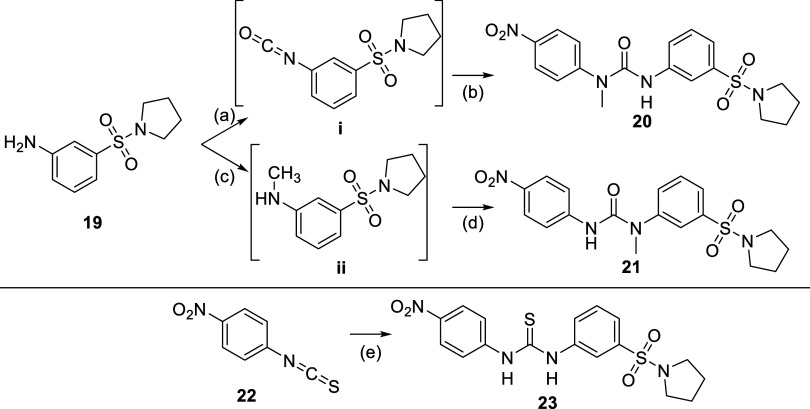
Synthetic Procedure Followed for the Synthesis of **20**, **21**, and **23** Reagents and reactions conditions: (a) triphosgene, Et_3_N, DCM, 0 °C to room temperature, 3 h; (b) *N*-methyl-4-nitroaniline, NaH, DMF, room temperature, 1 h. 32% yield over two steps; (c) *para*formaldehyde, NaBH_4_, MeOH, at room temperature for 2.5 h to 60 °C for 16 h, 57% yield; (d) 1-isocyanato-4-nitrobenzene, DMF, room temperature for 16 h, 12% yield. (e) 3-(pyrrolidin-1-ylsulfonyl)aniline, DMF, room temperature, 48 h, 35% yield.

**Scheme 4:**
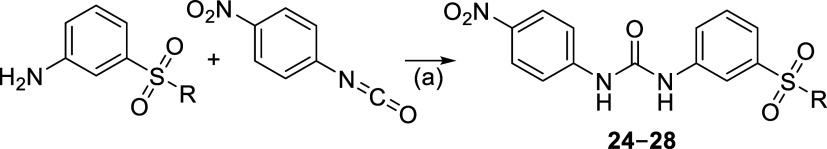
General Synthetic Procedure Followed for the Synthesis of **24–28** Reagents and conditions: (a) 1-isocyanato-4-nitrobenzene, DMF, room temperature, overnight, 28–58% yield.

**Scheme 5:**
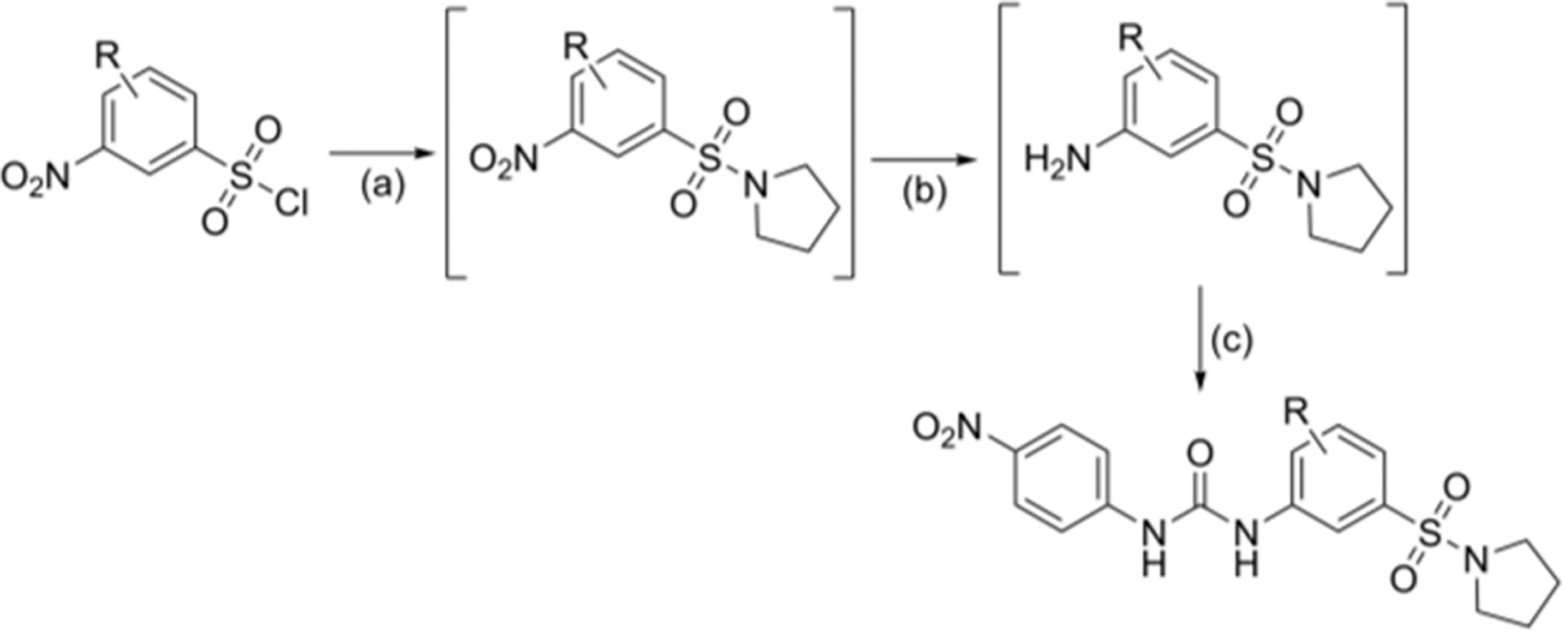
Synthesis of Compounds **34–40** Reagents and reactions conditions: (a) respective 3-nitrobenzenesulfonyl chloride, pyrrolidine, triethylamine, acetonitrile, 0 °C, 5 min; (b) Fe powder, NH_4_Cl (166 mM in water), EtOH, 80 °C, 2.5 h; (c) 1-isocyanato-4-nitrobenzene, DMF, room temperature, 4 h, 5–37% yield over three steps.

We commenced exploration of the initial hit by synthesizing derivatives with diverse moieties on the Western phenyl ring (Figure 2). Compounds **4**–**18** were synthesized as depicted in Scheme 2. Depending on commercial availability, we either generated isocyanates *in situ* by reacting the respective amine with triphosgene or purchased them. Nucleophilic addition between 3-(pyrrolidin-1-ylsulfonyl)aniline and the respective isocyanate afforded the desired compounds. At first, we directed modifications toward the primary amine and replaced it by moieties with different electronic effects (**5**–**11**) (Table 1). We observed that more electron-withdrawing substituents, such as a nitro (**8**) or nitrile group (**10**), had a pronounced effect on the potency, resulting in a 400-fold increase (*e.g.*, **8**, *Pf*IspD IC_50_ = 41 ± 7 nM). While weaker electron-withdrawing substituents , such as the trifluoromethyl (**11**) and the chloride (**5**), had a lower effect on the potency (*e.g.*, **11**, *Pf*IspD IC_50_ = 91 ± 19 nM). Lastly, weakly electron-donating groups, such as methyl (**6**), lead to an even smaller increase in potency (*e.g*., **6**, *Pf*IspD IC_50_ = 370 ± 80 nM), but were still significantly better than the initial hit compound **4**. The smaller increase in potency of **9** (*Pf*IspD IC_50_ = 330 ± 40 nM) could possibly be attributed to the size of the substituent as will be seen later. In addition, low-micromolar activity in the whole-cell assay was noted for these derivatives. Next, we explored various substitution patterns on the phenyl ring **12**–**14**. Placement of trifluoromethyl in *meta* position (**12**, *Pf*IspD IC_50_ = 415 ± 60 nM) did not improve upon its *para*-substituted counterpart (**11**, *Pf*IspD IC_50_ = 91 ± 19 nM). Furthermore, having multiple substituents (**13**–**14**) did also not lead to improvements in *Pf*IspD activity over the monosubstituted derivatives. To determine whether there was still room for growth on the Western side, we synthesized analogues **15**–**18** using the general synthetic route depicted in Scheme 2. Growth in this direction resulted in a significant loss in activity, which we interpret as a lack of space for further expansions. For the remainder of the SAR study, we selected **8** as scaffold for derivatization. Next, we focused on the urea linker itself (Table 2). Both positions of the urea bond were methylated successively as depicted in Scheme 3. To ensure selective methylation, **20** was synthesized by first transforming 3-(pyrrolidin-1-ylsulfonyl)aniline (**19**) into the corresponding isocyanate with triphosgene, followed by addition of deprotonated *N*-methyl-4-nitroaniline to the reaction mixture. On the other hand, **20** was synthesized *via* two steps. First, a reductive amination between 3-(pyrrolidin-1-ylsulfonyl)aniline and *para*formaldehyde resulted in *N*-methyl-3-(pyrrolidin-1-ylsulfonyl)aniline to which 1-isocyanato-4-nitrobenzene was added, resulting in a nucleophilic addition affording **21**. Unfortunately, methylation of either site of the urea bond led to complete loss of activity. A possible explanation for this observation could be the loss of hydrogen-bonding interactions or conformational changes imposed by the methylation. Next we explored the possibility of replacing the urea with a thiourea (**23**) by employing an isothiocyanate **22** in the synthesis instead of an isocyanate (Scheme 3, bottom). This modification resulted as well in a decrease in activity (**23**, *Pf*IspD IC_50_ = 395 ± 60 nM) in comparison with its urea counterpart (**8**, *Pf*IspD IC_50_ = 41 ± 7 nM). Subsequent modifications explored the Eastern side of the molecule (Figure 2). For the synthesis of these derivatives (**24**–**28**), we used the synthetic procedure as depicted in Scheme 4. Initially, we replaced the pyrrolidine with more flexible dimethyl (**24**) and diethyl (**25**) amine groups (Table 2). These derivatives did not manage to enhance activity (**24**, *Pf*IspD IC_50_ = 180 ± 20 nM) over the pyrrolidinyl-containing parent compound (**8**, *Pf*IspD IC_50_ = 41 ± 7 nM) (Table 2). Ring expansion toward piperidine (**26**) and morpholine (**27**) likewise failed to increase activity. On the other hand, when we substituted the pyrrolidinyl with a phenyl ring, the activity could be retained (**28**). In the next phase, we assessed the role of the sulfonyl linker in the compounds’ activity. To this end, we synthesized compounds **29** and **30**. For the synthesis of compound **29**, we began with amide-bond formation between 3-nitrobenzoic acid and pyrrolidine using propanephosphonic acid anhydride as the coupling reagent (Scheme S1). Subsequently, we reduced the nitro group to the corresponding amine, which was then reacted with 1-isocyanato-4-nitrobenzene, yielding the desired compound. For the synthesis of compound **30**, we employed a similar reaction scheme starting from 3-nitrobenzyl bromide (Scheme S2). A nucleophilic substitution reaction with pyrrolidine produced, an intermediate, of which the nitro group was then reduced to the corresponding amine. This amine was reacted with 1-isocyanato-4-nitrobenzene to afford the final compound. As shown in Table 3, replacing the sulfonyl linker resulted in a significant decrease in activity toward *Pf*IspD. Therefore, we reason that the sulfonyl linker is crucial for the activity of this compound class. Lastly, we explored a handful of compounds containing modifications on the middle ring (Table 4). Initially, these compounds were synthesized containing a morpholine (**31**–**33**) instead of a pyrrolidine, as anilines of these derivatives were commercially available. Interestingly, compound **31** (*Pf*IspD IC_50_ = 395 ± 3.5 nM) showed enhanced activity over parent compound **27** (*Pf*IspD IC_50_ = 600 ± 110 nM). Consequently, we decided to construct derivatives containing the pyrrolidine (**34**–**40**). A three-step synthesis led to compounds **34**–**40** (Scheme 5). As a first step, a nucleophilic substitution reaction took place between pyrrolidine and the respective 3-nitrobenzenesulfonyl chloride. Next, the nitro group was reduced to the amine, which was then reacted with 1-isocyanato-4-nitrobenzene, affording the desired urea compounds (Table 4). After examination of these compounds in our *in vitro* assays, we could not observe any increase in potency over **8**, even not for the derivative containing the 4-fluoro moiety (**34**), which previously triggered a rise in potency. In summary, modifications directed to the Western side of the molecule are most beneficial for the activity of the compound class. Positioning electron-withdrawing substituents at the *para* position induced the most notable changes, enabling compounds **8** and **10** to reach IC_50_ values of 41 nM. Other substituents or further expansions at this position did not achieve such an increase in potency. In addition, an unsubstituted urea linker is detrimental for the activity of the compound class. Attempts to modify the middle ring turned out to be futile as any placement of a moiety led to a decrease in activity. On the Eastern side of the molecule, a pyrrolidinyl or phenyl ring led to the highest *in vitro* activity. Furthermore, we observed that the sulfonyl linker is essential for the *in vitro* activity of the compound class. Overall, compounds **8**, **10**, and **28** are seen as frontrunners of the urea class, exhibiting the best *in vitro* activity, while also showing modest activity in the whole-cell assay. Interestingly, compound **28** constitutes a potential starting point to further explore the urea class by placing substituents on the phenyl ring or by growing in this direction.

**Table 1:**
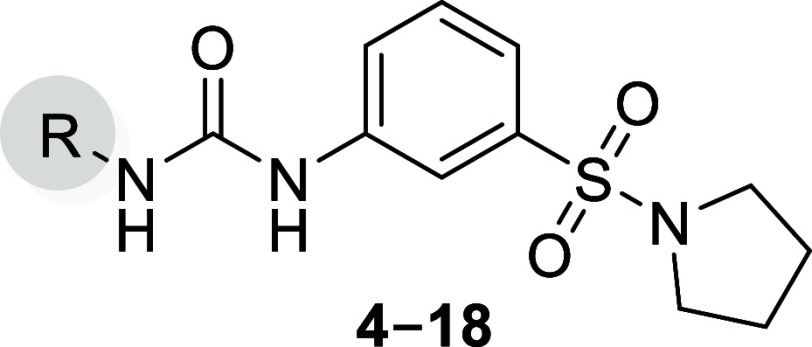

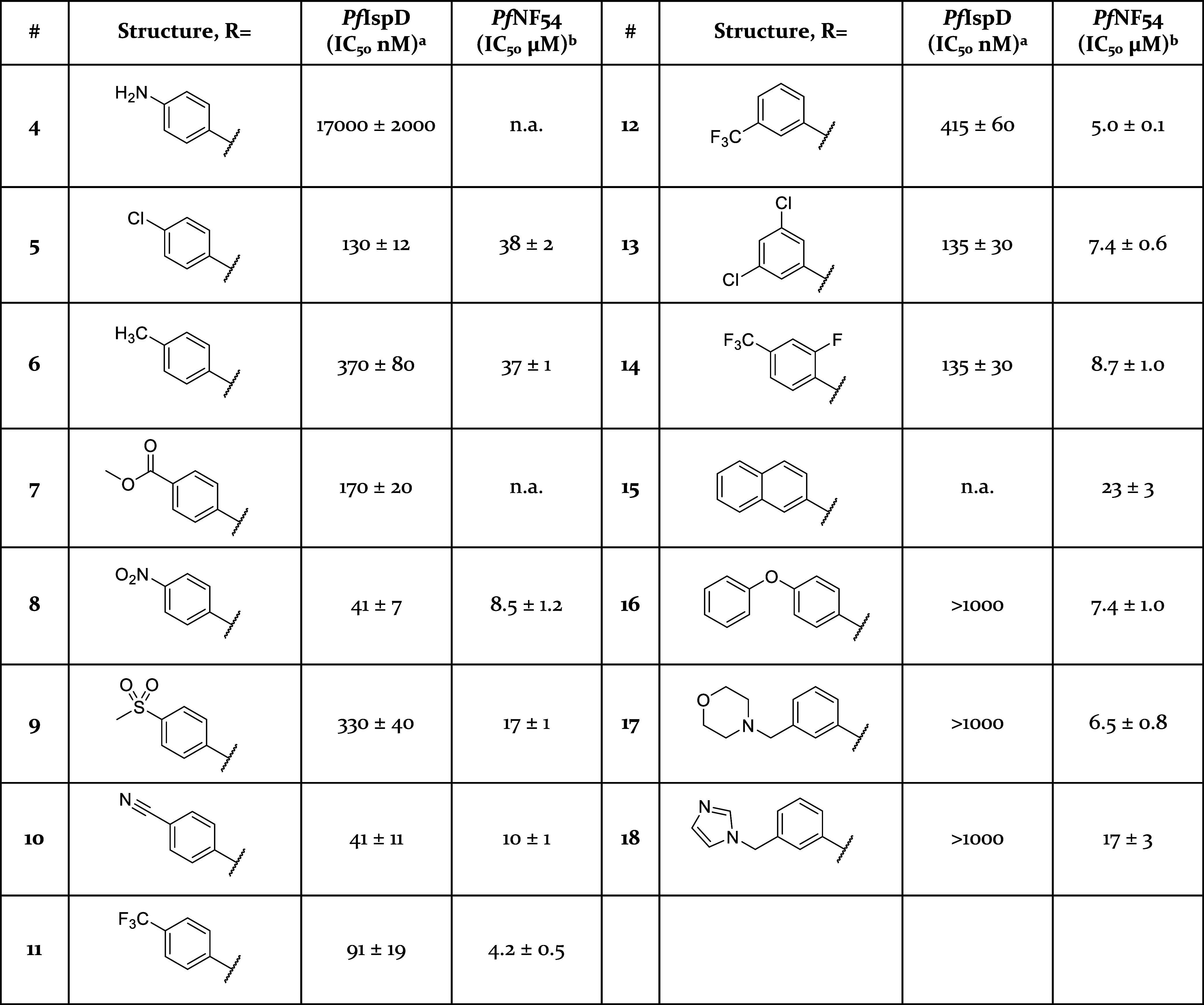
*In Vitro* and Whole-Cell Activities for Compounds **4–18**

a Assays were performed in replicate as independent experiments (*n* ≥ 2); values are shown as mean ± SD.

b Assays were performed in replicate as independent experiments (*n* = 2); values are shown as mean ± SD n.a. indicates the absence of activity.

**Table 2:**
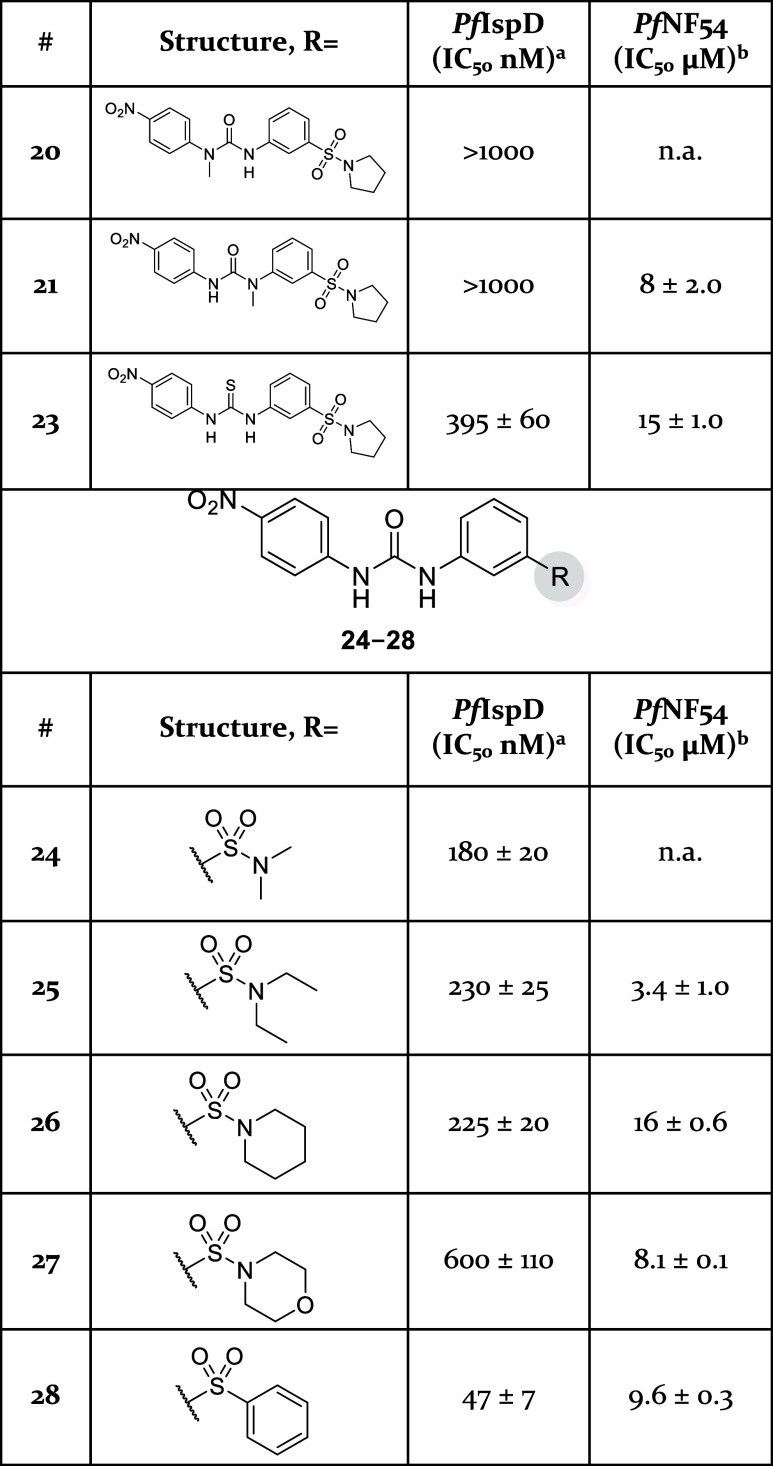
*In Vitro* and Whole-Cell Activities for Compounds **20**, **21**, and **23–28**

a Assays were performed in replicate as independent experiments (*n* ≥ 2); values are shown as mean ± SD.

b Assays were performed in replicate as independent experiments (*n* = 2); values are shown as mean ± SD n.a. indicates the absence of activity.

**Table 3:**
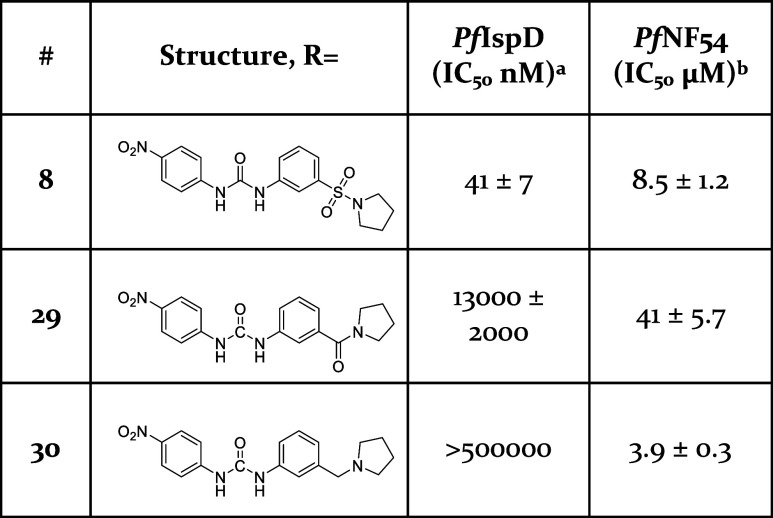
*In Vitro* and Whole-Cell Activities for Compounds **8**, **29**, and **30**

a Assays were performed in replicate as independent experiments (*n* ≥ 2); values are shown as mean ± SD.

b Assays were performed in replicate as independent experiments (*n* = 2); values are shown as mean ± SD.

**Table 4:**
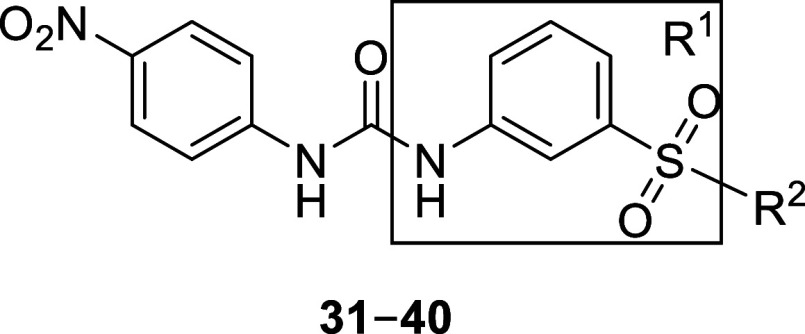

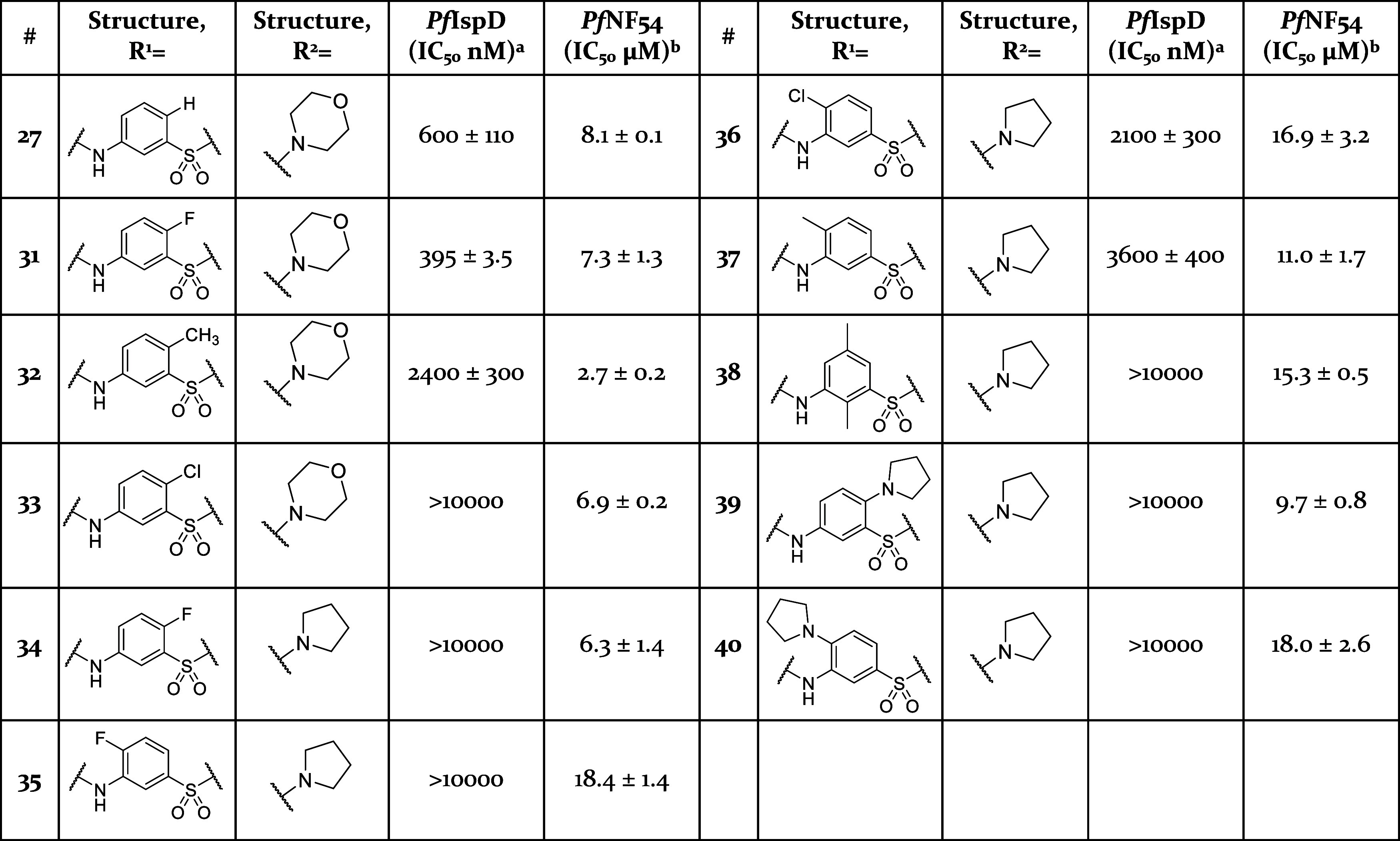
*In Vitro* and Whole-Cell Activities for Compounds **31–40**

a Assays were performed in replicate as independent experiments (*n* ≥ 2); values are shown as mean ± SD.

b Assays were performed in replicate as independent experiments (*n* ≥ 2); values are shown as mean ± SD n.a. indicates the absence of activity.

### Validating IspD as Target of the Urea Class

**Figure 3:**
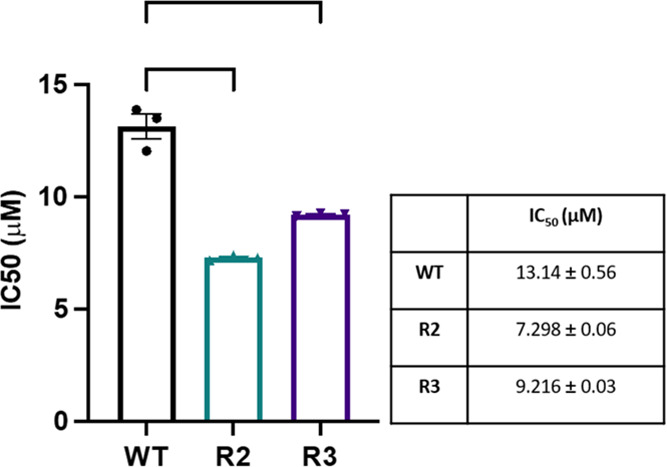
Comparison of the whole-cell activity of **10** toward one wild type strain (WT, MR4) and two MMV008138 resistant strains of *P. falciparum*. Statistical analysis—one-way anova, R2: P* < 0.0001, R3: P*—0.0003

**Figure 4:**
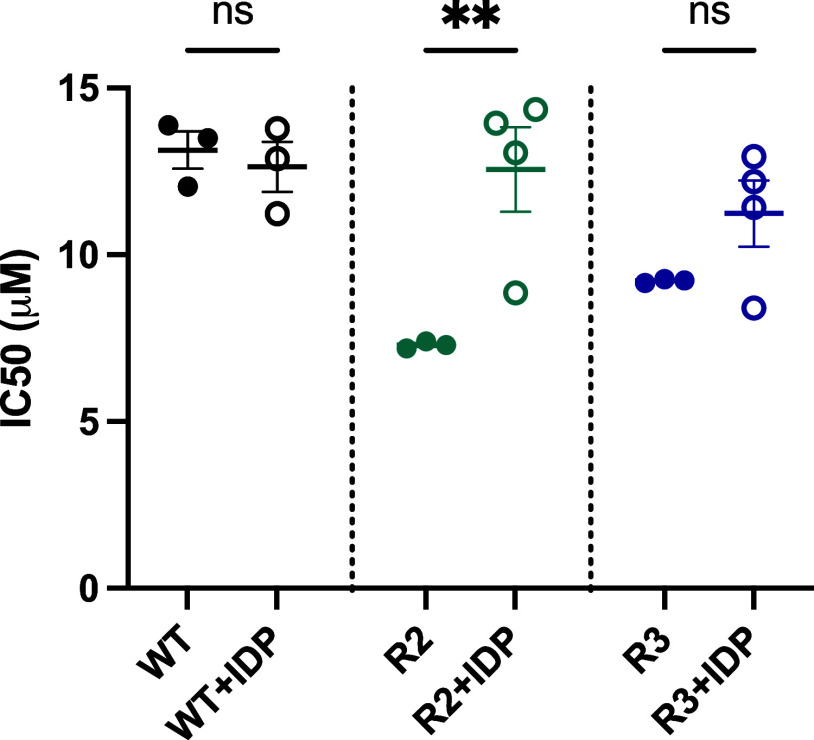
Difference in whole-cell activity of **10** between non supplemented and IDP supplemented conditions. WT = wild type; R2 and R3 are MMV008138 resistant strains.

A discrepancy was observed in the activity trends of compounds from this class when comparing on target and whole-cell assays. For instance, compound **10** (*Pf*IspD IC_50_ = 41 ± 7 nM) significantly outperforms compound **32** (*Pf*IspD IC_50_ = 2400 ± 300 nM) in *Pf*IspD activity. However, in the whole-cell assay, compound **32** (*Pf*NF54 IC_50_ = 2.7 ± 0.2 μM) shows better performance than compound **10** (*Pf*NF54 IC_50_ = 10 ± 1 μM). This inconsistency between *Pf*IspD activity and whole-cell activity could be attributed in some part to off-target effects. To verify if IspD is contributing to the cellular activity, we measured the whole-cell activity of compound **10** against a wild-type (MR4) strain and two MMV008138-resistant strains (R2 and R3) of *P. falciparum*. As shown in Figure 3, both resistant strains appear more susceptible to compound **10** than the wild type. We hypothesize that this increased susceptibility is due to a less fit IspD enzyme in the resistant parasites. Mutations in IspD that confer resistance to MMV008138 may alter the enzyme, making resistant parasites more susceptible to other IspD inhibitors. This suggests that IspD is a target of this class of compounds. To confirm this observation, we repeated the assay, this time supplementing the parasites with 200 μM IDP, the end product of the MEP pathway. The addition of IDP equalized the sensitivity of the resistant strains and the wild type toward compound **10**. This confirmed our hypothesis that IspD is indeed one of the *in vivo* targets of this compound class (Figure 4).

### LC-MS Based Activity Assay

To gain an idea on the mode of inhibition of our new compound class, we intended to do a characterization of the enzyme kinetics under a range of inhibitor concentrations. Our intention was to perform this experiment without the influence of auxiliary enzymes inherent to the photometric assay used for IC_50_ determinations.^15^ To achieve this goal, we sought to uncover a way to measure the progress of the enzymatic reaction without relying on any secondary reactions. In our exploration, we encountered the work of Li *et al.*, who successfully profiled and quantified MEP metabolites in leaves using liquid chromatography-tandem mass spectrometry.^16^ With this information in hand, we sought to develop an IspD activity assay based on the LC-MS detection and quantification of both substrate and product. Initial experiments revealed a significantly more pronounced signal for CDP-ME compared to MEP, with the latter often indistinguishable from background noise. An explanation for this observation might be the difference in ease of ionization, with CDP-ME being more readily ionizable than MEP. Furthermore, we observed identical fragmentation for MEP and the MEP part of CDP-ME, resulting in an overestimation of the MEP concentration. Hence, we decided to continue the assay development relying on the quantification of the product, CDP-ME. Calibration curves measured for CDP-ME demonstrated a linear progression for a wide concentration range showing an *R*^2^ of 0.99 (Figure S1). Finally, an internal standard was chosen, initially several unreactive ATP derivatives, such as adenylyl-imidodiphosphate and adenosine-5′-[(α,β)-methyleno]triphosphate were tested, but those exhibited long elution times of 20 to 30 min. Ultimately, we chose 4-methyl-1-oxo-1-(*p*-tolylamino)pentane-2-sulfonic acid as our internal standard, as its elution time was in the range of that of CDP-ME and showed consistent results.^17^ To demonstrate the potential of our new assay, we determined the Michaelis constant (*K*_m_) of both substrates, and obtained similar results as previously published (Table 5).^12,14,18^ To our knowledge, this is the only reported IspD assay that is not dependent on auxiliary enzymes.

**Table 5:** Comparison of Michaelis Constants

	*K*_m_^CTP^ (μM)	*K*_m_^MEP^ (μM)
our results^a^	58 ± 9	46 ± 3
Wu et al.^14^	not reported	61
Imlay et al.^12^	59 ± 4	not reported
Ghavami et al.^18^	9 ± 3	12 ± 3

a Assays were performed in replicate as independent experiments (*n* = 2); values are shown as mean ± SD.

### Mode of Inhibition

**Figure 5:**
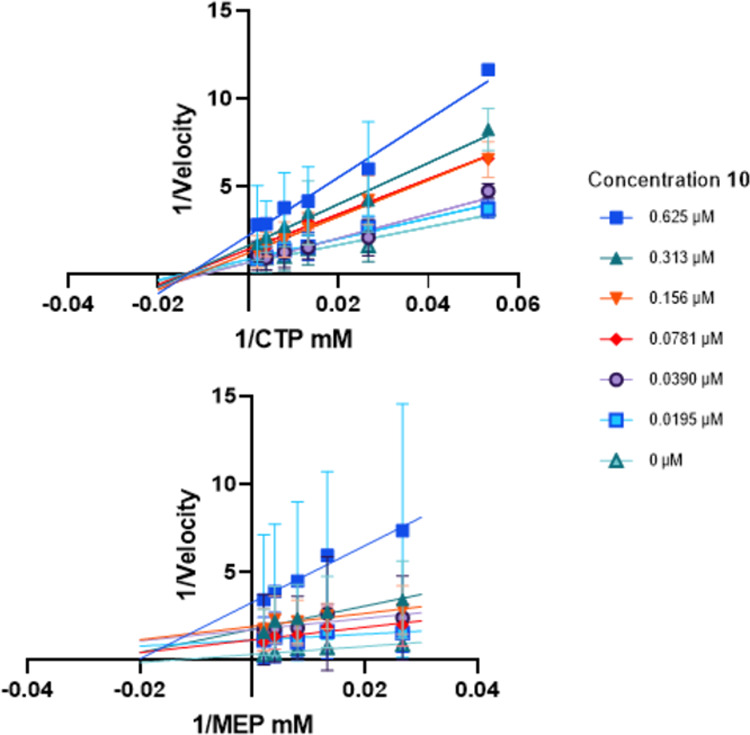
Inhibition of *Pf*IspD by **10** is characterized as noncompetitive with CTP, while uncompetitive with MEP. Lineweaver–Burk plots of both substrates at varying concentrations of **10**. Above: CTP was varied; below: MEP concentration was varied.

Next, we measured the influence of **10** on the enzymatic kinetics of both substrates at different concentrations, ranging from 19.5 to 625 nM. The corresponding Lineweaver–Burk plots hint toward a noncompetitive inhibition of **10** toward CTP and uncompetitive toward MEP (Figures 5, S2, and S3). This finding indicates that compound **10** binds to *Pf*IspD in a manner independent of CTP binding to the active site. In this way, it influences the catalytic activity of the enzyme without affecting CTP binding. This highlights an allosteric inhibition mechanism of the enzyme, which, has been observed before for *Arabidopsis thaliana* IspD by Witschel and co-workers but has never been observed previously for *Pf*IspD.^19^ On the other hand, compound **10** selectively targets the *Pf*IspD-MEP complex, influencing the catalytic activity of the enzyme as well as substrate binding. These findings unravel the selectivity of compound **10** against both substrates, revealing distinct modulatory effects dependent on the substrate specificity of *Pf*IspD.

### Metabolic and Plasma Stability

To gain an initial understanding of selected *in vitro* ADMET properties of the urea class, the metabolic and plasma stability of selected compounds (**5**, **8**, **10**, and **28**), was determined in liver S9 fractions and plasma of both mouse and human (Table 6). Clearance in mouse S9 liver fraction was high to moderate, with compounds **5**, **6**, and **28** showing lower clearance than **8**. In human S9, clearance showed a similar trend with generally lower turnover. No metabolism during 120 min was observed in human S9 fractions for **28**. Regarding plasma stability, all selected compounds showed complete stability in both species (Table 6). Lastly, we also assessed the cytotoxicity toward HepG2 cells. No significant cytotoxicity was observed for **28** up to 100 μM, while the other compounds showed an CC_50_ range of 29–62 μM.

**Table 6:** *In Vitro* Metabolic and Plasma Stability of Compounds **5**, **8**, **10**, and **28**

model system		5	8	10	28
mouse liver S9	*T*_1/2_ [min]^a^	23 ± 3	11 ± 3	20 ± 5	23 ± 3
	Cl_int_ [μL/min/mg]^a^	31 ± 4	66 ± 20	36 ± 10	31 ± 4
human liver S9	*T*_1/2_ [min]^a^	91 ± 19	53 ± 11	69 ± 12	>120
	Cl_int_ [μL/min/mg]^a^	8 ± 2	14 ± 3	10 ± 2	<5
mouse plasma	*T*_1/2_ [min]^a^	>150	>150	>150	>150
	% at 2.5 h^a^	>100	>100	>100	>100
human plasma	*T*_1/2_ [min]^a^	>150	>150	>150	>150
	% at 2.5 h^a^	>100	>100	>100	>100
PPB mouse [%]		98.9 ± 0.1	98.2 ± 0.3	96.9 ± 0.1	99.7 ± 0.1
PPB human [%]		99.9 ± 0.1	98.1 ± 0.1	96.3 ± 0.3	99.7 ± 0.1
HepG2 cytotoxicity	CC_50_ [μM]^a^	29 ± 7	62 ± 15	40 ± 5	>100

a Assays were performed in duplicate as independent experiments (*n* = 2); values are shown as mean ± SD.

### Pharmacokinetic (PK) Profiling

**Figure 6:**
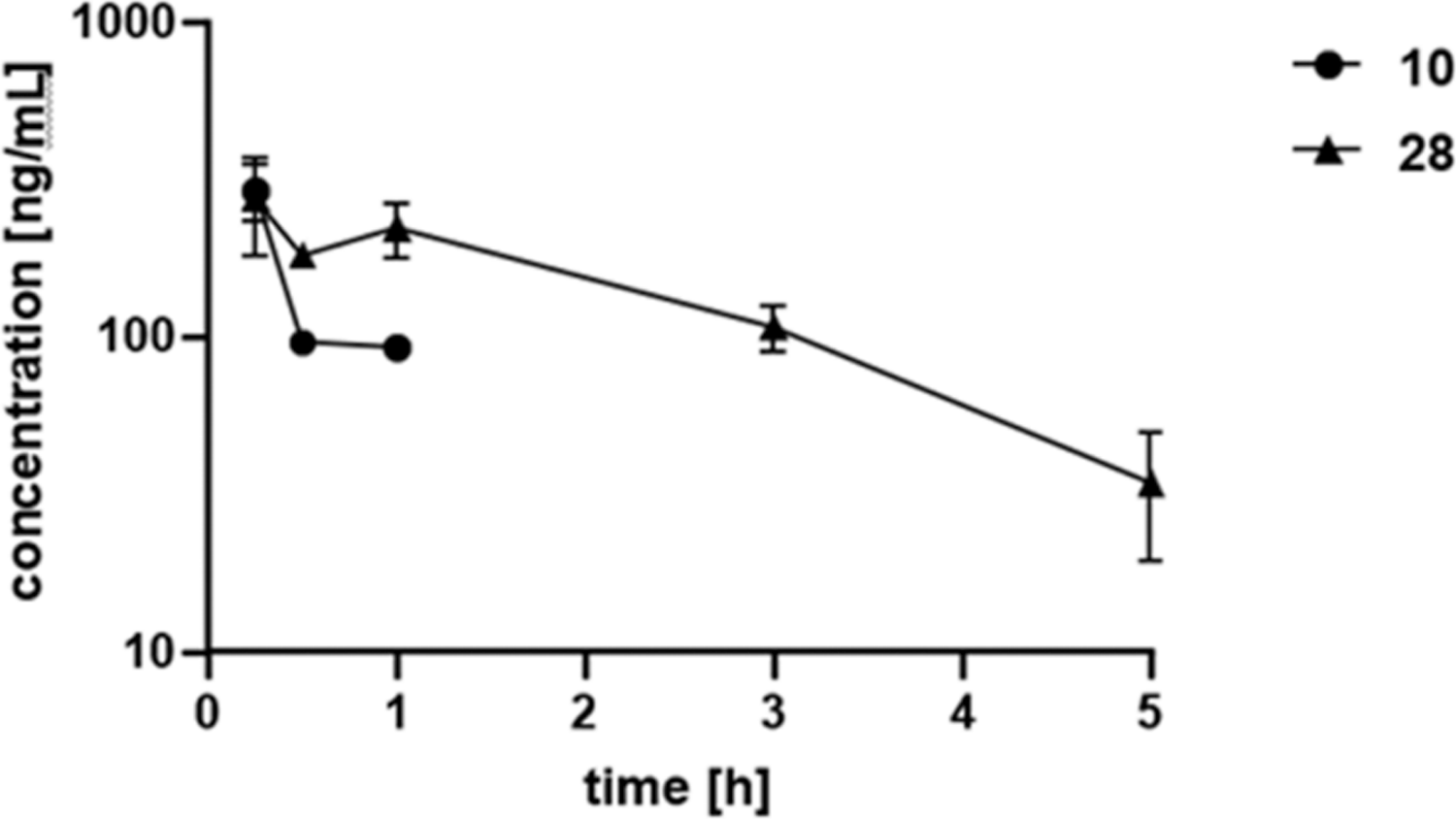
PK plasma profile over time of **10** and **28** at 1 mg/kg IV.

As several compounds exhibited promising initial ADME properties, we embarked on *in vivo* PK studies with compounds **10** and **28** in mice. We administered both compounds in a cassette format *via* the intravenous (IV) route at 1 mg/kg. Whereas compound **10** exhibited a short half-life of only 0.5 h, **28** had a half-life of around 1.6 h suggesting additional clearance mechanisms *in vivo* for compound **10** compared to **28** as the latter had a lower observed plasma clearance compared to **10**. Furthermore, both compounds had a similar volume of distribution of around 2.5–2.9 L/kg, suggesting that compounds might also distribute into tissue (Table 7). Moreover, **28** was still detectable until 5 h and had higher exposure levels (Figure 6). However, when considering the different plasma protein binding properties of compounds **10** and **28**, compound **10** exhibited an *f*AUC of around 7.71 ng/mL·h compared to **28** with an *f*AUC of around 1.86 ng/mL·h. When looking at terminal organ concentrations, **28** was found at around 203 ng/g tissue in liver, whereas **10** was not detected. This demonstrated that **28** had already favorable PK properties for further development as it distributed well to tissues. Nevertheless, it also showed that further optimization of the plasma protein binding level might be necessary.

**Table 7:** *In Vivo* PK Data for Compounds **10** and **28**

*in vivo* PK^a^	10	28
*T*_1/2_ [h]	0.05 ± 0.1	1.55 ± 0.5
*C*_0_ [ng/mL]	912.6 ± 400.2	433.1 ± 256.6
AUC_0–*t*_ [ng/mL·h]	246.4 ± 63.3	717.4 ± 135.9
MRT [h]	0.6 ± 0.2	2.24 ± 0.7
Vz_obs [L/kg]	2.5 ± 0.8	2.9 ± 1.2
Cl_obs [mL/min/kg]	53.0 ± 8.5	20.9 ± 2.0
liver ng/g	ND	202.6 ± 45.8

a Assays were performed in replicate as independent experiments (*n* = 2 mice); values are shown as mean ± SD. AUC_0–*t*_ = area under the concentration–time curve from time zero to time *t*; MRT = mean residence time; Vz_obs = observed volume of distribution; Cl_obs = observed clearance (based on observed last time point with measurable concentration); ND = not detected.

**Figure 7:**
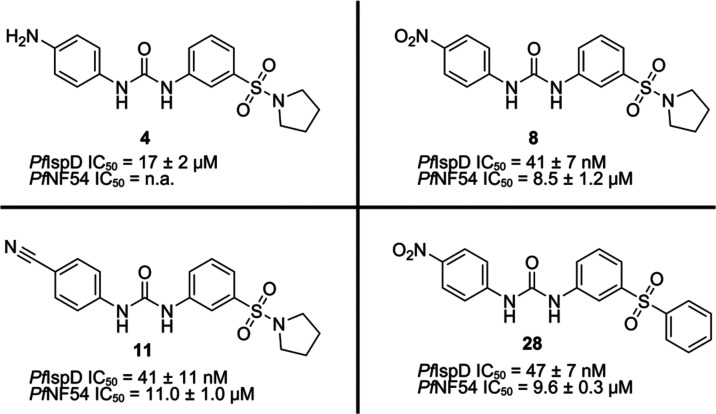
Initial hit compound and the best performing urea class derivatives. n.a. = no activity.

With our SAR, we accomplished a 400-fold increase in inhibitory activity of IspD, while also achieving activity in a whole-cell assay (Figure 7). Modifications directed to the Western side of the molecule were most impactful toward the increase in potency, and achieving whole-cell activity. Furthermore, trying to grow at this side of the molecule indicated that there is no space in the binding pocket to further expand in this direction. Adjustments directed at the urea linker, taught us that an unsubstituted urea bond is key for the activity. From there on, modifications directed to the middle ring and Eastern side of the scaffold did not result in further enhancement of the potency. Furthermore, by exploring different linkers between the middle ring and the Eastern ring, we noticed that the sulfonyl linker is essential for the potency of the compound class. Unfortunately, we observed a discrepancy between the *Pf*IspD activity and cellular activities of the compounds. Despite this, we successfully confirmed that IspD is a target for the compound class. Development of the new LC-MS based activity assay allowed us to gain an idea of the mode of inhibition of this new compound class without the use of auxiliary enzymes. Ultimately, this led to the confirmation of noncompetitive inhibition of **10** toward CTP and uncompetitive toward MEP. Therefore, the whole SAR could potentially teach us something about the structure of the allosteric pocket of *Pf*IspD. Structural information of the allosteric pocket could facilitate future research toward *Pf*IspD inhibitors. Especially as the active site of IspD appears to be challenging to target due to its polar character and solvent-exposure. Lastly, the metabolic clearance and plasma stability experiments demonstrated moderate to good values for some of the representative compounds of the urea class, which were confirmed by *in vivo* PK studies, revealing compound **28** with the best PK properties. Overall, due to its potent inhibitory activity, ease to synthesize, interesting mode of inhibition, and good ADME profile, the urea class has a great potential for further development in the anti-infective field.

## Experimental Section

### General

Purity of all compounds used in biochemical assays was ≥95%. Be aware, in contact with water, triphosgene is converted to the extremely toxic phosgene gas. Starting materials and solvents were purchased from commercial suppliers, and used without further purification. All chemical yields refer to purified compounds and were not optimized. Column chromatography was performed using the automated flash chromatography system CombiFlashRf (Teledyne Isco) equipped with RediSepRf silica columns. Preparative reversed-phase high-performance liquid chromatography (RP-HPLC) was performed either using an UltiMate 3000 Semi-Preparative System (Thermo Fisher Scientific) equipped with nucleodurC18 Gravity (250 mm × 16 mm, 5 μm) column or using a Pure C-850 Flash/Prep (Buchi) equipped with Nucleodur C18 HTec (250 mm × 40 mm, particle size 5 μm). Low-resolution mass spectrometry and purity control of final compounds was carried out using an Ultimate 3000-MSQ LCMS system (Thermo Fisher Scientific) consisting of a pump, an autosampler, MWD detector, and an ESI quadrupole mass spectrometer. ^1^H and ^13^C NMR spectra were recorded as indicated on a Bruker Avance Neo 500 MHz (^1^H, 500 MHz; ^13^C, 126 MHz) with prodigy cryoprobe system. Chemical shifts were recorded as δ values in ppm units and referenced against the residual solvent peak (DMSO-*d*_6_, δ = 2.50, 39.52 and acetone-*d*_6_: δ = 2.05, 29.84, CD_3_OD: δ = 3.27, 47.6). Splitting patterns describe apparent multiplicities and are designated as s (singlet), br s (broad singlet), d (doublet), dd (doublet of doublet), t (triplet), q (quartet), m (multiplet). Coupling constants (*J*) are given in Hertz (Hz). High-resolution mass spectra were recorded on a ThermoFisher Scientific (TF, Dreieich, Germany) Q Exactive Focus system equipped with heated electrospray ionization (HESI)-II source. For the LC-MS based IspD assay, a TF UltiMate 3000 binary RSLC UHPLC (Thermo Fisher, Dreieich, Germany) equipped with a degasser, a binary pump, an autosampler, and a thermostatted column compartment and a MWD, coupled to a TF TSQ Quantum Access Max mass spectrometer with heated electrospray ionization source (HESI-II) was used. The separation was performed with a SeQuant ZIC-HILIC 5 μM polymeric HPLC column (100 mm × 2.1 mm) with a precolumn at flow rate of 0.225 μL/min with a mobile phase composed of 50 mM ammonium acetate pH 8.5 (elute A), ACN (eluent B) under the following conditions: 0–30 s 80% B, 30–105 s 70% B, 105–135 s 70–40% B, 135–300 s hold, and 300–420 s 80% B with 225 μL/min flow rate and a total run time of 7 min. The divert valve was set to 0.49 min. The injection volume was 5 μL. The temperature of the autosampler was set to 6 °C. The following MS settings were used: electrospray ionization (ESI); negative mode for CDP-ME and MEP; collision gas pressure: 1.5 Torr; spray voltage: 10 V. The mass spectrometer was operated in the SRM mode with the following masses: 520.116 (fragment: 322.135–322.145) *m*/*z* for CDP-ME (tube lens offset 93 V and collision energy 23 eV); 215.006 (fragment: 79.395–79.405, 97.395–97.405) *m*/*z* for MEP (tube lens offset 94 V and collision energy 23 and 47 eV, respectively); 284.07 (fragment: 106.19, 177.03–150.15) *m*/*z* for 4-methyl-1-oxo-1-(*p*-tolylamino)pentane-2-sulfonic acid (tube lens offset 28 and 21 respectively V and collision energy 28 and 21 eV, respectively) with a scan width of 0.010 *m*/*z* and a scan time of 0.1 s, respectively. Observed retention times were as follows: CDP-ME, MEP, and 4-methyl-1-oxo-1-(*p*-tolylamino)pentane-2-sulfonic acid 4.90, 4.72, and 1.04 min, respectively (Figure S4). MS-peak areas were determined using TF Xcalibur Software then CDP-ME and MEP peak areas were normalized by the internal standard peak area. All PK plasma samples were analyzed via HPLC-MS/MS using an Agilent 1290 Infinity II HPLC system coupled to an AB Sciex QTrap 6500plus mass spectrometer. HPLC conditions were as follows: column: Agilent Zorbax Eclipse Plus C18, 50 mm × 2.1 mm, 1.8 μm; temperature: 30 °C; injection volume: 5 μL; flow rate: 700 μL/min; solvent A: water + 0.1% formic acid; solvent B: acetonitrile + 0.1% formic acid; gradient for 10 and 26: 99% A at 0 min, 99 – 0% A from 0.1 to 4.0 min, 0% A until 4.5 min. Mass spectrometric conditions were as follows: Scan type: Q1 and Q3 masses for glipizide, **10** and **28** can be found in Table S4; peak areas of each sample and of the corresponding internal standard were analyzed using Multi-Quant 3.0 software (AB Sciex).

### Chemistry

#### General Procedure 1 (GP-1) for the Synthesis of Analogues **5–12**

To a flask containing 3-(pyrrolidin-1-ylsulfonyl)aniline (1 equiv), and DMF (150 equiv, unless otherwise stated), the respective isocyanate (1 equiv) was added at 0 °C. The resulting mixture was stirred at room temperature overnight, after which, water was added, and the resulting mixture was extracted with EtOAc (3×, 20 mL). The combined organic layers were washed with saturated aqueous NaCl solution, dried over MgSO_4_, filtered, concentrated *in vacuo*, and purified.

#### General Procedure 2 (GP-2) for the Synthesis of Analogues **13–18**

To a flask that contains triphosgene (0.5 equiv) in DCM (150 equiv, unless otherwise stated) at 0 °C under argon atmosphere, a solution of DCM (150 equiv, unless otherwise stated) containing the respective amine (1.2 equiv) and trimethylamine (1.2 equiv) was added, and the resulting mixture was stirred at room temperature for 3 h. Next, a flask was charged with 3-(pyrrolidin-1-ylsulfonyl)aniline (1 equiv), NaH 60% (1.2 equiv) and DMF (150 equiv), the resulting mixture was stirred for 1 h under argon atmosphere, after which, the solution was added dropwise to the flask containing the triphosgene reaction mixture, and the resulting solution was stirred at room temperature overnight. Water (20 mL) was added, and the mixture was extracted with EtOAc (3×, 20 mL), washed with saturated aqueous NaCl solution, dried over MgSO_4_, filtered, concentrated *in vacuo*, and purified.

#### General Procedure 3 (GP-3) for the Synthesis of Analogues **22–29**

To a flask containing 1-isocyanato-4-nitrobenzene and DMF (150 equiv, unless otherwise stated), the respective aniline (1 equiv) was added at room temperature. The resulting mixture was stirred at room temperature overnight, after which, water was added and the resulting mixture was extracted with EtOAc (3×, 20 mL). The combined organic layers were washed with saturated aqueous NaCl solution, dried over MgSO_4_, filtered, concentrated *in vacuo*, and purified.

#### General Procedure 4 (GP-4) for the Synthesis of Analogues **30–34**

To a flask containing acetonitrile (100 equiv), trimethylamine (2 equiv), and pyrrolidine (1 equiv) at 0 °C, the respective 3-nitrobenzenesulfonyl chloride (1 equiv) was added. Next, the resulting solution was stirred at 0 °C for 5 min, after which, the solvent was evaporated. To the residue, EtOH (140 equiv), an aqueous solution of NH_4_Cl at a concentration of 166 mM (0.50 equiv), and Fe powder (5 equiv) were added, the resulting reaction mixture was stirred at 80 °C for 2.5 h. Next, the organic solvent was evaporated *in vacuo*, water (20 mL) was added, and the solution was extracted with EtOAc (3×, 20 mL). The combined organic layers were dried over MgSO_4_, filtered, and concentrated *in vacuo*. Subsequent, the residue was solubilized with DMF (45 equiv), and 1-isocyanato-4-nitrobenzene (1.5 equiv) was added. The resulting reaction mixture was stirred at room temperature for 1 h, after which, DMF was removed on reduced pressure and the residue was purified.

##### 1-(4-Aminophenyl)-3-(3-(pyrrolidin-1-ylsulfonyl)phenyl)urea (**4**)

A mixture of 1-(4-nitrophenyl)-3-(3-(pyrrolidin-1-ylsulfonyl)phenyl)urea (**8**) (0.15 g, 0.4 mmol), Fe (0.11 mg, 1.9 mmol), and ammonium chloride (0.01 g, 0.2 mmol) was dissolved in an ethanol/water (2:1) mixture. The mixture was heated to 100◦C for 2 h. Excess ethanol was evaporated *in vacuo*, and the remaining residue was washed with water (3×, 20 mL), and then filtered. The obtained solid was then purified using preparative HPLC affording **4** as a white powder (0.1 g, 72% yield).

^1^H NMR (500 MHz, DMSO-*d*_6_) δ 10.60 (s, 1H), 10.51 (s, 1H), 8.22 (d, *J* = 9.1, 2H), 8.08 (s, 1H), 7.82 (d, *J* = 9.1, 2H), 7.75 (d, *J* = 7.9, 1H), 7.61 (t, *J* = 7.8, 1H), 7.56 (d, *J* = 7.8, 1H), 3.17 (t, *J* = 6.6, 4H), 1.66 (t, *J* = 6.6, 4H). ^13^C NMR (126 MHz, DMSO-*d*_6_) δ 179.7, 145.8, 142.6, 140, 136.2, 129.7, 127.5, 124.5, 123.3, 122.2, 121.8, 47.9, 24.8. High-resolution mass spectrometry (HR-MS) (ESI) calculated for C_17_H_21_N_4_O_3_S [M + H]^+^: 361.1256, found: 361.1327

##### 1-(4-Chlorophenyl)-3-(3-(pyrrolidin-1-ylsulfonyl)phenyl)urea (**5**)

According to **GP-1**, using 1-chloro-4-isocyanatobenzene (0.08 g, 0.49 mmol), affording after purification by flash chromatography (CH_2_Cl_2_/MeOH, 10/0 → 9.5/0.5), and washing with MeOH, **5** was afforded as a white powder (22 mg, 11% yield). ^1^H NMR (500 MHz, DMSO-*d*_6_) δ 9.1 (s, 2H), 8.1 (t, *J* = 2.0, 1H), 7.7–7.6 (m, 1H), 7.6–7.5 (m, 3H), 7.4 (d, *J* = 7.7, 1H), 7.4–7.3 (m, 2H), 3.2–3.1 (m, 4H), 1.7–1.6 (m, 4H). ^13^C NMR (126 MHz, DMSO-*d*_6_) δ 152.9, 141, 138.9, 137, 130.3, 129.1, 126.1, 122.7, 120.8, 120.5, 116.9, 48.3, 25.2. HR-MS (ESI) calculated for C_17_H_19_ClN_3_O_3_S [M + H]^+^, 380.0757, found: 380.0822. HPLC purity: 98%.

##### 1-(3-(Pyrrolidin-1-ylsulfonyl)phenyl)-3-(*p*-tolyl)urea (**6**)

*A*ccording to **GP-1**, using *p*-tolyl isocyanate (0.06 mL, 0.48 mmol), afforded after purification by flash chromatography (cyclohexane/EtoAc = 1:1) **6** as white solid (0.15 g, 95% yield). ^1^H NMR (500 MHz, DMSO-*d*_6_) δ 9.0 (s, 1H), 8.7 (s, 1H), 8.1 (t, *J* = 2.0, 1H), 7.6–7.5 (m, 1H), 7.5 (t, *J* = 7.9, 1H), 7.4–7.3 (m, 3H), 7.1 (d, *J* = 8.3, 2H), 3.2–3.1 (m, 4H), 2.3 (s, 3H), 1.7–1.6 (m, 4H). ^13^C NMR (126 MHz, DMSO-*d*_6_) δ 153, 137.2, 137, 131.5, 130.2, 129.7, 122.5, 120.6, 119.1, 116.7, 48.3, 25.2, 20.8. HR-MS (ESI) calculated for C_18_H_22_N_3_O_3_S [M + H]^+^, 360.1304, found: 360.1361. HPLC purity: 99%.

##### Methyl 4-(3-(3-(Pyrrolidin-1-ylsulfonyl)phenyl)ureido)benzoate (**7**)

According to **GP-1**, using methyl 4-isocyanatobenzoate (0.088 g, 0.5 mmol), and CH_2_Cl_2_ (10 mL), to afford after filtration of the precipitate, **7** as a white solid (0.15 g, 75% yield). ^1^H NMR (500 MHz, DMSO-*d*_6_) δ 9.2 (br s, 2H), 8.1 (t, *J* = 1.8 Hz, 1H), 7.9 (d, *J* = 8.7 Hz, 2H), 7.7–7.6 (m, 3H), 7.6 (t, *J* = 7.9 Hz, 1H), 7.4 (d, *J* = 7.6 Hz, 1H), 3.8 (s, 3H), 3.2–3.1 (m, 4H), 1.7–1.6 (m, 4H). ^13^C NMR (126 MHz, DMSO-*d*_6_) δ 166.4, 152.7, 144.5, 140.7, 137.1, 130.9, 130.4, 123.2, 122.8, 121.1, 118.1, 117.1, 52.3, 48.3, 25.2. HR-MS (ESI) calculated for C_19_H_22_N_3_O_5_S [M + H]^+^: 404.1202, found: 404.1275. HPLC purity: 98%

##### 1-(4-Nitrophenyl)-3-(3-(pyrrolidin-1-ylsulfonyl)phenyl)urea (**8**)

According to **GP-1**, using 1-isocyanato-4-nitrobenzene (0.1 g, 0.61 mmol), to afford after purification by flash chromatography (CH_2_Cl_2_/MeOH, 10/0 → 9.5/0.5), **8** as a yellow powder (0.02 g, 8% yield). ^1^H NMR (500 MHz, DMSO-*d*_6_) δ 9.6 (br s, 1H), 9.4 (br s, 1H), 8.2 (br d, *J* = 8.9 Hz, 2H), 8.1 (br s, 1H), 7.7 (br d, *J* = 8.4 Hz, 2H), 7.7 (br d, *J* = 7.6 Hz, 1H), 7.6 (br t, *J* = 7.9 Hz, 1H), 7.4 (br d, *J* = 7.6 Hz, 1H), 3.2–3.1 (m, 4H), 1.7 (br s, 4H). ^13^C NMR (126 MHz, DMSO-*d*_6_) δ 152.1, 146.1, 141.2, 140, 136.6, 129.9, 125.1, 122.5, 120.9, 117.9, 117.8, 116.8, 47.9, 24.7. HR-MS (ESI) calculated for C_17_H_19_N_4_O_5_S [M + H]^+^: 391.0998, found: 391.1066. HPLC purity: 100%.

##### 1-(4-(Methylsulfonyl)phenyl)-3-(3-(pyrrolidin-1-ylsulfonyl)phenyl)urea (**9**)

According to **GP-1**, using 1-isocyanato-4-(methylsulfonyl)benzene (0.99 g, 0.5 mmol) and, DCM (10 mL), to afford after filtration of the precipitate **9** as a white powder (0.15 g, 70% yield). ^1^H NMR (500 MHz, DMSO-*d*_6_) δ 9.29 (br s, 2H), 8.1–8.0 (m, 1H), 7.8 (d, *J* = 8.9 Hz, 2H), 7.7 (d, *J* = 8.9 Hz, 2H), 7.7–7.6 (m, 1H), 7.5 (t, *J* = 7.9 Hz, 1H), 7.4 (d, *J* = 7.8 Hz, 1H), 3.2–3.1 (m, 7H), 1.7–1.6 (m, 4H). ^13^C NMR (126 MHz, DMSO-*d*_6_) δ 152.7, 144.7, 140.6, 137.1, 133.9, 130.4, 128.8, 122.9, 121.2, 118.5, 117.2, 48.3, 44.4, 25.2. HR-MS (ESI) calculated for C_18_H_22_N_3_O_5_S_2_ [M + H]^+^: 424.0923, found: 424.0997. HPLC purity: 99%.

##### 1-(4-Cyanophenyl)-3-(3-(pyrrolidin-1-ylsulfonyl)phenyl)urea (**10**)

According to **GP-1**, using 4-isocyanatobenzonitrile (0.1 g, 0.69 mmol), to afford after purification by flash chromatography (CH_2_Cl_2_/MeOH, 10/0 → 9.5/0.5) and recrystallization with CH_2_Cl_2_, and diethyl ether, **10** as a yellow powder (0.075 g, 29% yield). ^1^H NMR (500 MHz, DMSO-*d*_6_) δ 9.3 (br s, 2H), 8.1 (t, *J* = 1.8 Hz, 1H), 7.8–7.7 (m, 2H), 7.7–7.6 (m, 2H), 7.7–7.6 (m, 1H), 7.6 (t, *J* = 7.9 Hz, 1H), 7.4 (d, *J* = 7.8 Hz, 1H), 3.2–3.1 (m, 4H), 1.7–1.64 (m, 4H). ^13^C NMR (126 MHz, DMSO-*d*_6_) δ 152.6, 144.4, 140.6, 137.1, 133.8, 130.4, 123, 121.3, 119.7, 118.8, 117.2, 104.1, 49.1, 48.3, 25.2. HR-MS (ESI) calculated for C_18_H_19_N_4_O_3_S [M + H]^+^: 371.1010, found: 371.1157. HPLC purity: 98%.

##### 1-(3-(Pyrrolidin-1-ylsulfonyl)phenyl)-3-(4-(trifluoromethyl)phenyl)urea (**11**)

According to **GP-1**, using 1-isocyanato-4-(trifluoromethyl)benzene (0.09 g, 0.5 mmol), and DCM (10 mL), to afford after filtration of the precipitate, **11** as an off-white powder (0.09 g, 0.213 mmol, 43% yield). ^1^H NMR (500 MHz, DMSO-*d*_6_) δ 9.2 (s, 1H), 8.1 (t, *J* = 1.8 Hz, 1H), 7.7–7.6 (m, 1H), 7.6–7.5 (m, 1H), 7.5 (t, *J* = 7.9 Hz, 1H), 7.4 (d, *J* = 7.8 Hz, 1H), 3.2–3.1 (m, 1H), 1.7–1.6 (m, 1H). ^13^C NMR (126 MHz, DMSO-*d*_6_) δ 152.8, 143.6, 140.7, 137.1, 130.4, 126.7–126.5 (m), 122.9, 121.1, 118.7, 117.1, 48.3, 25.2. ^19^F NMR (470 MHz DMSO-*d*_6_) δ −60.1. HR-MS (ESI) calculated for C_18_H_19_F_3_N_3_O_3_S [M + H]^+^: 414.1021, found: 414.1087. HPLC purity: 99%.

##### 1-(3-(Pyrrolidin-1-ylsulfonyl)phenyl)-3-(3-(trifluoromethyl)phenyl)urea (**12**)

According to **GP-1**, using 1-isocyanato-3-(trifluoromethyl)benzene (0.07 g, 0.35 mmol), to afford after purification by flash chromatography (EtOAc/petroleum ether, 3/7 → 5/5), recrystallization using MeOH, and washing with CH_2_Cl_2_ (3×, 5 mL), **12** as a white crystalline powder (0.01 g, 8% yield). ^1^H NMR (500 MHz, CD_3_OD) δ 8.1 (t, *J* = 1.9 Hz, 1H), 7.9 (s, 1H), 7.7 (ddd, *J* = 8.0, 2.2, 1.2 Hz, 1H), 7.6 (dd, *J* = 8.2, 1.6 Hz, 1H), 7.6–7.5 (m, 1H), 7.5–7.4 (m, 2H), 7.3–7.2 (m, 1H), 3.3–3.2 (m, 4H), 1.8–1.7 (m, 4H). ^13^C NMR (126 MHz, CD_3_OD) δ 153.3, 140.2, 140, 137.2, 129.4 (d, *J* = 4.4), 125.3, 123.1, 122.7, 122, 121.1, 118.8, 117.4, 115.2, 47.8, 24.8. ^19^F NMR (470 MHz DMSO-*d*_6_) δ −61.3. HR-MS (ESI) calculated for C_18_H_19_F_3_N_3_O_3_S [M + H]^+^: 414.1021, found: 414.1088. HPLC purity: 98%.

##### 1-(3,5-Dichlorophenyl)-3-(3-(pyrrolidin-1-ylsulfonyl)phenyl)urea (**13**)

According to **GP-2**, using 1,3-dichloro-5-isocyanatobenzene (0.09 g, 0.48 mmol) to afford **13** after evaporation of the solvent as white solid (0.14 g, 77%). ^1^H NMR (500 MHz, DMSO-*d*_6_) δ 9.4–9.1 (m, 2H), 8.1 (t, *J* = 1.8 Hz, 1H), 7.7–7.6 (m, 1H), 7.6–7.5 (m, 3H), 7.4 (d, *J* = 7.8 Hz, 1H), 7.2 (t, *J* = 1.8 Hz, 1H), 3.2–3.1 (m, 4H), 1.7–1.6 (m, 4H). ^13^C NMR (126 MHz, DMSO-*d*_6_) δ 152.7, 142.5, 140.6, 137.1, 134.6, 130.4, 123, 121.7, 121.2, 117.2, 117.1, 48.3, 25.2. HR-MS (ESI) calculated for C_17_H_18_Cl_2_N_3_O_3_S [M + H]^+^: 414.0368, found: 414.0430. HPLC purity: 99%

##### 1-(2-Fluoro-4-(trifluoromethyl)phenyl)-3-(3-(pyrrolidin-1-ylsulfonyl)phenyl)urea (**14**)

According to **GP-2**, 2-fluoro-4-(trifluoromethyl)aniline (0.14 g, 0.79 mmol), to afford after purification by flash chromatography (cyclohexane/EtoAc = 1:1), **14** as white powder (0.04 g, 15% yield). ^1^H NMR (500 MHz, DMSO-*d*_6_) δ 9.6 (br s, 1H), 9.0 (br s, 1H), 8.4 (t, *J* = 8.3 Hz, 1H), 8.1 (s, 1H), 7.8–7.7 (m, 1H), 7.6–7.5 (m, 3H), 7.5–7.4 (m, 1H), 3.2–3.1 (m, 4H), 1.7–1.6 (m, 4H). ^13^C NMR (126 MHz, DMSO-*d*_6_) δ 151.9, 151.7, 145.0, 139.7, 136.5, 129.9, 122.1, 121.9–121.6, 120.7, 120.1, 116.2, 47.7, 24.5. ^19^F NMR (470 MHz DMSO-*d*_6_) δ −60.2, −127.7. HR-MS (ESI) calculated for C_18_H_18_F_4_N_3_O_3_S [M + H]^+^: 432.0927, found: 432.0995. HPLC purity: 99%.

##### 1-(Naphthalen-2-yl)-3-(3-(pyrrolidin-1-ylsulfonyl)phenyl)urea (**15**)

According to **GP-2**, naphthalen-2-amine (0.1 g, 0.70 mmol), to afford after washing with MeOH (5×, 5 mL), **15** as an off-white solid (0.02 g, 8% yield). ^1^H NMR (500 MHz, DMSO-*d*_6_) δ 9.2 (s, 1 H), 9.0 (s, 1 H), 8.2 (s, 1 H), 8.1 (s, 1 H), 7.9 (br d, *J* = 8.85, 2 H), 7.8 (br d, *J* = 9.77, 1 H), 7.6 (d, *J* = 8.06, 1 H), 7.6 (t, *J* = 7.93, 1 H), 7.5 (dd, *J* = 8.77, 2.06, 1 H), 7.5 (t, J = 7.48, 1 H), 7.4–7.3 (m, 2 H), 3.2–3.1 (m, 4 H), 1.7–1.6 (m, 4 H). ^13^C NMR (126 MHz, DMSO-*d*_6_) δ 152.7–152.8, 152.7, 140.7, 138.9–142.1, 137.2, 136.7, 133.8, 130, 129.4, 128.6, 127.6, 127.2, 126.6, 124.3, 122.3, 120.5, 120, 116.5, 114, 48, 24. HR-MS (ESI) calculated for C_21_H_22_N_3_O_3_S [M + H]^+^: 396,1304, found: 396.1364. HPLC purity: 99%.

##### 1-(4-Phenoxyphenyl)-3-(3-(pyrrolidin-1-ylsulfonyl)phenyl)urea (**16**)

According to **GP-2**, using 4-phenoxyaniline (0.17 g, 0.94 mmol), to afford after purification with flash chromatography (cyclohexane/EtOAc = 7:3) **16** as a white solid (0.18 g, 44%). ^1^H NMR (500 MHz, DMSO-*d*_6_) δ 9.1 (s, 1H), 8.8 (s, 1H), 8.1 (t, *J* = 2.0, 1H), 7.6 (ddd, *J* = 8.2, 2.3, 1.0, 1H), 7.5 (t, *J* = 7.9, 1H), 7.5–7.4 (m, 2H), 7.4–7.3 (m, 3H), 7.1 (t, *J* = 7.5, 1H), 7.0 (m, 4H), 3.2–3.1 (m, 4H), 1.7–1.6 (m, 4H). ^13^C NMR (126 MHz, DMSO-*d*_6_) δ 158.1, 153.1, 151.4, 141.2, 137, 135.8, 130.4, 130.3, 123.3, 122.6, 120.9, 120.7, 120.2, 118.1, 116.8, 48.3, 25.2. HR-MS (ESI) calculated for C_23_H_24_N_3_O_4_S [M + H]^+^: 438.1409, found: 438.1474. HPLC purity: 99%.

##### 1-(3-(Morpholinomethyl)phenyl)-3-(3-(pyrrolidin-1-ylsulfonyl)phenyl)urea (**17**)

According to **GP-2**, 3-(morpholinomethyl)aniline (0.1 g, 0.52 mmol), to afford after washing with MeOH (5×, 5 mL), **17** as a yellow crystalline powder (0.09 g, 31% yield). ^1^H NMR (500 MHz, DMSO-*d*_6_) δ 9.1 (s, 1 H), 8.8 (s, 1 H), 8.1 (t, *J* = 1.91, 1 H), 7.6 (dd, *J* = 8.16, 1.14, 1 H), 7.5 (t, *J* = 7.93, 1 H), 7.4 (s, 1 H), 7.4 – 7.3 (m, 2 H), 7.3–7.2 (m, 1 H), 7.0–6.9 (m, 1 H), 3.6 (t, *J* = 4.50, 4 H), 3.4 (s, 2 H), 3.2–3.1 (m, 4 H), 2.4 (br s, 4 H), 1.7–1.6 (m, 4 H). ^13^C NMR (126 MHz, DMSO-*d*_6_) δ 152.4, 140.6, 139.3, 138.6, 136.5, 129.8, 128.6, 122.8, 122.1, 120.2, 118.8, 117.2, 116.3, 66.2, 62.5, 53.2, 47.8, 24.7. HR-MS (ESI) calculated for C_22_H_29_N_4_O_4_S [M + H]^+^: 445,1831, found: 445,1899. HPLC purity: 98%.

##### 1-(3-((1*H*-Imidazol-1-yl)methyl)phenyl)-3-(3-(pyrrolidin-1-ylsulfonyl)phenyl)urea (**18**)

According to **GP-2**, using 3-((1*H*-imidazol-1-yl)methyl)aniline (0.1 g, 0.58 mmol), to afford after washing with MeOH (5×, 5 mL), **18** as a white solid (0.01 g, 4% yield). ^1^H NMR (500 MHz, DMSO-*d*_6_) δ 9.2 (br s, 1H), 8.9 (br s, 1H), 8.1 (s, 1H), 7.8 (s, 1H), 7.6–7.5 (m, 1H), 7.5 (t, *J* = 7.9 Hz, 1H), 7.4–7.3 (m, 2H), 7.4–7.3 (m, 1H), 7.3 (t, *J* = 7.9 Hz, 1H), 7.2–7.1 (m, 1H), 6.9 (s, 1H), 6.9–6.8 (m, 1H), 5.2 (s, 2H), 3.1 (br t, *J* = 6.6 Hz, 4H), 1.7–1.6 (m, 4H). ^13^C NMR (126 MHz, DMSO-*d*_6_) δ 152.4, 140.5, 139.7, 138.4, 136.4, 129.7, 129, 122, 120.9, 120.1, 117.6, 117.1, 116.1, 49.4, 47.7, 24.6. HR-MS (ESI) calculated for C_21_H_24_N_5_O_3_S [M + H]^+^: 426,1522, found: 426.1582. HPLC purity: 98%.

##### 1-Methyl-1-(4-nitrophenyl)-3-(3-(pyrrolidin-1-ylsulfonyl)phenyl)urea (**20**)

To a flask containing triphosgene (0.08 g, 0.27 mmol), and DCM (2 mL) at 0 °C under argon atmosphere, a solution containing 3-((1*H*-imidazol-1-yl)methyl)aniline (0.13 g, 0.89 mmol), trimethylamine (247 mL, 1.78 mmol), and DCM (2 mL), was added. The resulting solution was stirred at room temperature for 2 h. To a different flask, *N*-methyl-4-nitroaniline (0.13 g, 0.89 mmol), sodium hydride (0.03 mg, 1.19 mmol), and DMF (2.5 mL) were added, the resulting solution was stirred at room temperature for 2 h, after which, it was added dropwise to the solution containing triphosgene. The resulting mixture was stirred at room temperature for 1 h, next water was added and the mixture was extracted with EtOAc (5×, 20 mL). The combined organic layers were washed with saturated aqueous NaCl solution, dried over MgSO_4_, filtered, concentrated *in vacuo*, and purified by column chromatography, (CH_2_Cl_2_/MeOH, 10/0 → 9.5/0.5), and recrystallization (CH_2_Cl_2_/diethyl ether), to afford **19** as a yellow solid (0.12 g, 32% yield). ^1^H NMR (500 MHz, DMSO-*d*_6_) δ 9.2 (s, 1H), 8.3–8.2 (m, 1H), 8.0 (t, *J* = 1.9 Hz, 1H), 7.8 (dd, *J* = 8.1, 1.4 Hz, 1H), 7.7–7.6 (m, 1H), 7.5 (t, *J* = 8.0 Hz, 1H), 7.4 (d, *J* = 7.8 Hz, 1H), 3.4 (s, 1H), 3.2–3.1 (m, 1H), 1.7–1.6 (m, 1H). ^13^C NMR (126 MHz, DMSO-*d*_6_) δ 154.7, 150.6, 143.9, 141.1, 136.7, 130, 125.4, 124.9, 124.3, 121.5, 118.8, 48.3, 37.4, 25.2. HR-MS (ESI) calculated for: C_18_H_21_N_4_O_5_S [M + H]^+^: 405.1154, found: 405.1214. HPLC purity: 99%.

##### 1-Methyl-3-(4-nitrophenyl)-1-(3-(pyrrolidin-1-ylsulfonyl)phenyl)urea (**21**)

A flask was charged with 3-(pyrrolidin-1-ylsulfonyl)aniline (0.1 g, 0.44 mmol), paraformaldehyde (0.09 g), and MeOH (5 mL). The resulting solution was stirred at room temperature for 2.5 h, after which, NaBH_4_ (0.03 g, 0.88 mmol) was added. The resulting mixture was stirred at 60 °C for 16 h, next, water (20 mL) was added, and the mixture was extracted with EtOAc (5×, 20 mL). The combined organic layers were washed with saturated aqueous NaCl solution, dried over MgSO_4_, filtered, concentrated *in vacuo*, and purified by flash chromatography (EtOAc/petroleum benzyne 3/7 → 6/4) affording *N*-methyl-3-(pyrrolidin-1-ylsulfonyl)aniline (**ii**) (0.06 g, 0.25 mmol, 57% yield) which was added to a flask containing 1-isocyanato-4-nitrobenzene (0.07 g, 0.28 mmol), and DMF (5 mL). The solution was stirred at room temperature overnight, next, water (20 mL) was added, and the resulting mixture was extracted with EtOAc (5×, 20 mL). The combined organic layers were washed with saturated aqueous NaCl solution, dried over MgSO_4_, filtered, concentrated *in vacuo*, purified by flash chromatography (CH_2_Cl_2_/MeOH, 10/0 → 9.5/0.5), and recrystallized (MeOH, CH_2_Cl_2_ and diethyl ether) to afford **20** as an off-white solid (0.02 g, 12% yield). ^1^H NMR (500 MHz, DMSO-*d*_6_) δ 9.2 (br s, 1H), 8.2 (d, *J* = 9.2 Hz, 2H), 7.8–7.6 (m, 6H), 3.4 (s, 3H), 3.2–3.1 (m, 4H), 1.7–1.6 (m, 4H) ^13^C NMR (126 MHz, DMSO-*d*_6_) δ 154.5, 147.4, 144.9, 141.6, 137.2, 130.7 (d, *J* = 10.9), 125.1, 124.9, 119.1, 48.3, 38.1, 25.1. HR-MS (ESI) calculated for: C_18_H_21_N_4_O_5_S [M + H]^+^: 405.1154, found: 405.1215. HPLC purity: 100%.

##### 1-(4-Nitrophenyl)-3-(3-(pyrrolidin-1-ylsulfonyl)phenyl)thiourea (**23**)

To 3-(pyrrolidin-1-ylsulfonyl)aniline (0.03 g, 0.11 mmol) dissolved in DCM (5 mL) was added 1-isothiocyanato-4-nitrobenzene (0.02 g, 0.11 mmol) at 0 °C. The reaction was then stirred at room temperature for 2 days. The reaction was quenched by the addition of saturated aqueous solution of NaHCO_3_ (20 mL), and extracted with DCM. The organic solvent was dried over MgSO_4_, filtered, and then removed *in vacuo* and the reaction was purified using preparative HPLC affording **21** (0.02 g, 35% yield). ^1^H NMR (500 MHz, DMSO-*d*_6_) δ 9.0 (s, 1H), 7.9 (t, *J* = 1.8 Hz, 1H), 7.8 (d, *J* = 8.5 Hz, 2H), 7.8 (dd, *J* = 8.2, 1.3 Hz, 1H), 7.6 (d, *J* = 8.4 Hz, 2H), 7.5 (t, *J* = 8.0 Hz, 1H), 7.4–7.3 (m, 1H), 3.2–3.1 (m, 4H), 1.7–1.62 (m, 4H). ^13^C NMR (126 MHz, DMSO-*d*_6_) δ 153.7, 145.4, 140.5, 136, 129.5, 127.3, 126.5, 123.9, 121, 118.4, 80.1, 75.1, 47.9, 24.7. HR-MS (ESI) calculated for C_17_H_19_N_4_O_4_S_2_ [M + H]^+^: 407.0770, found: 407.0839. HPLC purity: 95%.

##### *N*,*N*-Dimethyl-3-(3-(4-nitrophenyl)ureido)benzenesulfonamide (**24**)

According to **GP-3** using, 3-amino-*N*,*N*-dimethylbenzenesulfonamide (0.1 g, 0.5 mmol), and DCM (10 mL), to afford after filtration of the precipitate, **22** as a white solid (0.09 g, 49% yield). ^1^H NMR (500 MHz, DMSO-*d*_6_) δ 9.6–9.3 (m, 2H), 8.2–8.1 (m, 2H), 8.1 (t, *J* = 1.8 Hz, 1H), 7.8–7.7 (m, 2H), 7.6 (dd, *J* = 7.9, 1.5 Hz, 1H), 7.6 (t, *J* = 7.9 Hz, 1H), 7.4 (d, *J* = 7.8 Hz, 1H), 2.7–2.6 (m, 6H). ^13^C NMR (126 MHz, DMSO-*d*_6_) δ 152.5, 146.6, 141.7, 140.5, 135.7, 130.4, 125.6, 123.2, 121.7, 118.3, 117.5, 38.1. HR-MS (ESI) calculated for C_15_H_17_N_4_O_5_S [M + H]^+^: 365.0841, found: 365.0915. HPLC purity: 99%.

##### *N*,*N*-Diethyl-3-(3-(4-nitrophenyl)ureido)benzenesulfonamide (**25**)

According to **GP-3** using, 3-amino-*N*,*N*-diethylbenzenesulfonamide (0.114 g, 0.5 mmol), and DCM (10 mL), to afford after filtration of the precipitate, **23** as a yellow solid (0.11 g, 58% yield). ^1^H NMR (500 MHz, DMSO-*d*_6_) δ 9.4 (br s, 2H), 8.2–8.1 (m, *J* = 9.2 Hz, 2H), 8.1–8.0 (m, 1H), 7.8–7.7 (m, 2H), 7.6 (dd, *J* = 8.2, 1.0 Hz, 1H), 7.5 (t, *J* = 7.9 Hz, 1H), 7.4 (d, *J* = 7.8 Hz, 1H), 3.2 (q, *J* = 7.2 Hz, 4H), 1.1 (t, *J* = 7.2 Hz, 6H) ^13^C NMR (126 MHz, DMSO-*d*_6_) δ 152.5, 146.6, 141.7, 140.8, 140.5, 130.4, 125.6, 122.7, 120.8, 118.3, 116.7, 42.3, 14.6. HR-MS (ESI) calculated for C_17_H_21_N_4_O_5_S [M + H]^+^: 393.1154, found: 393.1221. HPLC purity: 99%.

##### 1-(4-Nitrophenyl)-3-(3-(piperidin-1-ylsulfonyl)phenyl)urea (**26**)

According to **GP-3** using, 3-(piperidin-1-ylsulfonyl)aniline (0.120 g, 0.5 mmol), and DCM (10 mL), to afford after filtration of the precipitate, **24** as an off-white solid (0.112 g, 55% yield). ^1^H NMR (500 MHz, DMSO-*d*_6_) δ 9.6–9.3 (m, 2H), 8.2–8.1 (m, 2H), 8.1–8.0 (m, 1H), 7.8–7.7 (m, 2H), 7.7 (dd, *J* = 7.9, 1.6 Hz, 1H), 7.6 (t, *J* = 7.9 Hz, 1H), 7.4–7.3 (m, 1H), 2.9–2.8 (m, 4H), 1.6–1.5 (m, 4H), 1.4–1.3 (m, 2H) ^13^C NMR (126 MHz, DMSO-*d*_6_) δ 152.5, 146.5, 141.7, 140.5, 136.6, 130.4, 125.6, 123.1, 121.6, 118.3, 117.3, 47.1, 25.2, 23.3. HR-MS (ESI) calculated for C_18_H_21_N_4_O_5_S [M + H]^+^: 405.1154, found: 405.1213. HPLC purity: 99%.

##### 1-(3-(Morpholinosulfonyl)phenyl)-3-(4-nitrophenyl)urea (**27**)

According to **GP-3** using, (3-orpholinosulfonyl)aniline (0.12 g, 0.5 mmol), and CH_2_Cl_2_ (5 mL), to afford after filtration of the precipitate, **25** as a white powder (0.06 g, 31% yield). ^1^H NMR (500 MHz, DMSO-*d*_6_) δ 9.6–9.3 (m, 2H), 8.2–8.1 (m, 2H), 8.1–8.0 (m, 1H), 7.8–7.7 (m, 2H), 7.7–7.6 (m, 1H), 7.6–7.5 (m, 1H), 7.4 (d, *J* = 7.6 Hz, 1H), 3.7–3.6 (m, 4H), 2.9–2.8 (m, 4H). ^13^C NMR (126 MHz, DMSO-*d*_6_) δ 152.6, 141.7, 140.6, 135.4, 130.5, 125.6, 123.5, 121.8, 118.3, 117.5, 65.8, 46.4. HR-MS (ESI) calculated for C_17_H_19_N_4_O_6_S [M + H]^+^: 407.0947, found: 407.1006. HPLC purity: 99%.

##### 1-(4-Nitrophenyl)-3-(3-(phenylsulfonyl)phenyl)urea (**28**)

According to **GP-3** 3-(pyrrolidin-1-ylsulfonyl)aniline (0.16 g, 0.69 mmol) to afford after purification by flash chromatography (CH_2_Cl_2_/MeOH, 10/0 → 9.5/0.5), and washing the residue with MeOH, and CH_2_Cl_2_, **26** as a yellow powder (0.08 g, 28% yield). ^1^H NMR (500 MHz, DMSO-*d*_6_) δ 9.7–9.6 (m, 1H), 9.5–9.4 (m, 1H), 8.3–8.2 (m, 1H), 8.2–8.1 (m, 2H), 8.0–7.9 (m, 2H), 7.8–7.7 (m, 3H), 7.7–7.5 (m, 5H). ^13^C NMR (126 MHz, DMSO-*d*_6_) δ 152.5, 146.5, 142.1, 141.8, 141.5, 140.8, 134.3, 130.9, 130.3, 127.8, 125.6, 123.8, 121.5, 118.5, 118.3, 117. HR-MS (ESI) calculated for: C_19_H_16_N_3_O_5_S [M + H]^+^: 398.0732, found: 398.0800. HPLC purity: 98%.

##### 1-(4-Nitrophenyl)-3-(3-(pyrrolidine-1-carbonyl)phenyl)urea (**29**)

To a flask containing 3-nitrobenzoic acid (0.25 g, 1.5 mmol), pyrrolidine (0.21 g, 3.0 mmol), trimethylamine (0.33 g, 3.3 mmol) and DCM (2 mL), a solution of propanephosphonic acid anhydride in EtOAc (50%, 1.4 g, 2.2 mmol) was added. The resulting solution was stirred at room temperature for 2 h, after which, water was added, and the solution was extracted with DCM (3×, 15 mL). The combined organic layers were dried over MgSO_4_, filtered, and concentrated *in vacuo*. To the residue, EtOH (5 mL), an aqueous solution of NH_4_Cl at a concentration of 166 mM in water (3.83 mL, 0.64 mmol) and Fe powder (0.18 g, 3.2 mmol) were added, the resulting reaction mixture was stirred at 80 °C for 2.5 h. Next, the organic solvent was evaporated *in vacuo*, water (20 mL) was added, and the solution was extracted with DCM (3×, 15 mL). The combined organic layers were dried over MgSO_4_, filtered, and concentrated *in vacuo*. Subsequent, the residue was solubilized with DCM (15 mL), and 1-isocyanato-4-nitrobenzene (0.15 g, 0.89 mmol) was added. The resulting reaction mixture was stirred at room temperature for 1 h, after which, DMF was removed under reduced pressure, and the residue was purified using preparative RP-HPLC to yield **29** as a yellow solid (0.04 g, 9% yield). ^1^H NMR (500 MHz, DMSO-*d*_6_) δ 9.5 (br s, 1H), 9.1 (br s, 1H), 8.2 (d, *J* = 9.0 Hz, 2H), 7.7–7.6 (m, 3H), 7.5–7.4 (m, 1H), 7.4 (t, J = 7.8 Hz, 1H), 7.1 (d, *J* = 7.5 Hz, 1H), 3.5 (t, *J* = 6.9 Hz, 2H), 3.4 (t, *J* = 6.4 Hz, 2H), 1.9–1.8 (m, 4H). ^13^C NMR (126 MHz, DMSO-*d*_6_) δ 168.0, 152.0, 146.3, 141.1, 138.9, 137.8, 128.8, 125.1, 121.0, 119.8, 117.9, 117.2, 49.0, 45.9, 26.0, 23.9. HR-MS (ESI) calculated for: C_18_H_19_N_4_O_4_ [M + H]^+^: 355.1328, found: 355.14202. HPLC purity: 100%

##### 1-(4-Nitrophenyl)-3-(3-(pyrrolidin-1-ylmethyl)phenyl)urea (**30**)

To a flask containing 3-nitrobenzyl bromide (0.2 g, 0.92 mmol), trimethylamine (0.09 g, 0.92 mmol) and DCM (2.5 mL), pyrrolidine (0.07 g, 0.92 mmol) was added. Next the solution was stirred at room temperature for 2 h, after which, water was added and the resulting solution was extracted with DCM (3×, 15 mL). The combined organic layers were dried over MgSO_4_, filtered, and concentrated *in vacuo*. To the residue, EtOH (5 mL), an aqueous solution of NH_4_Cl at a concentration of 166 mM in water (4.85 mL, 0.64 mmol), and Fe powder (0.22 g, 4.02 mmol) were added, the resulting reaction mixture was stirred at 80 °C for 2.5 h. Next, the organic solvent was evaporated *in vacuo*, water (20 mL) was added, and the solution was extracted with DCM (3×, 15 mL). The combined organic layers were dried over MgSO_4_, filtered, and concentrated in vacuo. Subsequent, the residue was solubilized with DCM (15 mL), and 1-isocyanato-4-nitrobenzene (0.11 g, 0.68 mmol) was added. The resulting reaction mixture was stirred at room temperature for 1 h, after which, DMF was removed on reduced pressure, and the residue was purified using preparative RP-HPLC to yield **30** as a yellow solid (0.03 g, 9% yield). ^1^H NMR (500 MHz, DMSO-*d*_6_) δ 10.2 (br s, 1H), 9.7 (br s, 1H), 8.2 (d, *J* = 9.2 Hz, 2H), 7.7 (d, *J* = 9.2 Hz, 2H), 7.6 (s, 1H), 7.4 (br d, *J* = 8.1 Hz, 1H), 7.3 (t, *J* = 7.8 Hz, 1H), 7.0 (d, *J* = 7.5 Hz, 1H), 3.7 (s, 2H), 2.6 (br s, 4H), 1.8 (br s, 4H). ^13^C NMR (126 MHz, DMSO-*d*_6_) δ 164.3, 151.9, 146.5, 140.5, 139.1, 128.4, 124.8, 122.5, 118.6, 117.3, 117.1, 58.7, 53.0, 22.6. HR-MS (ESI) calculated for: C_18_H_21_N_4_O_3_ [M + H]^+^: 341.1535, found: 341.16151. HPLC purity: 98%

##### 1-(4-Fluoro-3-(morpholinosulfonyl)phenyl)-3-(4-nitrophenyl)urea (**31**)

According to **GP-3** using, 4-fluoro-3-(morpholinosulfonyl)aniline (0.08 g, 0.46 mmol), to afford after purification by flash chromatography (CH_2_Cl_2_/MeOH, 10/0 → 9.5/0.5), and washing the residue with MeOH, **27** as a yellow powder (0.05 g, 23% yield). ^1^H NMR (500 MHz, DMSO-*d*_6_) δ 9.5 (br s, 1H), 9.3 (br s, 1H), 8.2–8.1 (m, 2H), 8.1 (dd, *J* = 6.0, 2.7 Hz, 1H), 7.8–7.7 (m, 3H), 7.5 (t, J = 9.5 Hz, 1H), 3.7–3.6 (m, 4H), 3.1–3.0 (m, 4H). ^13^C NMR (126 MHz, DMSO-*d*_6_) δ 154.9, 152.9, 152.5, 146.5, 141.7, 136.4, 126, 125.6, 123.7, 120.6, 118.7, 118.5, 118.3, 66, 46. ^19^F NMR(470 MHz DMSO-*d*_6_) δ −116.4. HR-MS (ESI) calculated for: C_17_H_18_FN_4_O_6_S [M + H]^+^: 425.0853, found: 425.0913. HPLC purity: 96%.

##### 1-(4-Chloro-3-(morpholinosulfonyl)phenyl)-3-(4-nitrophenyl)urea (**32**)

According to **GP-3** using, 4-chloro-3-(morpholinosulfonyl)aniline (0.1 g, 0.36 mmol), to afford after filtration, and washing (MeOH, CH_2_Cl_2_ and diethyl ether), **28** as yellow solid (0.01 g, 7% yield). ^1^H NMR (500 MHz, DMSO-*d*_6_) δ 9.7–9.4 (m, 2H), 8.2 (d, *J* = 2.4 Hz, 1H), 8.3–8.2 (m, 2H), 7.8–7.7 (m, 3H), 7.7– 7.6 (m, 1H), 3.7–3.6 (m, 4H), 3.2–3.1 (m, 4H). ^13^C NMR (126 MHz, DMSO-*d*_6_) δ 151.96, 145.98, 141.33, 138.69, 134.93, 132.74, 125.12, 123.85, 123.31, 120.89, 117.90, 65.72, 45.74. HR-MS (ESI) calculated for: C_17_H_18_ClN_4_O_6_S [M + H]^+^: 441.0557, found: 441.0623. HPLC purity: 99%.

##### 1-(4-Methyl-3-(morpholinosulfonyl)phenyl)-3-(4-nitrophenyl)urea (**33**)

According to **GP-3** using, 4-methyl-3-(morpholinosulfonyl)aniline (0.1 g, 39 mmol), affording after purification by flash chromatography (CH_2_Cl_2_/MeOH, 10/90 → 95/05), and recrystallization (MeOH, CH_2_Cl_2_, and diethyl ether), **29** as a white solid (0.02 g, 9% yield). ^1^H NMR (500 MHz, DMSO-*d*_6_) δ 9.5 (br s, 1H), 9.28 (br s, 1H), 8.3–8.2 (m, 2H), 8.1 (d, *J* = 2.4 Hz, 1H), 7.8–7.7 (m, 2H), 7.6 (dd, *J* = 8.2, 2.3 Hz, 1H), 7.4 (d, *J* = 8.4 Hz, 1H), 3.7–3.6 (m, 4H), 3.4 (s, 9H), 3.1–3.0 (m, 4H). ^13^C NMR (126 MHz, DMSO-*d*_6_) δ 152.5, 146.7, 141.6, 137.9, 135.2, 134, 131.3, 125.7, 123.5, 119.8, 118.2, 66, 45.8, 20.2. HR-MS (ESI) calculated for: C_18_H_21_N_4_O_6_S [M + H]^+^: 421.1104, found: 421.1174. HPLC purity: 99%.

##### 1-(4-Fluoro-3-(pyrrolidin-1-ylsulfonyl)phenyl)-3-(4-nitrophenyl)urea (**34**)

According to **GP-4** using, 1-((2-fluoro-5-nitrophenyl)sulfonyl)pyrrolidine (0.1 g, 0.42 mmol), to afford after purification by preparative RP-HPLC **30** as an orange solid (0.01 g, 6% yield). ^1^H NMR (500 MHz, DMSO-*d*_6_): δ 10.2–9.8 (m, 1H), 8.8–8.6 (m, 1H), 8.2 (d, *J* = 9.2 Hz, 2H), 7.8 (d, *J* = 2.3 Hz, 1H), 7.7 (d, *J* = 9.2 Hz, 2H), 7.7 (d, *J* = 2.3 Hz, 1H), 7.0 (d, *J* = 9.0 Hz, 1H), 3.5–3.4 (m, 4H), 1.9–1.8 (m, 4H). ^13^C NMR (126 MHz, DMSO-*d*_6_): δ 153.1, 150.7, 146.6, 140.7, 129.3, 127.1, 124.9, 123.4, 117.3, 115.2, 49.8, 24.9. ^19^F NMR (470 MHz DMSO-*d*_6_) δ −73.5. HR-MS (ESI) calculated for: C_17_H_17_FN_4_O_5_S [M + H]^+^: 409.0904, found: 409.0967. HPLC purity: 96%.

##### 1-(2-Fluoro-5-(pyrrolidin-1-ylsulfonyl)phenyl)-3-(4-nitrophenyl)urea (**35**)

According to **GP-4** using, 4-fluoro-3-nitrobenzenesulfonyl chloride (0.1 g, 0.42 mmol), to afford after purification by preparative RP-HPLC **31** as a gray solid (0.01 g, 5% yield). ^1^H NMR (500 MHz, DMSO-*d*_6_): δ 9.6 (br s, 1H), 9.2 (br s, 1H), 8.2 (d, *J* = 2.4 Hz, 1H), 8.2 (d, *J* = 9.2 Hz, 2H), 7.7 (d, *J* = 9.2 Hz, 2H), 7.7 (dd, *J* = 9.2, 2.3 Hz, 1H), 7.3 (d, *J* = 9.2 Hz, 1H), 3.4–3.3 (m, 4H), 2.0–1.9 (m, 4H). ^13^C NMR (126 MHz, DMSO-*d*_6_): δ 152.1, 146.2, 144.9, 141.0, 130.3, 128.4, 125.0, 120.7, 120.0, 117.5, 51.8, 25.1. ^19^F NMR (470 MHz DMSO-*d*_6_) δ −115.9. HR-MS (ESI) calculated for: C_17_H_17_FN_4_O_5_S [M + H]^+^: 409.0904, found: 409.0967. HPLC purity: 98.

##### 1-(2-Chloro-5-(pyrrolidin-1-ylsulfonyl)phenyl)-3-(4-nitrophenyl)urea (**36**)

According to **GP-4** using, 4-chloro-3-nitrobenzenesulfonyl chloride (0.15 g, 0.59 mmol), to afford after purification by preparative RP-HPLC **32** as a yellow solid (0.02 g, 7% yield). ^1^H NMR (500 MHz, DMSO-*d*_6_): δ 10.3–10.1 (m, 1H), 8.9–8.7 (m, 1H), 8.7 (d, *J* = 2.0 Hz, 1H), 8.2 (d, *J* = 9.2 Hz, 2H), 7.8 (d, *J* = 8.4 Hz, 1H), 7.7 (d, *J* = 9.2 Hz, 2H), 7.5 (dd, *J* = 8.4, 2.0 Hz, 1H), 3.2–3.1 (m, 4H), 1.7–1.6 (m, 4H). ^13^C NMR (126 MHz, DMSO-*d*_6_): δ 152.1, 146.0, 142.0, 136.7, 135.9, 130.8, 126.8, 125.7, 122.5, 119.8, 118.0, 118.3, 48.4, 25.2. HR-MS (ESI) calculated for: C_17_H_17_ClN_4_O_5_S [M + H]^+^: 425.0608, found: 425.0686. HPLC purity: 97%.

##### 1-(2-Methyl-5-(pyrrolidin-1-ylsulfonyl)phenyl)-3-(4-nitrophenyl)urea (**37**)

According to **GP-4** using, 4-methyl-3-nitrobenzenesulfonyl chloride (0.15 g, 0.59 mmol), to afford after purification by preparative RP-HPLC **33** as a yellow solid (0.07 g, 28% yield). ^1^H NMR (500 MHz, DMSO-*d*_6_): δ 10.0–9.7 (m, 1H), 8.4 (d, *J* = 1.7 Hz, 1H), 8.2 (d, *J* = 9.2 Hz, 2H), 7.7 (d, *J* = 9.2 Hz, 2H), 7.5 (d, *J* = 7.9 Hz, 1H), 7.4 (dd, *J* = 8.7, 2.0 Hz, 1H), 3.2–3.1 (m, 4H), 2.4 (s, 3H), 1.7–1.6 (m, 4H). ^13^C NMR (126 MHz, DMSO-*d*_6_): δ 151.9, 145.9, 141.0, 137.4, 133.7, 132.5, 130.9, 125.1, 125.0, 121.4, 118.8, 117.8, 117.4, 47.6, 24.5, 17.8. HR-MS (ESI) calculated for: C_18_H_20_N_4_O_5_S [M + H]^+^: 405.1154, found: 405.1219. HPLC purity: 98%.

##### 1-(2,5-Dimethyl-3-(pyrrolidin-1-ylsulfonyl)phenyl)-3-(4-nitrophenyl)urea (**38**)

According to **GP-4** using, 2,5-dimethyl-3-nitrobenzenesulfonyl chloride (0.15 g, 0.60 mmol), to afford after purification by preparative RP-HPLC **34** as a yellow solid (0.09 g, 37% yield). ^1^H NMR (500 MHz, DMSO-*d*_6_): δ 9.9–9.7 (m, 1H), 8.5–8.2 (m, 1H), 8.2 (d, *J* = 9.2 Hz, 2H), 7.8–7.7 (m, 1H), 7.7 (d, *J* = 9.2 Hz, 2H), 7.5–7.4 (m, 1H), 3.2 (m, 4H), 2.4 (s, 3H), 2.4 (s, 3H), 1.9–1.8 (m, 4H). ^13^C NMR (126 MHz, DMSO-*d*_6_): δ 152.4, 146.5, 141.2, 138.7, 137.6, 135.6, 128.3, 126.2, 125.4, 125.0, 117.6, 47.4, 25.2, 20.9, 14.1. HR-MS (ESI) calculated for: C_19_H_22_N_4_O_5_S [M + H]^+^: 419.1311, found: 419.1373. HPLC purity: 100%.

##### 1-(4-Nitrophenyl)-3-(4-(pyrrolidin-1-yl)-3-(pyrrolidin-1-ylsulfonyl)phenyl)urea (**39**)

To a flask containing acetonitrile (1.5 mL), trimethylamine (0.12 g, 1.17 mmol), and pyrrolidine (0.04 g, 0.59 mmol) at room temperature, 2-chloro-5-nitrobenzenesulfonyl chloride (0.15 g, 0.59 mmol) was added. Next the resulting solution was stirred at room temperature for 5 min, after which, the solvent was evaporated. To the residue, EtOH (5.4 mL, 0.1 mmol), an aqueous solution of NH_4_Cl at a concentration of 166 mM in water (2.0 mL, 0.34 mmol), and Fe powder (0.19 g, 3.44 mmol) were added, the resulting reaction mixture was stirred at 80 °C for 2.5 h. Next, the organic solvent was evaporated *in vacuo*, water (20 mL) was added, and the solution was extracted with EtOAc (3×, 20 mL). The combined organic layers were dried over MgSO_4_, filtered, and concentrated *in vacuo*. Subsequent, the residue was solubilized with DMF (2 mL), and 1-isocyanato-4-nitrobenzene (0.14 g, 0.86 mmol) was added. The resulting reaction mixture was stirred at room temperature for 1 h, after which, DMF was removed on reduced pressure, and the residue was purified using preparative RP-HPLC to yield **35** as a yellow solid (0.01 g, 13% yield). ^1^H NMR (500 MHz, DMSO-*d*_6_): δ 9.6–9.3 (m, 1H), 9.3–9.0 (m, 1H), 8.2 (d, *J* = 9.2 Hz, 2H), 8.0 (d, *J* = 2.6 Hz, 1H), 7.7 (d, *J* = 9.2 Hz, 1H), 7.6 (dd, *J* = 8.9, 2.6 Hz, 1H), 7.4 (d, *J* = 8.9 Hz, 1H), 3.3–3.2 (m, 4H), 3.2–3.1 (m, 4H), 1.9–1.8 (m, 4H), 1.8–1.7 (m, 4H). ^13^C NMR (126 MHz, DMSO-*d*_6_): δ 152.0, 146.3, 144.3, 141.0, 134.1, 133.6, 125.1, 123.8, 123.4, 120.8, 117.6, 53.6, 47.7, 25.4, 24.2. HR-MS (ESI) calculated for: C_21_H_25_N_5_O_5_S [M + H]^+^: 460.1576, found: 460.1653. HPLC purity: 99%.

##### 1-(4-Nitrophenyl)-3-(2-(pyrrolidin-1-yl)-5-(pyrrolidin-1-ylsulfonyl)phenyl)urea (**40**)

To a flask containing acetonitrile (1.5 mL), trimethylamine (0.12 g, 1.17 mmol), and pyrrolidine (0.04 g, 0.59 mmol) at room temperature, 4-chloro-3-nitrobenzenesulfonyl chloride (0.15 g, 0.59 mmol) was added. Next the resulting solution was stirred at room temperature for 5 min, after which, the solvent was evaporated. To the residue, EtOH (5.4 mL, 0.1 mmol), an aqueous solution of NH_4_Cl at a concentration of 166 mM in water (2.0 mL, 0.34 mmol), and Fe powder (0.19 g, 3.44 mmol) were added, the resulting reaction mixture was stirred at 80 °C for 2.5 h. Next, the organic solvent was evaporated *in vacuo*, water (20 mL) was added, and the solution was extracted with EtOAc (3×). The combined organic layers were dried over MgSO_4_, filtered, and concentrated *in vacuo*. Subsequently, the residue was solubilized with DMF (2 mL) and 1-isocyanato-4-nitrobenzene (0.14 g, 0.86 mmol) was added. The resulting reaction mixture was stirred at room temperature for 1 h, after which, DMF was removed on reduced pressure and the residue was purified using preparative RP-HPLC to yield **36** as a yellow solid (0.02 g, 9% yield) ^1^H NMR (500 MHz, DMSO-*d*_6_): δ 9.8 (br s, 1H), 8.2 (br s, 1H), 8.2 (d, *J* = 9.2 Hz, 2H), 7.8 (d, *J* = 2.1 Hz, 1H), 7.7 (d, *J* = 9.3 Hz, 2H), 7.4 (dd, *J* = 8.7, 2.3 Hz, 1H), 7.0 (d, *J* = 8.9 Hz, 1H), 3.3 (m, 4H), 3.1–3.0 (m, 4H), 2.0–1.9 (m, 4H), 1.7–1.6 (m, 4H). ^13^C NMR (126 MHz, DMSO-*d*_6_): δ 152.9, 147.4, 146.7, 141.1, 126.0, 125.7, 125.3, 125.2, 124.5, 117.6, 115.8, 50.2, 47.9, 25.0, 24.7. HR-MS (ESI) calculated for: C_21_H_25_N_5_O_5_S [M + H]^+^: 460.1576, found: 460.1652. HPLC purity: 97%.

### Photometric *In Vitro* Assay

Dilution series (1:2) of inhibitors in DMSO covered the concentration range of approximately 200–0.01 μM. After finishing the dilution series, the final volume of compound solution in DMSO per well was 3 μL. For the IspD assay, 30 μL aliquots of a solution containing 100 mM Tris-HCl, pH 7.6, 0.02% NaN_3_, 1 mM MEP and 1 mM CTP were added to microplate wells preloaded with 3 μL of DMSO containing test compounds. The reaction was started by addition of 27 μL aliquots of buffer: 100 mM Tris-HCl, pH 7.6, containing 10 mM MgCl_2_, 60 mM KCl, 10 mM dithiothreitol, 0.02% NaN_3_, 1 mM NADH, 2 mM phosphoenolpyruvate, 2 mM ATP, 1 U mL^–1^ pyruvate kinase, 1 U mL^–1^ lactate dehydrogenase, 1.5 U mL^–1^*Escherichia coli* IspE, 0.01 μM *Pf*IspD. The reaction was monitored photometrically (340 nm) at room temperature for 30–60 min on a plate reader (Spectramax M2, Molecular Devices, Biberach an der Riss, Germany). Initial rates were estimated using Softmax Pro 6.1 software (Molecular Devices, Biberach an der Riss, Germany). IC_50_ values were determined with a nonlinear regression method using the program Dynafit.^21^

### Whole-Cell Assay

*Pf*NF54 wild type parasites cultured in RPMI 1640 medium supplemented with 25 mM HEPES, 24 mM sodium bicarbonate (pH 7.3), 0.36 mM hypoxanthine, 100 μg/mL neomycin and 0.5% Albumax II were used to test for compound activity on parasite multiplication using a [^3^*H*]-hypoxanthine incorporation assay.^22^ Compounds were dissolved in DMSO (10 mM), diluted in hypoxanthine-free culture medium and titrated in duplicate over a 64-fold range (6 step 2-fold dilutions) in 96 well plates. 100 μL Asexual parasite culture (prepared in hypoxanthine-free medium) were added to each well and mixed with the compound to obtain a final hematocrit of 1.25% and a final parasitemia of 0.3%. After incubation for 48 h, 0.25 μCi of [^3^*H*]-hypoxanthine was added per well and plates were incubated for an additional 24 h. Parasites were then harvested onto glass-fiber filters using a Microbeta FilterMate cell harvester (Perkin-Elmer, Waltham), and radioactivity was counted using a MicroBeta2 liquid scintillation counter (Perkin-Elmer, Waltham). The results were recorded and expressed as a percentage of the untreated controls. Fifty percent inhibitory concentrations (EC_50_) were estimated by linear interpolation.^23^

### *P. falciparum* Culture

*P. falciparum* strain 3D7 (wild-type, WT) was sourced from MR4 as part of the BEI Resources Repository, NIAID, NIH (www.mr4.org). *P. falciparum* strains resistant to 1*R*,3*S*-MMV008138 (R2 and R3), containing mutations in *Pf*IspD, were generated in strain 3D7. Parasites were cultured in a 2% suspension of human erythrocytes in RPMI 1640 (Sigma) medium supplemented with 27 mM sodium bicarbonate, 11 mM glucose, 5 mM HEPES, 1 mM sodium pyruvate, 0.37 mM hypoxanthine, 0.01 mM thymidine, 10 μg/mL gentamicin, and 0.5% Albumax (Gibco). Cultures were maintained at 37 °C, 5% O_2_/5% CO_2_/90% N_2_ atmosphere.

### Drug Sensitivity Assays

Asynchronous cultures of *P. falciparum*, wild type (3D7) and 1*R*,3*S*-MMV008138-resistant strains (R2 and R3) were diluted to 0.5% parasitemia and 2% hematocrit. The parasites were then exposed to varying concentrations (0–240 μM) of *Pf*IspD-targeting compound **10** for 72 h in a 96-well plate with 100 μL culture volumes per well. After 72 h, parasite growth was quantified using PicoGreen assay (Invitrogen) to measure the DNA content as previously described.^10^ Fluorescence measurement was taken using a CLARIOstar Plus microplate reader (BMG Labtech) at 485 nm excitation/528 nm emission. To determine the effectiveness of **10**, half maximal inhibitory concentrations (IC_50_) values were calculated using nonlinear regression analysis in GraphPad Prism software. The reported IC_50_ value represent the mean ± standard error of the mean (SEM) from at least three independent replicates.

### IDP Rescue Assay

For studying the reversal of growth inhibition, *Pf*3D7 (WT), and 1*R*,3*S*-MMV008138 resistant parasites (R2 and R3) were grown in the presence of varying concentrations of **10**, and the presence or absence of 200 μM isopentenyl pyrophosphate (IDP; Echelon Biosciences) for 72 h. The parasite growth was quantified by measuring DNA content using PicoGreen assay (Invitrogen). IC_50_ values were calculated by nonlinear regression analysis using GraphPad Prism software and reflect the mean ± standard error of the mean (SEM) from at least three independent experiments. Statistical comparison of the values was done via one-way ANOVA.

### LC-MS Based *In Vitro* Assay

Dilution series (1:2) of inhibitors in DMSO covered the concentration range of approximately 200–0.01 μM. After finishing the dilution series, the final volume of compound solution in DMSO per well was 2.0 μL. During the assay, the following buffer was used: 100 mM Tris-HCl pH 7.6, 1 mM DTT. To start the assay, aliquots of buffer (49 μL) containing: 306.1 μM CTP, 2.0 mM MgCl_2_ and 0.1 μM *Pf*IspD, were added to a 96-well plate (Nunc V). Next 2 μL of the inhibitor dilutions (in DMSO) are added and the plate is allowed to incubate at 37 °C for 10 min. Then another 49 μL of buffer containing 306.1 μM MEP was added to start the reaction. The plates were incubated at 37 °C for 40 min, after which, the protein was denaturated by heating up the plate to 95 °C for 5 min. The plate was then centrifuged at 4000 rpm at 4 °C for 5 min to precipitate all solids present in the solution. To another 96-well plate, 190 μL of ice cold 3:1:1 ACN, isopropanol, water mixture was added. Thereafter, 10 μL of each of the supernatants from the assay plate were added. The plate was centrifuged again at 4000 rpm at 4 °C for 5 min, after which, 50 μL of the supernatant was transferred to a plate capable to measured in the MS and covered with a silicon cover. LC-MS conditions and data analysis methods we used were described above.

### Determination of Enzyme Kinetics

A volume of 80 μL Buffer A containing 100 mM Tris-HCl pH 7.6, 1 mM DTT, 1 mM MgCl_2_, 50 nM *Pf*IspD were added to well A1 96-well plate while 50 μL were added to the rest of the wells. A volume of 20 μL of 10 mM CTP was added to the first well, then a serial dilution was conducted by moving 50 μL. To start the reaction, we then added on buffer A 50 μL of Buffer B containing 100 mM Tris-HCl pH 7.6, 1 mM DTT, and 1 mM MEP. The assay plate was incubated at 37 °C for 40 min, after which, the protein was denaturated by heating up the plate to 95 °C for 5 min. The plate was then centrifuged at 4000 rpm at 4 °C for 5 min to precipitate the protein. To another 96-well plate, 190 μL of ice cold 3:1:1 ACN, isopropanol, water mixture was added containing 100 nM 4-methyl-1-oxo-1-(*p*-tolylamino)pentane-2-sulfonic acid, adenylyl-imidodiphosphate and adenosine-5′-[(α,β)-methyleno]triphosphate as internal standard.^15^ Thereafter, 10 μL of each of the supernatants from the assay plate were added to the plate containing the mixture with our internal standard. The plate was centrifuged again at 4000 rpm at 4 °C for 5 min, after which, 50 μL of the supernatant was transferred to an LC-MS plate and closed with a silicon cover. LC-MS conditions and data analysis methods we used were described above. The peak area for each conditions were used to calculate the Michaelis–Menten kinetic parameters using Graphpad Prism v 9. Measurements were performed in duplicates, repeated at least two times from two to three independent experiments.

### Determination of Mode of Inhibition of **10**

Dilution series (1:2) of inhibitors in DMSO covered the concentration range of approximately 200–0.01 μM. After finishing the dilution series, the final volume of compound solution in DMSO per well was 2.0 μL. During the assay, the following buffer was used: 100 mM Tris-HCl pH 7.6, 1 mM DTT. To study the inhibition mode against CTP, aliquots of buffer (49 μL) containing: 0, 37.5, 75, 125, 250, 500 μM CTP, 2.0 mM MgCl_2_ and 0.1 μM PfIspD, were added to a 96-well plate (Nunc V). Next 2 μL of the inhibitor dilutions (in DMSO) are added and the assay plate was incubated at 37 °C for 10 min. Then another 49 μL of buffer containing 500 μM MEP was added to start the reaction. To study the Mode of inhibition toward MEP, similar steps were followed as in case of CTP with using 0, 37.5, 75, 125, 250, 500 μM MEP and 500 μM CTP The assay plate was incubated at 37 °C for 40 min, after which, the protein was denaturated by heating up the plate to 95 °C for 5 min. The assay plate was then centrifuged at 4000 rpm at 4 °C for 5 min to precipitate the protein. To another 96-well plate, 190 μL of ice cold 3:1:1 ACN, isopropanol, water mixture was added containing 100 nM 4-methyl-1-oxo-1-(*p*-tolylamino)pentane-2-sulfonic acid as internal standard.^15^ Thereafter, 10 μL of each of the supernatants from the assay plate were added to the plate containing the mixture with our internal standard. The plate was centrifuged again at 4000 rpm at 4 °C for 5 min, after which, 50 μL of the supernatant was transferred to an LC-MS plate and closed with a silicon cover. LC-MS conditions and data analysis methods we used were described above.

### Metabolic Stability in Liver S9 Fractions

For the evaluation of combined phase I and phase II metabolic stability, the compound (1 μM) was incubated with 1 mg/mL pooled mouse liver S9 fraction (Xenotech, Kansas City) or human liver S9 fraction (Corning, New York, USA), 2 mM NADPH, 1 mM UDPGA, 10 mM MgCl_2_, 5 mM GSH and 0.1 mM PAPS at 37 °C for 240 min. The metabolic stability of testosterone, verapamil and ketoconazole were determined in parallel to confirm the enzymatic activity of mouse S9 fractions, for human S9 testosterone, diclofenac and propranolol were used. The incubation was stopped after defined time points by precipitation of aliquots of S9 enzymes with 2 volumes of cold acetonitrile containing internal standard (150 nM diphenhydramine). Samples were stored on ice until the end of the incubation and precipitated protein was removed by centrifugation (4 °C, 15 min, 4000*g*). Concentration of the remaining test compound at the different time points was analyzed by HPLC-MS/MS (TSQ Quantum Access MAX, Thermo Fisher, Dreieich, Germany) and used to determine half-life (*t*_1/2_).

### Stability in Mouse and Human Plasma

To determine stability in mouse plasma, the compound (1 μM) was incubated with pooled CD-1 mouse or human plasma (Neo Biotech, Nanterre, France). Samples were taken at defined time points by mixing aliquots with 4 volumes of acetonitrile containing internal standard (125 nM diphenhydramine). Samples were stored on ice until the end of the incubation and precipitated protein was removed by centrifugation (4 °C, 15 min, 4000*g*, 2 centrifugation steps). Concentration of the remaining test compound at the different time points was analyzed by HPLC-MS/MS (TSQ Quantum Access MAX, Thermo Fisher, Dreieich, Germany). The plasma stability of procain, propantheline and diltiazem were determined in parallel to confirm the enzymatic activity.

Plasma Protein Binding Plasma protein binding was determined using the Rapid Equilibrium Dialysis (RED) system (Thermo Fisher Scientific, Waltham MA, USA). Compounds were diluted to 10 μM in 50% murine (CD-1) or human plasma (Neo Biotech, Nanterre, France) in PBS pH 7.4 and added to the respective chamber according to the manufacturer’s protocol, followed by addition of PBS pH 7.4 to the opposite chamber. Samples were taken immediately after addition to the plate as well as after 2, 4 and 5 h by mixing 10 μL with 80 μL ice-cold acetonitrile containing 12.5 nM diphenhydramine as internal standard, followed by addition of 10 μL plasma to samples taken from PBS and vice versa. Samples were stored on ice until the end of the incubation and precipitated protein was removed by centrifugation (15 min, 4 °C, 4,000 g, 2 centrifugation steps). Concentration of the remaining test compound at the different time points was analyzed by HPLC-MS/MS (Vanquish Flex coupled to a TSQ Altis Plus, Thermo Fisher, Dreieich, Germany). The amount of compound bound to protein was calculated using the equation PPB [%] = 100 – 100 x (amount in buffer chamber/amount in plasma chamber).

### Pharmacokinetic (PK) Studies

For pharmacokinetic experiments, outbred male CD-1 mice (Charles River, Germany), 4 weeks old, were used. The animal studies were conducted in accordance with the recommendations of the European Community (Directive 2010/63/EU, first January 2013). All animal procedures were performed in strict accordance with the German regulations of the Society for Laboratory Animal Science (GV-SOLAS) and the European Health Law of the Federation of Laboratory Animal Science Associations (FELASA). Animals were excluded from further analysis if sacrifice was necessary according to the humane end points established by the ethical board. All experiments were approved by the ethical board of the Niedersächsisches Landesamt für Verbraucherschutz and Lebensmittelsicherheit, Oldenburg, Germany. Compounds **10** and **28** were administered at 1 mg/kg intravenously in a cassette format (*n* = 2). At the time points 0.25, 0.5, 1, and 3 post administration, up to 25 μL of blood were collected from the lateral tail vein. At 5 h post administration, mice were euthanized to collect blood from the heart as well as to remove spleen and liver aseptically. Whole blood was collected into Eppendorf tubes coated with 0.5 M EDTA and immediately spun down at 15,870*g* for 10 min at 4 °C. Then, plasma was transferred into a new Eppendorf tube spleen and liver were homogenized using a Polytron tissue homogenizer. Spleen, liver, and plasma samples were stored at −80 °C until analysis. First, a calibration curve was prepared by spiking different concentrations of **10** and **28** into mouse plasma, homogenized spleen or homogenized liver from CD-1 mice. Glipizide was used as an internal standard. In addition, quality control samples (QCs) were prepared for **10** and **26** in the same matrices. For **10** and **28** the same extraction procedure was used: 7.5 μL of a plasma sample (calibration samples, QCs or PK samples) was extracted with 22.5 μL of acetonitrile containing 12.5 ng/mL of glipizide as an internal standard for 5 min at 2000 rpm on an Eppendorf MixMate vortex mixer. Then samples were spun down at 13.000 rpm for 10 min. Supernatants were transferred to standard HPLC-glass vials. For liver and spleen, 20 μL of a sample (calibration samples, QCs or PK samples) were extracted with 10 μL water containing 10% formic acid, and 22.5 μL acetonitrile with 12.5 ng/mL of glipizide as internal standard. Samples were extracted for 5 min at 800 rpm on an Eppendorf MixMate vortex mixer and spun down for 5 min at 4000 rpm. Peaks of PK samples were quantified using the calibration curve. The accuracy of the calibration curve was determined using QCs independently prepared on different days (Table S4). PK parameters were determined using a noncompartmental analysis with PKSolver.^24^

### Cytotoxicity Study

An MTT-based assay was employed to evaluate the viability of HepG2 after challenge with selected inhibitors and performed as described previously.^25^

## Supporting Information Available

Activities of selected compounds against auxiliary enzymes; calibration curve for CDP-ME; kinetic parameters concerning MEP and CTP; LC-MS chromatograms of MEP, CDP-ME, and the internal standard; Q1 and Q3 masses for glipizide, **10** and **28**; supplementary reaction schemes; activities of representative compounds against different bacterial pathogens; ^1^H, ^13^C NMR spectra, and HPLC traces of all final compounds (PDF)
Molecular formula strings, activity data against *Pf*IspD and whole-cell activity against *Pf*NF54 (CSV)

## Abbreviations Used

AMR: antimicrobial resistance
AUC0-*t*: area under the concentration–time curve from time zero to time *t*
CDP-ME: 4-diphosphocytidyl-2-*C*-methylerythritol
Cl_obs: clearance (based on observed last time point with measurable concentration)
CTP: cytidine triphosphate
CuAAC: copper-catalyzed azide-alkyne cycloaddition
DDA: data-dependent acquisition
DIA: data-independent acquisition
DCM: dichloromethane
DMADP: dimethylallyl diphosphate
DMF: *N*,*N*-dimethylformamide
DMSO: dimethyl sulfoxide
Eq: equivalents
ESI: electron spray ionization
FA: formic acid
EC_50_: fifty percent inhibitory concentrations
HESI: heated electrospray ionization
HTS: high throughput screening
HPLC: high pressure liquid chromatography
IDP: isopentenyl diphosphate
IV: intravenous
Km: Michaelis constant
LCMS: liquid chromatography-mass spectrometry
MEP: 2-*C*-methylerythritol-*D*-erythritol-4-phosphate
MRT: mean residence time
MWD: multiple wave detector
n.a.: no activity
ND: not detected
NMR: nuclear magnetic resonance
*Pf*: *Plasmodium falciparum*
PPi: inorganic diphosphate
PK: pharmacokinetic
SAR: structure–activity relationship
SD: standard deviation
Vz_obs: volume of distribution associated with the terminal phase
