# Supplementary material for: Targeting Plasmodium falciparum IspD in the Methyl-d-erythritol Phosphate Pathway: Urea-Based Compounds with Nanomolar Potency on Target and Low-Micromolar Whole-Cell Activity
Source: J Med Chem. 2024 Sep 20;67(19):17070–86. doi: 10.1021/acs.jmedchem.4c00212 (PMC11472328; doi:10.1021/acs.jmedchem.4c00212)

Targeting *Plasmodium falciparum* IspD in the Methyl-*D*-Erythritol Phosphate Pathway: Urea-Based Compounds with Nanomolar Potency on Target and Low-Micromolar Whole-cell Activity

Daan Willocx,[†,¶,‡] Lorenzo Bizzarri,[ψ,#,¶] Alaa Alhayek,[ψ,†] Deepika Kannan,[Σ] Patricia Bravo,[§,ǁ] Boris Illarionov,[⊥] Katharina Rox,[ʃ,∂] Jonas Lohse,[#] Markus Fischer,[⊥] Andreas M. Kany,[†] Hannes Hahne,[#] Matthias Rottmann,[§,ǁ] Matthias Witschel[¦], Audrey Odom John,[Σ¥] Mostafa M. Hamed,[†] Eleonora Diamanti,[†] Anna K. H. Hirsch[*,†,¶]

[†]Helmholtz Institute for Pharmaceutical Research (HIPS)-Helmholtz Centre for Infection Research (HZI), Campus E8.1, 66123 Saarbrücken, Germany

[¶]Saarland University, Department of Pharmacy, Campus E8.1, 66123 Saarbrücken, Germany

[#]OmicScouts GmbH, Lise-Meitner-Straße 30, 85354 Freising, Germany

[Σ]Department of Pediatrics, Children's Hospital of Philadelphia, Philadelphia, PA 19104, USA

[§]Swiss Tropical and Public Health Institute, Kreuzstrasse 2, 4123 Allschwil, Switzerland

[ʃ]Helmholtz Centre for Infection Research (HZI), Department of Chemical Biology, Inhoffenstraße 7, 38124 Braunschweig, Germany

[∂]German Center for Infection Research (DZIF), Partner site Hannover-Braunschweig, Inhoffenstraße 7, 38124 Braunschweig, Germany

[ǁ]Universität Basel, Petersplatz 1, 4003 Basel, Switzerland

[⊥]Hamburg School of Food Science, University of Hamburg, Grindelallee 117, 20146 Hamburg, Germany

[¦]BASF-SE, Carl-Bosch-Strasse 38, 67056 Ludwigshafen, Germany

[¥]Perelman School of Medicine, University of Pennsylvania, Philadelphia, PA 19104, USA

Corresponding Author: Anna.hirsch@helmholtz-hips.de

Table of Contents

S1. Activities of selected compounds against auxiliary enzymes S2

S2. Calibration curve for CDP-ME S3

S3. Determination kinetic parameters concerning CTP. S4

S4. Determination kinetic parameters concerning MEP. S5

S5. LC-MS chromatograms of MEP, 4-methyl-1-oxo-1-(p-tolylamino)pentane-2-sulfonic acid and CDP-ME respectively S6

S6. Q1 and Q3 masses for glipizide, **10** and **28** S7

S7. Supplementary reaction schemes S8

S8. Activities of representative compounds against different bacterial pathogens S10

S9. ^{1}H NMR and ^{13}C NMR spectra as well as LC-MS and purity traces of Compounds **4**–**40** S11

S1. Activities of selected compounds against auxiliary enzymes

Table S1. Activities of selected compounds against auxiliary enzymes

#	Structure, R=	*Pf*IspD (IC_{50} nM)[a]	EcIspE (IC_{50} µM)[a]	PK-LDH (IC_{50} µM)[a]
**7**	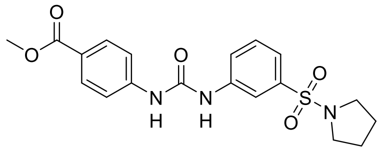	170 ± 20	>500	242 ± 72
**9**	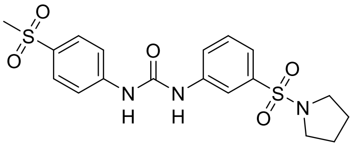	330 ± 40	>500	>500
**11**	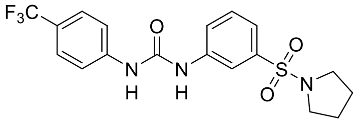	91 ± 19	73 ± 15	73±15
**26**	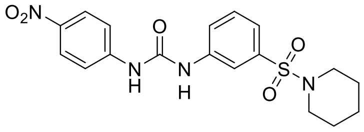	225 ± 20	49 ± 7	>500
**27**	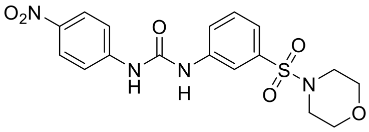	600 ± 110	>500	104 ± 19
**31**	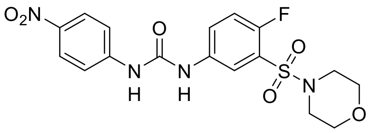	400 ± 100	75 ± 11	>500

[a]Assays were performed in replicate as independent experiments (*n* = 2); values are shown as mean ± SD.

S2. Calibration curve for CDP-ME

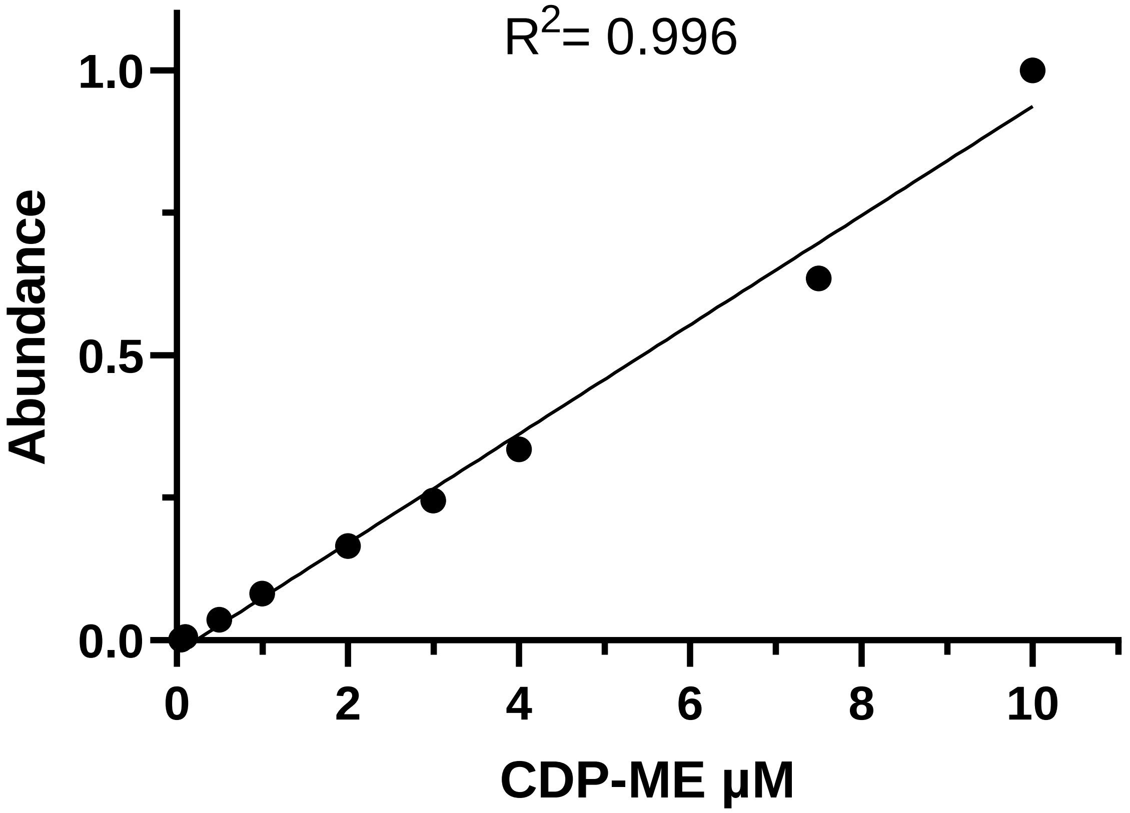

Figure S1. Calibration curve for CDP-ME detection. Concentrations ranging from 50 nM to 10 µM. The calculated area under the curve was converted to values ranging for 0 to 1.

S3. Determination kinetic parameters concerning CTP.

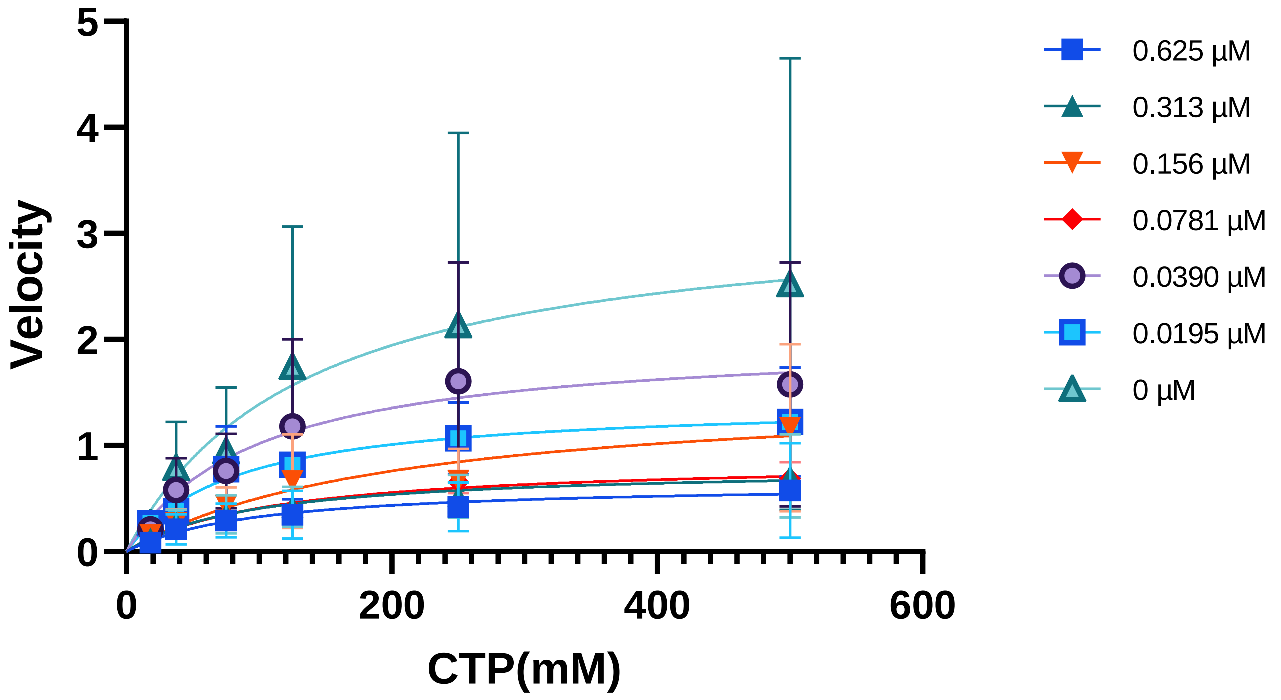

Figure S2. Kinetic parameters were determined concerning the CTP substrate. Each data point reflects the mean ± SD obtained from a minimum of two-independent experiments.

Table S2. Kinetic parameters at different concentrations of compound 10 at varying CTP concentrations.

Compound 10 (µM)	0.625	0.313	0.156	0.0781	0.0390	0.0195	0.000
V_{max}	0.6496	0.8006	1.537	0.8674	2.021	1.418	3.253
K_m	97.89	97.23	204.4	111.7	98.52	80.79	134.4

S4. Determination kinetic parameters concerning MEP.

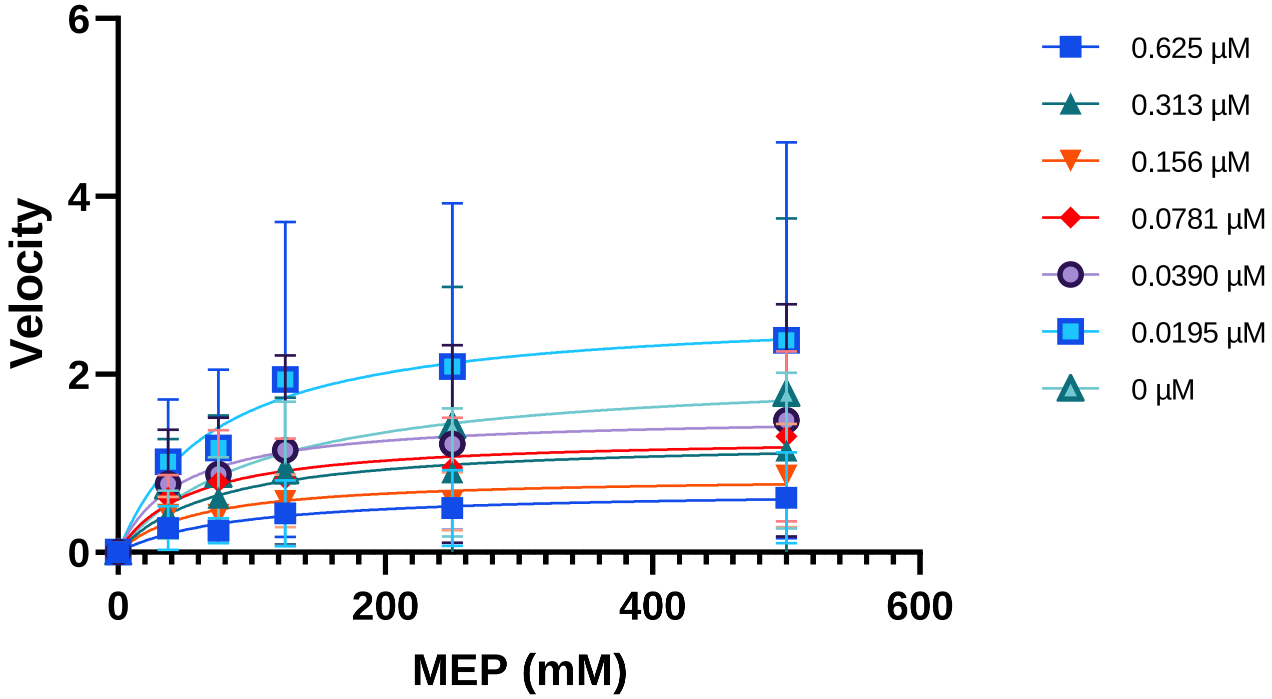

Figure S3. Kinetic parameters were determined concerning the CTP substrate. Each data point reflects the mean ± SD obtained from a minimum of two-independent experiments.

Table S3. Kinetic parameters at different concentrations of compound 10 at varying MEP concentrations

Compound 10 (µM)	0.625	0.313	0.156	0.0781	0.0390	0.0195	0.000
V_{max}	0.7037	1.277	0.8556	1.305	1.541	2.734	2.064
K_m	90.58	74.61	59.79	53.57	46.05	71.50	106.2

S5. LC-MS chromatograms of MEP, 4-methyl-1-oxo-1-(p-tolylamino)pentane-2-sulfonic acid and CDP-ME respectively

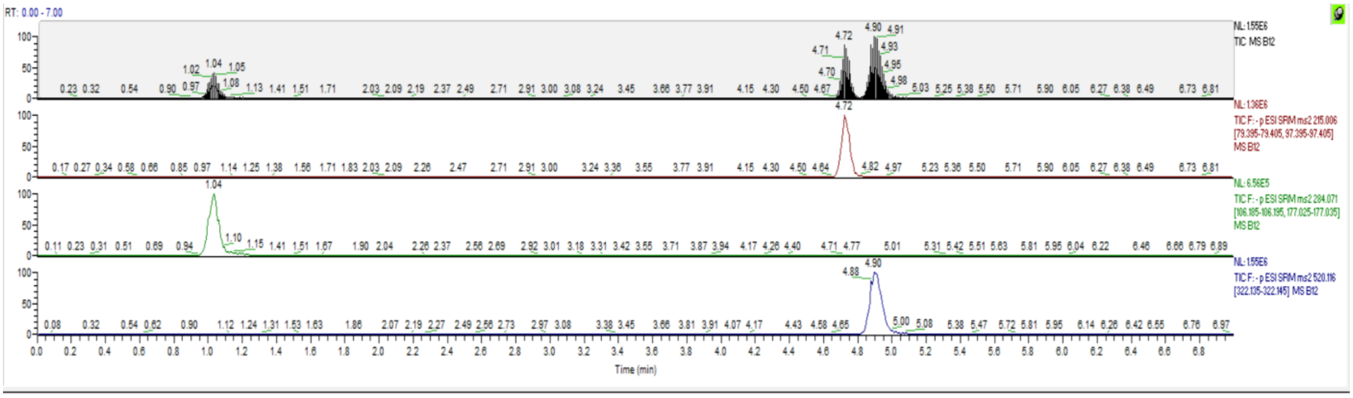

Figure S4. LC-MS chromatogram of MEP (second, red), 4-methyl-1-oxo-1-(p-tolylamino)pentane-2-sulfonic acid (third, green) and CDP-ME (fourth, blue) respectively.

S6. Q1 and Q3 masses for glipizide, 10 and 28

Table S4. Q1 and Q3 masses for glipizide, **10** and **28**

ID	Q1 Mass [Da]	Q3 Mass [Da]	time [msec]	CE [volts]	CXP [volts]	DP [volts]
**10**	371.022	355.0	30	15	20	101
	371.022	253.0	30	25	28	101
**10**	392.998	23.2	30	69	10	96
		353.9	30	13	20	96
		252.2	30	19	22	96
**28**	395.952	136.9	30	-16	-11	-40
		231.9	30	-34	-21	-40
**glipizide**	443.936	319.100	150	-26	-21	-66
**glipizide**	443.936	170.100	150	-40	-7	-66

S7. Supplementary reaction schemes

Scheme S1. Synthetic route for the synthesis of 29

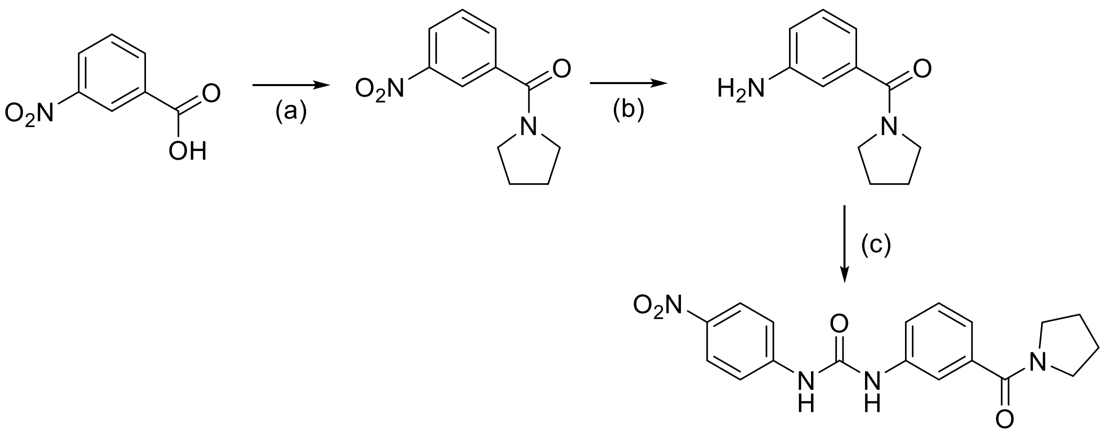

29

[a]Reagents and conditions: (a) pyrrolidine, trimethylamine, propanephosphonic acid anhydride, room temperature, 2 h; (b) NH_4Cl (166 mM in water), Fe powder, 80 °C, 2,5 h; (c) 1-isocyanato-4-nitrobenzene, room temperature, 1 h, 9% yield over all steps.

Scheme S2. Synthetic route for the synthesis of 30

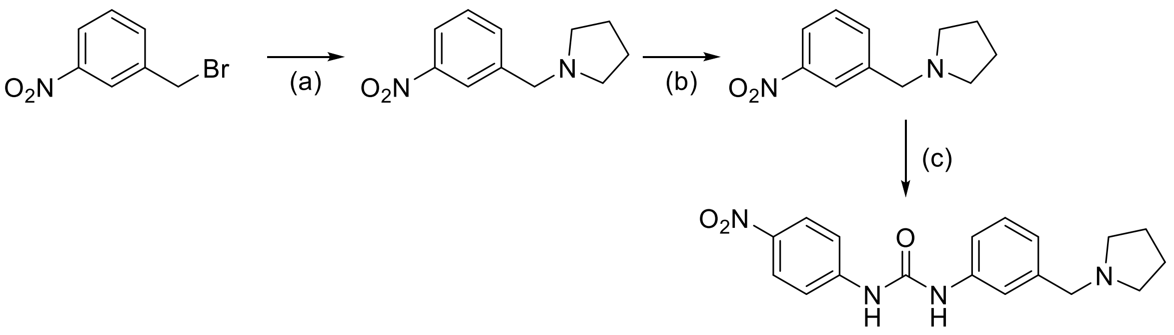

30

[a]Reagents and conditions: (a) pyrrolidine, trimethylamine, room temperature, 2 h; (b) NH_4Cl (166 mM in water), Fe powder, 80 °C, 2,5 h; (c) 1-isocyanato-4-nitrobenzene, room temperature, 1 h, 9% yield over all steps.

Scheme S3. General reaction scheme for the synthesis of compounds 31–33[a].

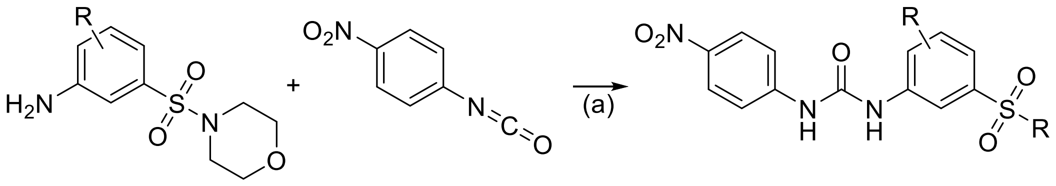

[a]Reagents and reactions conditions: 1-isocyanato-4-nitrobenzene, DMF, room temperature, overnight. 7–23% yield.

S8. Activities of representative compounds against different bacterial pathogens

Table S5 Activities of representative compounds against different bacterial pathogens

#	5	8	10	28
**Inhibition of *E. coli K12* growth (at 100 μM)**	45 ± 6%	14 ± 5%	46 ± 4%	No inhibition
**Inhibition of *E. coli acrB* growth (at 100 μM)**	Full inhibition	71 ± 6%	Full inhibition	No inhibition
**MIC 90 (growth inhibition) of *Mtb H37Rv mCherry 10* strain**	n.a	n.d	64 μM	n.d

[a]Assays were performed in replicate as independent experiments ($n \geq 2$); values are shown as mean ± SD. values are shown as mean ± SD. n.a. indicates the absence of activity

S9. ^{1}H NMR and ^{13}C NMR spectra as well as LC-MS and purity traces of Compounds **4–40**

4

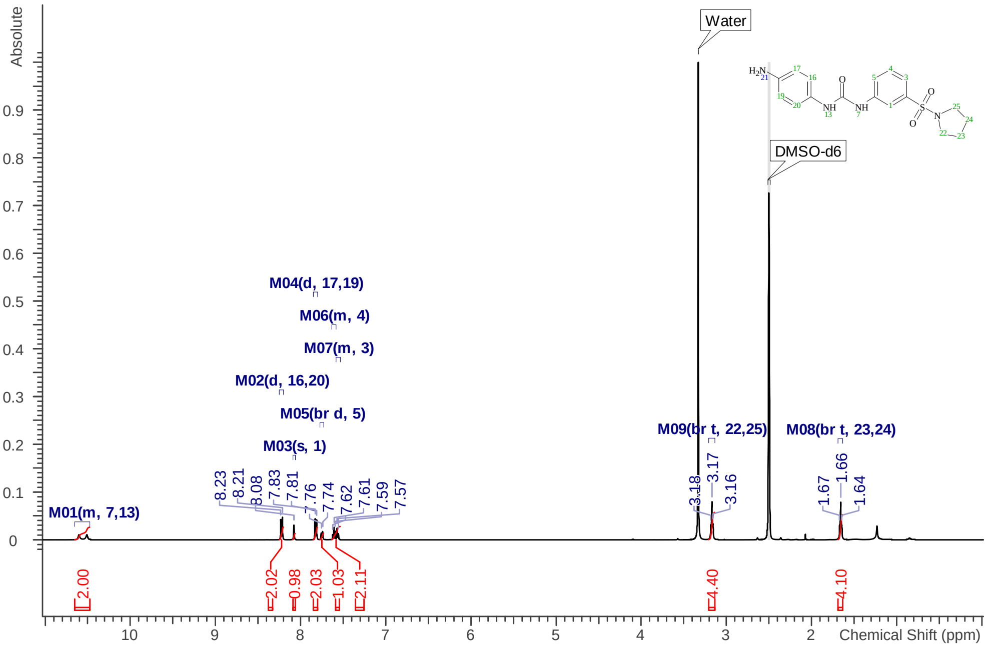

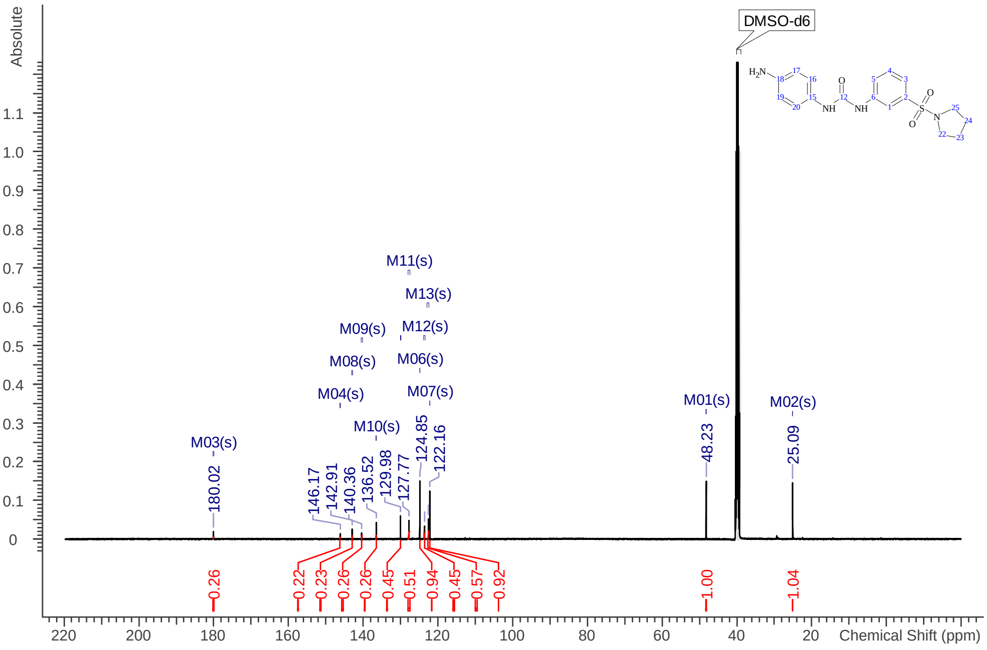

5

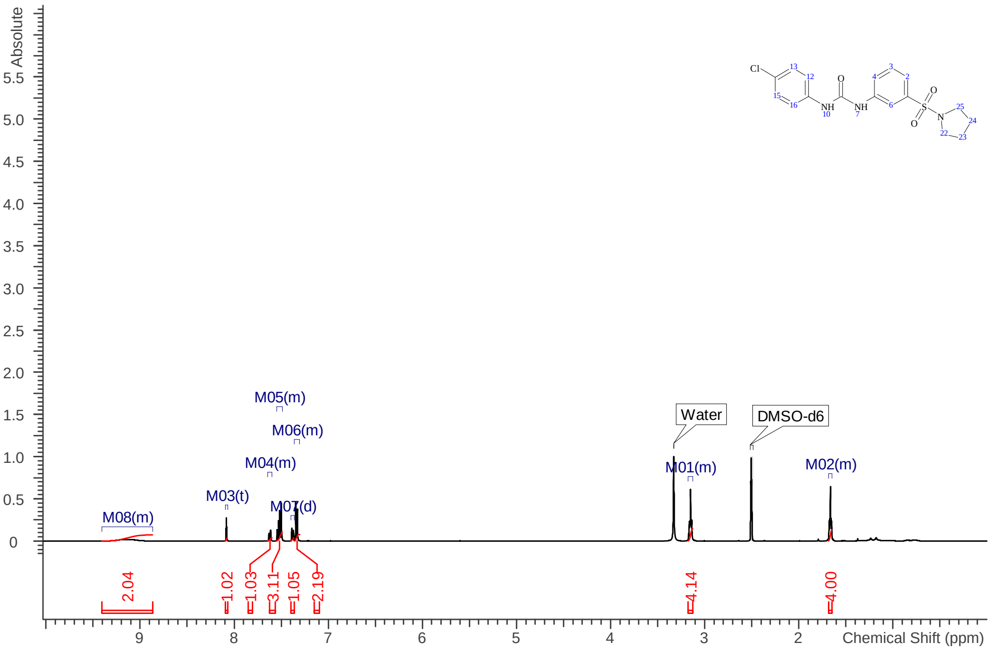

Absolute
Water
DMSO-d6
M05(m)
M06(m)
M04(m)
M03(t)
M07(d)
M08(m)
M01(m)
M02(m)
2.04
1.02
1.03
3.11
1.05
2.19
4.14
4.00
Chemical Shift (ppm)

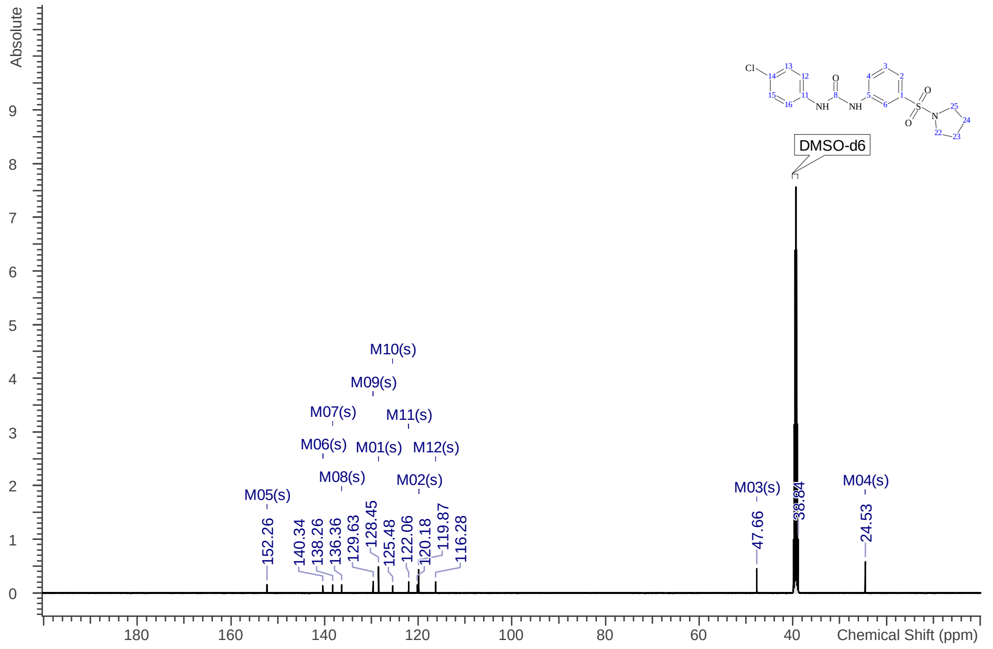

Absolute
DMSO-d6
M10(s)
M09(s)
M07(s)
M11(s)
M06(s)
M01(s)
M12(s)
M08(s)
M02(s)
M05(s)
M03(s)
M04(s)
152.26
140.34
138.26
136.36
129.63
128.45
125.48
122.06
120.18
119.87
116.28
47.66
24.53
Chemical Shift (ppm)

6

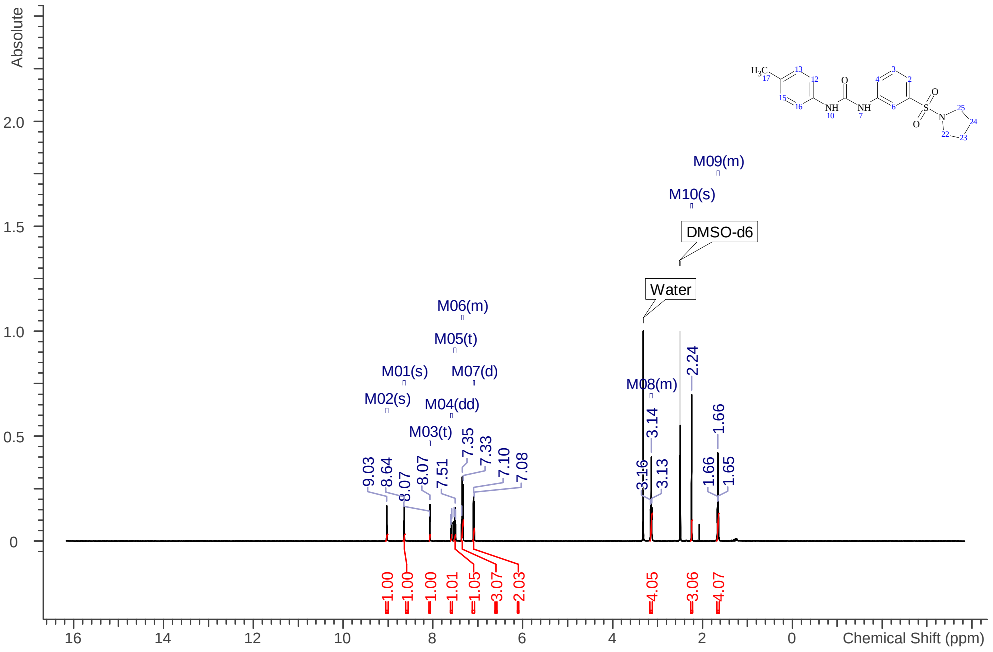

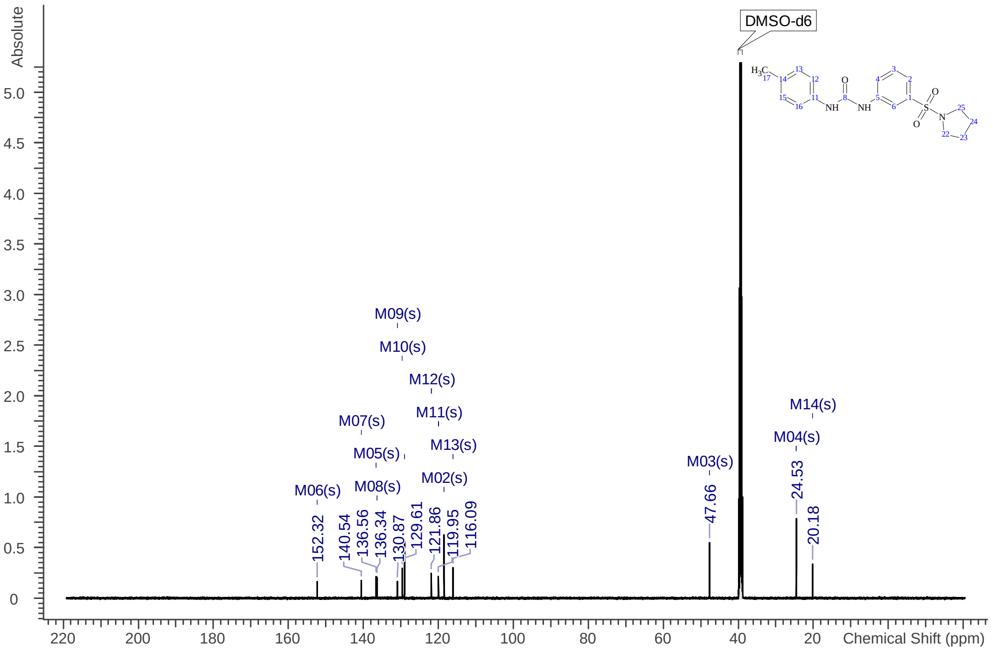

7

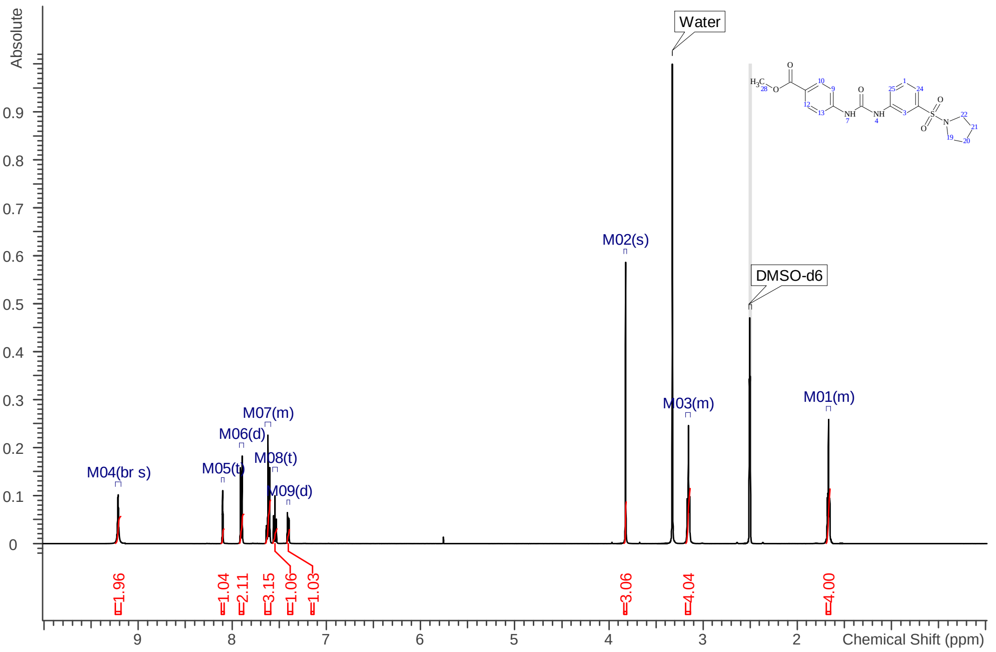

Absolute
Water
DMSO-d6
M01(m)
M02(s)
M03(m)
M04(br s)
M05(t)
M06(d)
M07(m)
M08(t)
M09(d)
1.96
1.04
2.11
3.15
1.06
1.03
3.06
4.04
4.00
Chemical Shift (ppm)

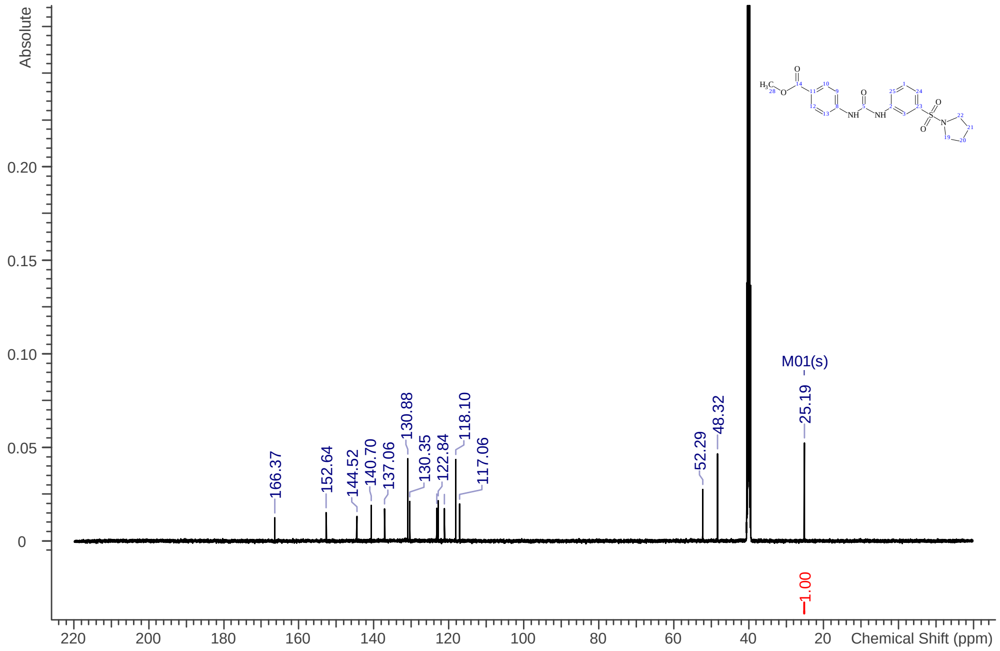

Absolute
M01(s)
166.37
152.64
144.52
140.70
137.06
130.88
130.35
122.84
118.10
117.06
52.29
48.32
25.19
1.00
Chemical Shift (ppm)

8

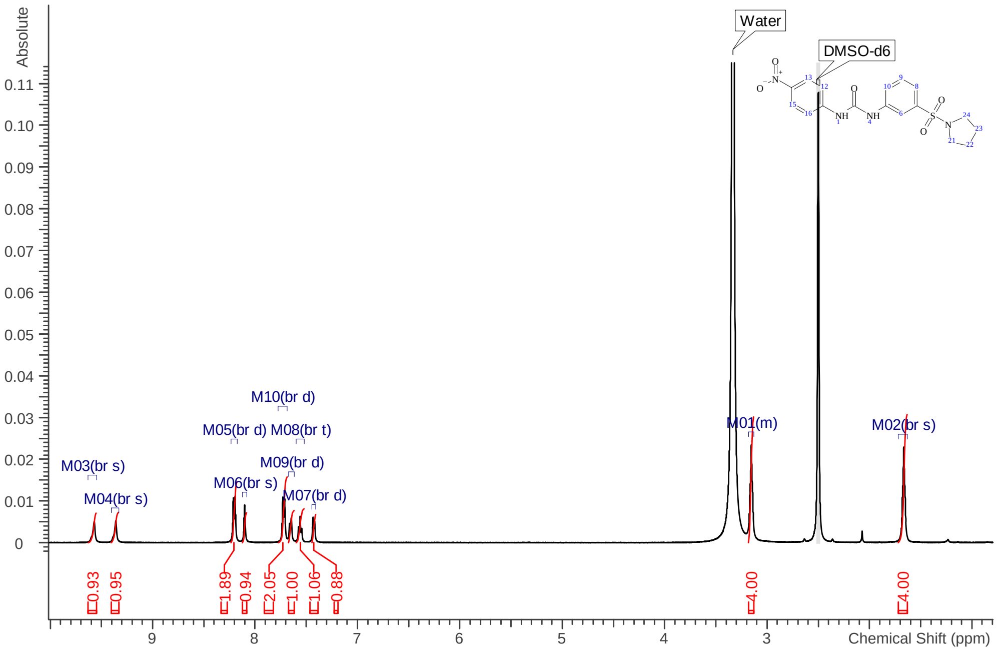

Absolute
Water
DMSO-d6
M10(br d)
M05(br d) M08(br t)
M03(br s)
M09(br d)
M06(br s)
M04(br s)
M07(br d)
M01(m)
M02(br s)
0.93
0.95
1.89
0.94
2.05
1.00
1.06
0.88
4.00
4.00
Chemical Shift (ppm)

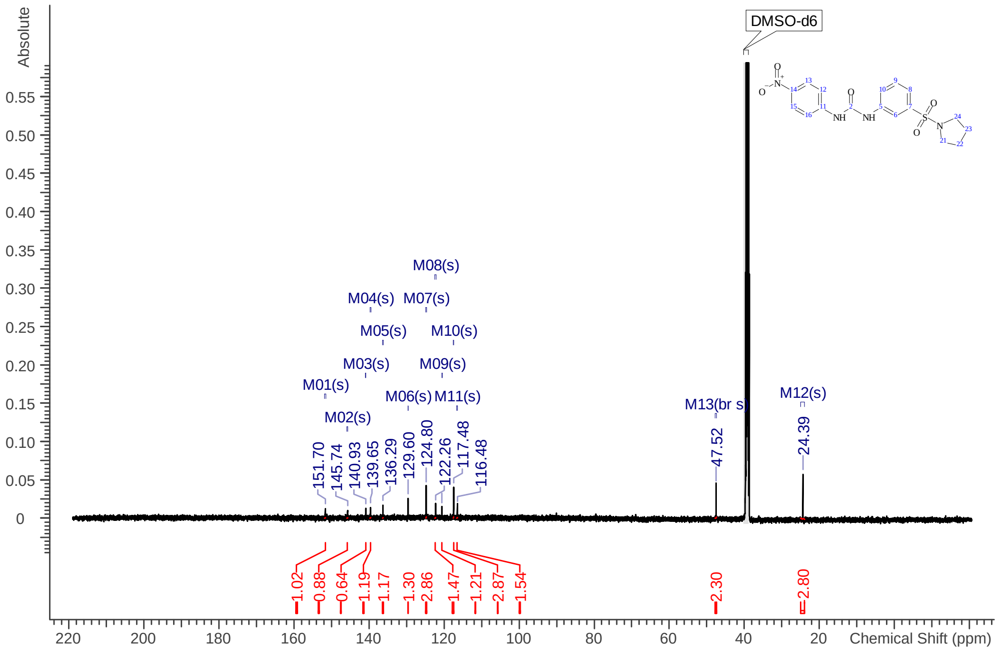

Absolute
DMSO-d6
M08(s)
M04(s) M07(s)
M05(s) M10(s)
M03(s) M09(s)
M01(s)
M06(s) M11(s)
M02(s)
M13(br s)
M12(s)
151.70
145.74
140.93
139.65
136.29
129.60
124.80
122.26
117.48
116.48
47.52
24.39
1.02
0.88
0.64
1.19
1.17
1.30
2.86
1.47
1.21
2.87
1.54
2.30
2.80
Chemical Shift (ppm)

9

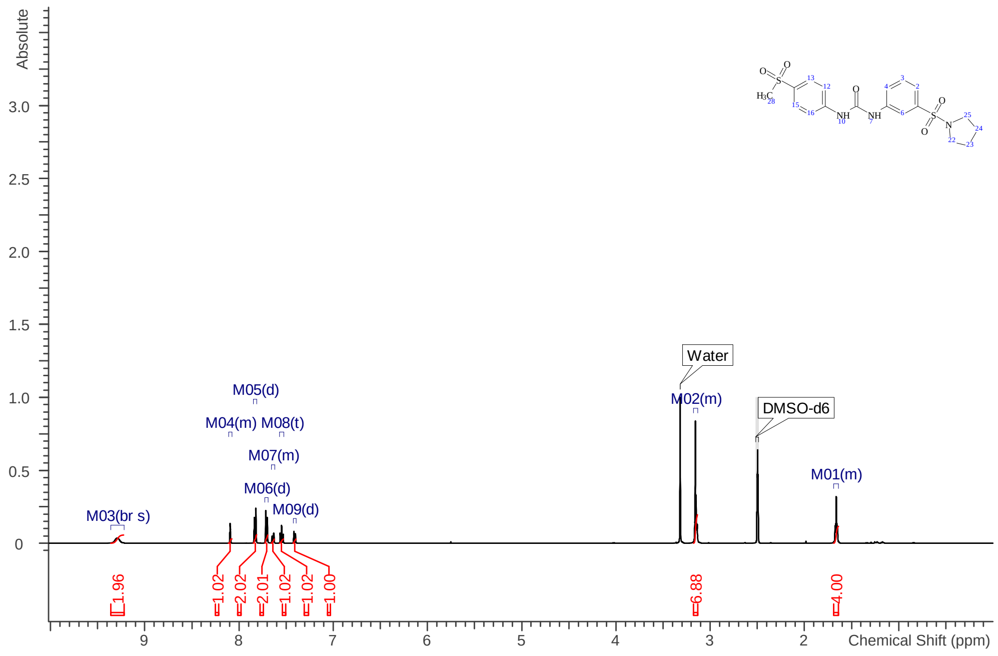
Absolute
Water
DMSO-d6
M01(m)
M02(m)
M03(br s)
M04(m)
M05(d)
M06(d)
M07(m)
M08(t)
M09(d)
1.96
1.02
2.02
2.01
1.02
1.02
1.00
6.88
4.00
Chemical Shift (ppm)

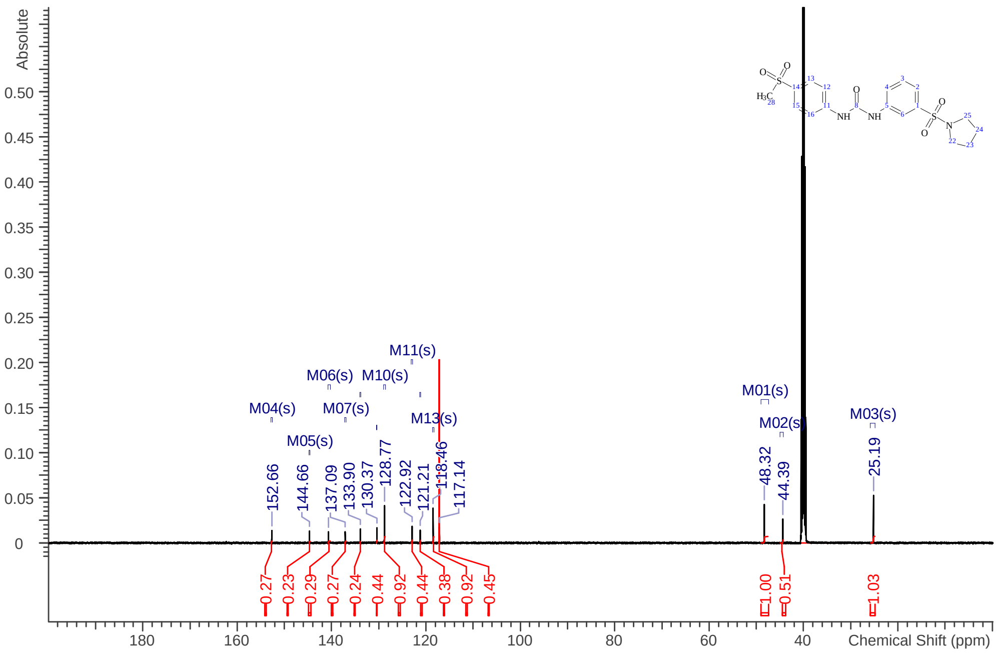
Absolute
M01(s)
M02(s)
M03(s)
M04(s)
M05(s)
M06(s)
M07(s)
M10(s)
M11(s)
M13(s)
152.66
144.66
137.09
133.90
130.37
128.77
122.92
121.21
118.46
117.14
48.32
44.39
25.19
Chemical Shift (ppm)

10

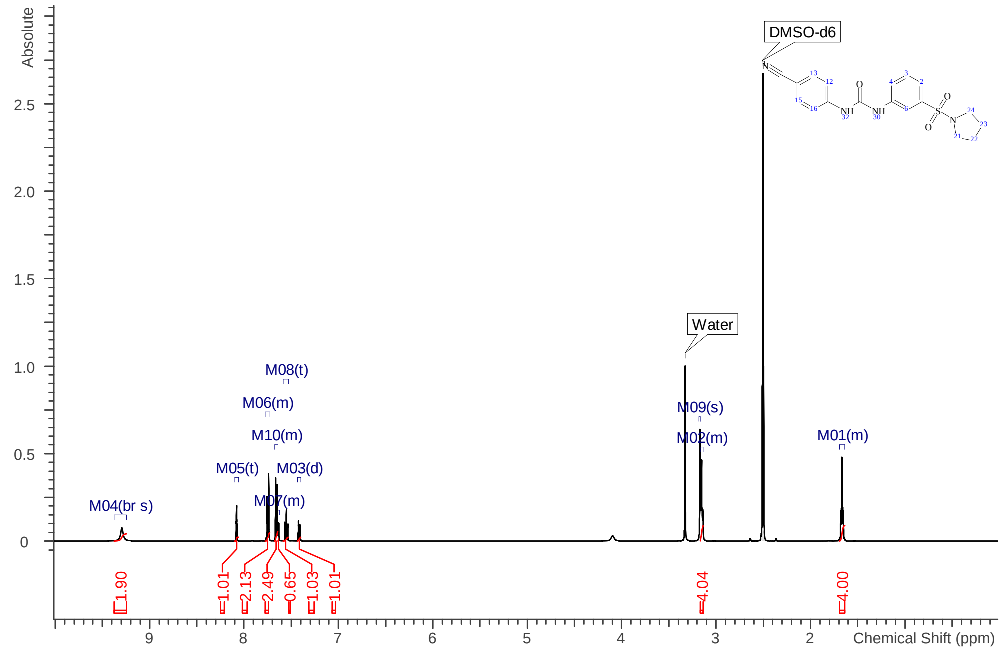

Absolute
DMSO-d6
Water
M08(t)
M06(m)
M10(m)
M05(t)
M03(d)
M04(br s)
M07(m)
M09(s)
M02(m)
M01(m)
1.90
1.01
2.13
2.49
0.65
1.03
1.01
4.04
4.00
Chemical Shift (ppm)

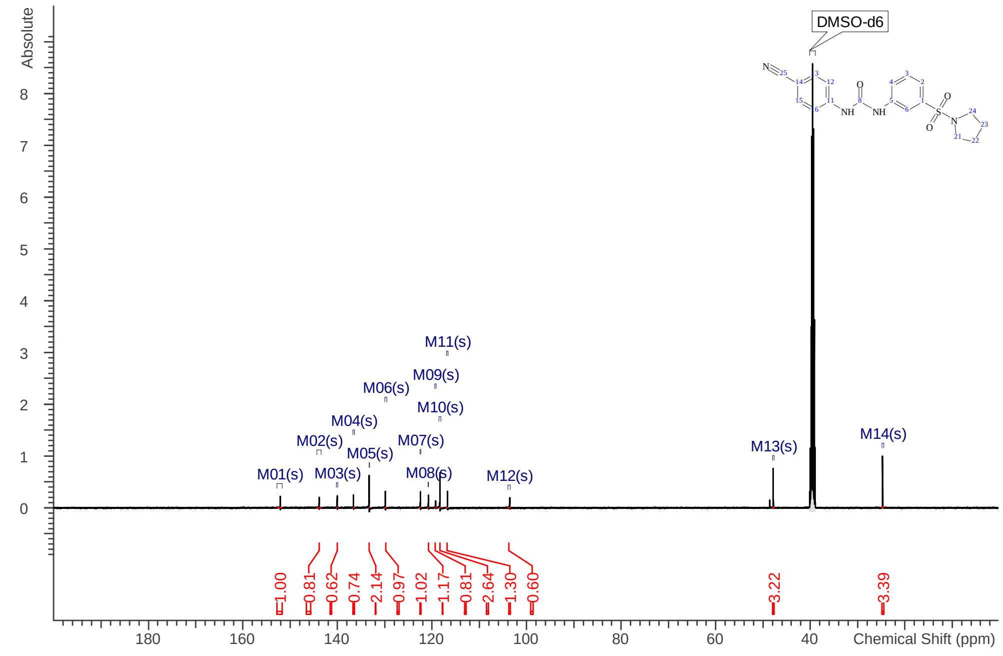

Absolute
DMSO-d6
M11(s)
M09(s)
M06(s)
M10(s)
M04(s)
M02(s)
M07(s)
M05(s)
M01(s)
M03(s)
M08(s)
M12(s)
M13(s)
M14(s)
1.00
0.81
0.62
0.74
2.14
0.97
1.02
1.17
0.81
2.64
1.30
0.60
3.22
3.39
Chemical Shift (ppm)

11

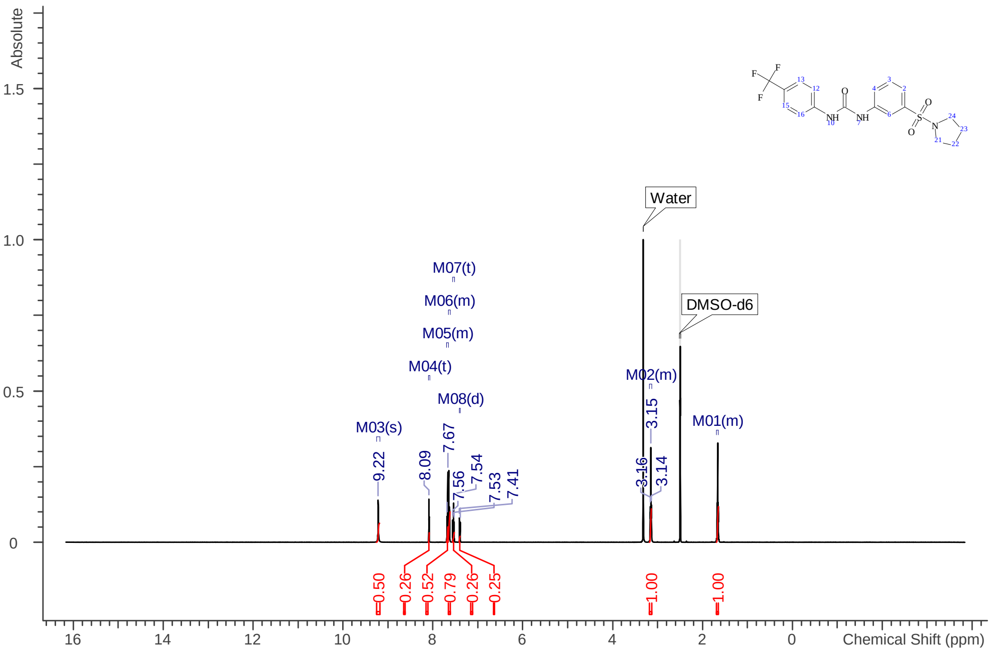

Absolute
Water
DMSO-d6
M07(t)
M06(m)
M05(m)
M04(t)
M08(d)
M03(s)
M02(m)
M01(m)
9.22
8.09
7.67
7.56
7.54
7.53
7.41
3.16
3.15
3.14
0.50
0.26
0.52
0.79
0.26
0.25
1.00
1.00
Chemical Shift (ppm)

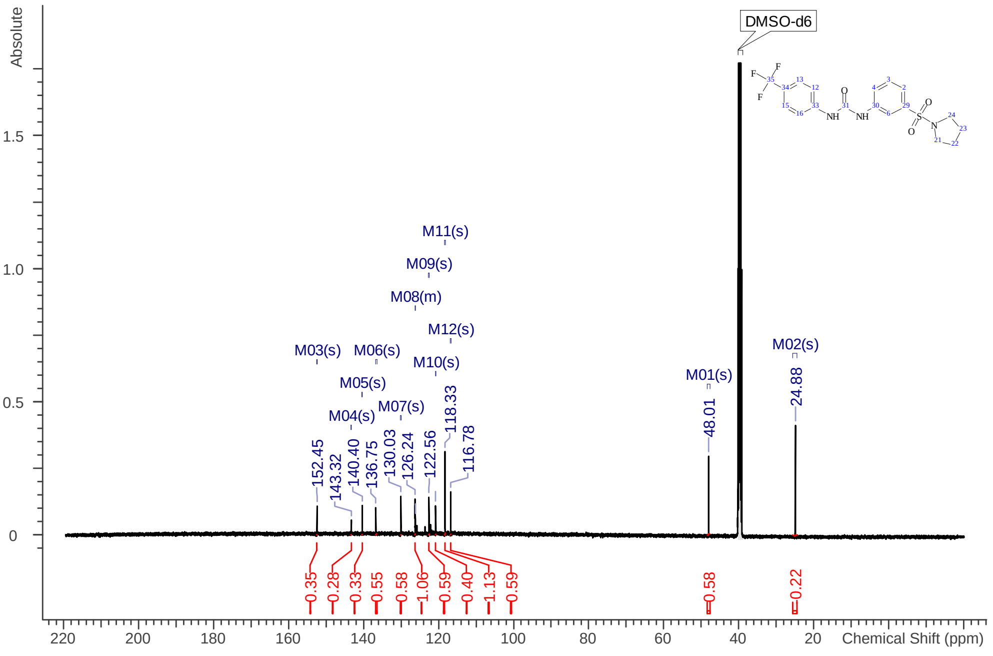

Absolute
DMSO-d6
M11(s)
M09(s)
M08(m)
M12(s)
M03(s)
M06(s)
M10(s)
M05(s)
M07(s)
M04(s)
M01(s)
M02(s)
152.45
143.32
140.40
136.75
130.03
126.24
122.56
118.33
116.78
48.01
24.88
0.35
0.28
0.33
0.55
0.58
1.06
0.59
0.40
1.13
0.59
0.58
0.22
Chemical Shift (ppm)

12

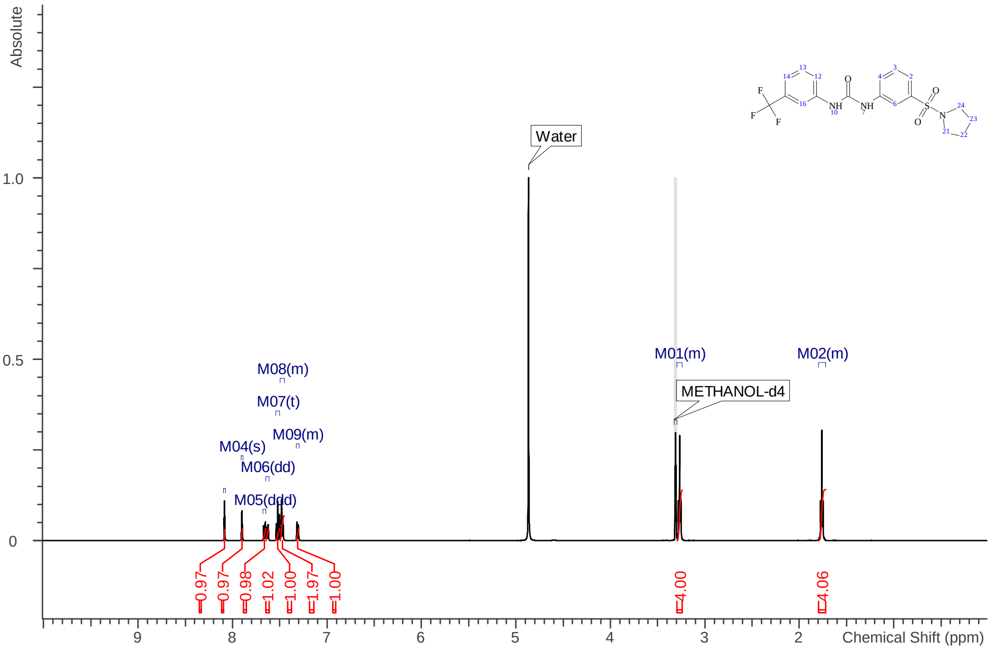
Absolute
1.0
0.5
0
Water
METHANOL-d4
M01(m)
M02(m)
M04(s)
M05(ddd)
M06(dd)
M07(t)
M08(m)
M09(m)
0.97
0.97
0.98
1.02
1.00
1.97
1.00
4.00
4.06
9
8
7
6
5
4
3
2
Chemical Shift (ppm)

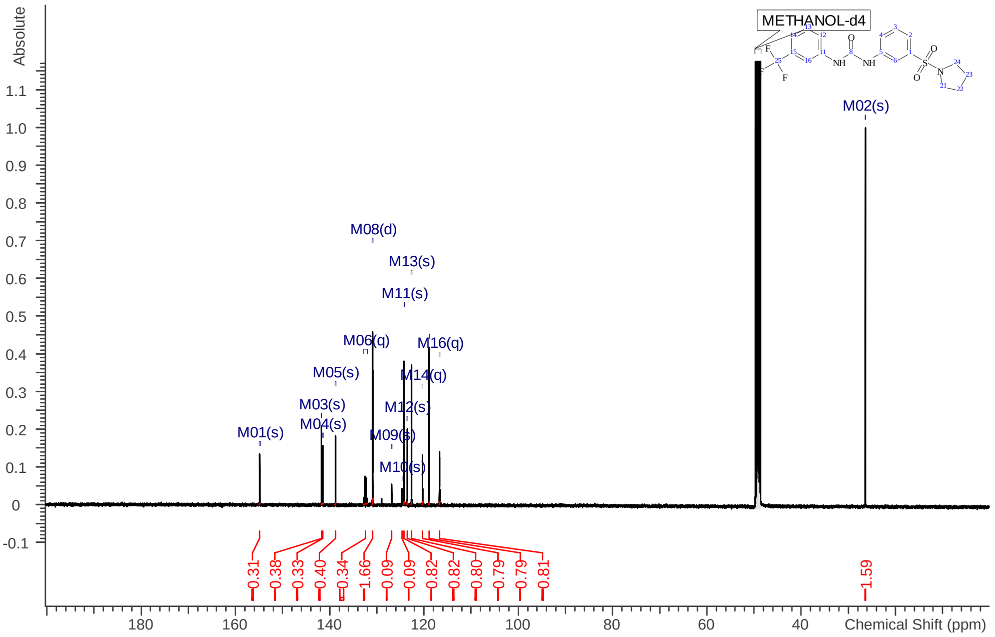
Absolute
1.1
1.0
0.9
0.8
0.7
0.6
0.5
0.4
0.3
0.2
0.1
0
-0.1
METHANOL-d4
M01(s)
M02(s)
M03(s)
M04(s)
M05(s)
M06(q)
M08(d)
M09(s)
M10(s)
M11(s)
M12(s)
M13(s)
M14(q)
M16(q)
0.31
0.38
0.33
0.40
0.34
1.66
0.09
0.09
0.82
0.82
0.80
0.79
0.79
0.81
1.59
180
160
140
120
100
80
60
40
Chemical Shift (ppm)

13

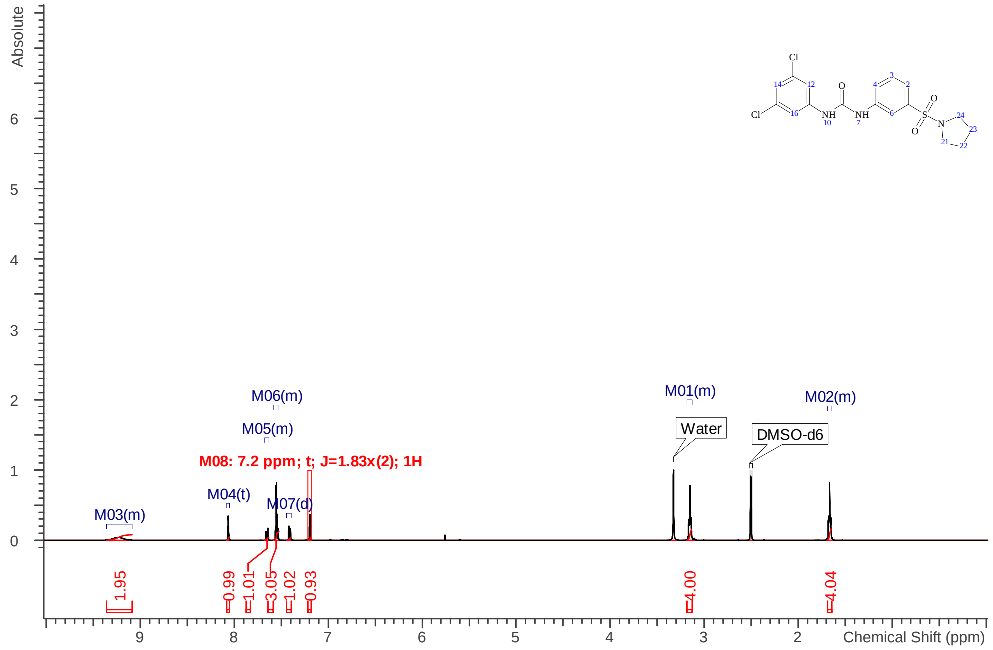

Absolute
M06(m)
M05(m)
M08: 7.2 ppm; t; J=1.83x(2); 1H
M04(t)
M07(d)
M03(m)
M01(m)
Water
DMSO-d6
M02(m)
1.95
0.99
1.01
3.05
1.02
0.93
4.00
4.04
Chemical Shift (ppm)

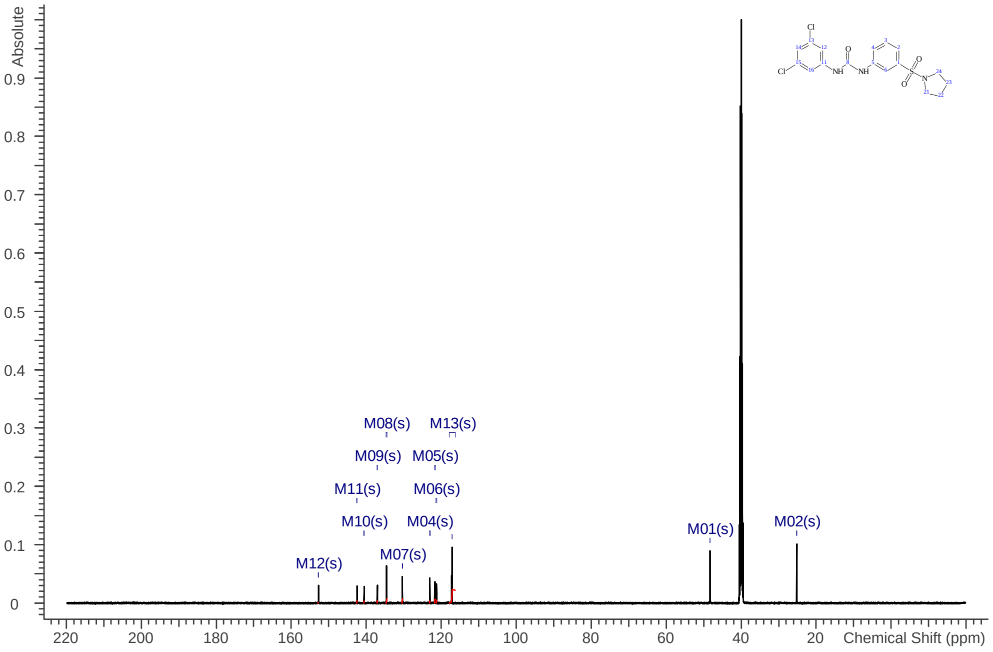

Absolute
M08(s)
M13(s)
M09(s)
M05(s)
M11(s)
M06(s)
M10(s)
M04(s)
M07(s)
M12(s)
M01(s)
M02(s)
Chemical Shift (ppm)

14

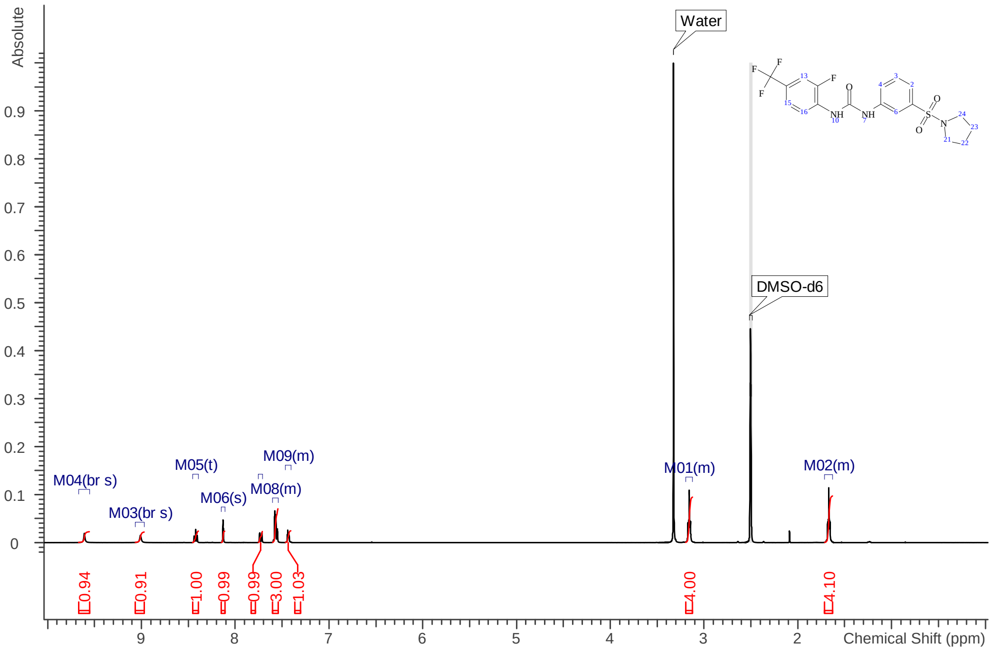
Absolute
Water
DMSO-d6
M04(br s)
M03(br s)
M05(t)
M06(s)
M09(m)
M08(m)
M01(m)
M02(m)
0.94
0.91
1.00
0.99
0.99
3.00
1.03
4.00
4.10
Chemical Shift (ppm)

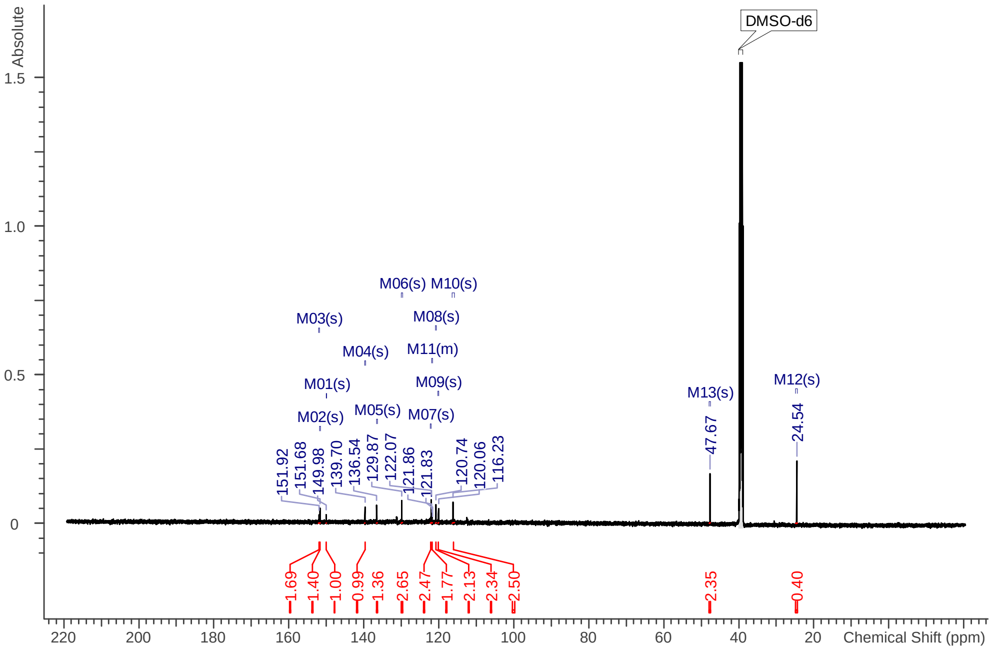
Absolute
DMSO-d6
M06(s) M10(s)
M03(s)
M08(s)
M04(s)
M11(m)
M01(s)
M09(s)
M05(s)
M07(s)
M02(s)
M13(s)
M12(s)
151.92
151.68
149.98
139.70
136.54
129.87
122.07
121.86
121.83
120.74
120.06
116.23
47.67
24.54
1.69
1.40
1.00
0.99
1.36
2.65
2.47
1.77
2.13
2.34
2.50
2.35
0.40
Chemical Shift (ppm)

15

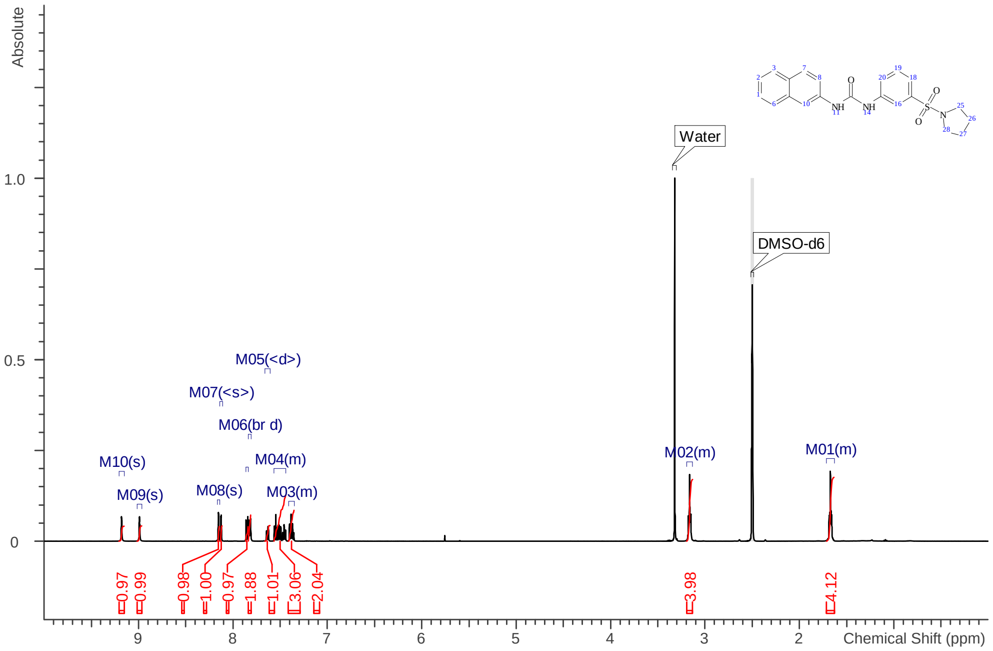
Water
DMSO-d6
M10(s)
M09(s)
M08(s)
M07(<s>)
M06(br d)
M05(<d>)
M04(m)
M03(m)
M02(m)
M01(m)
0.97
0.99
0.98
1.00
0.97
1.88
1.01
3.06
2.04
3.98
4.12
Absolute
Chemical Shift (ppm)

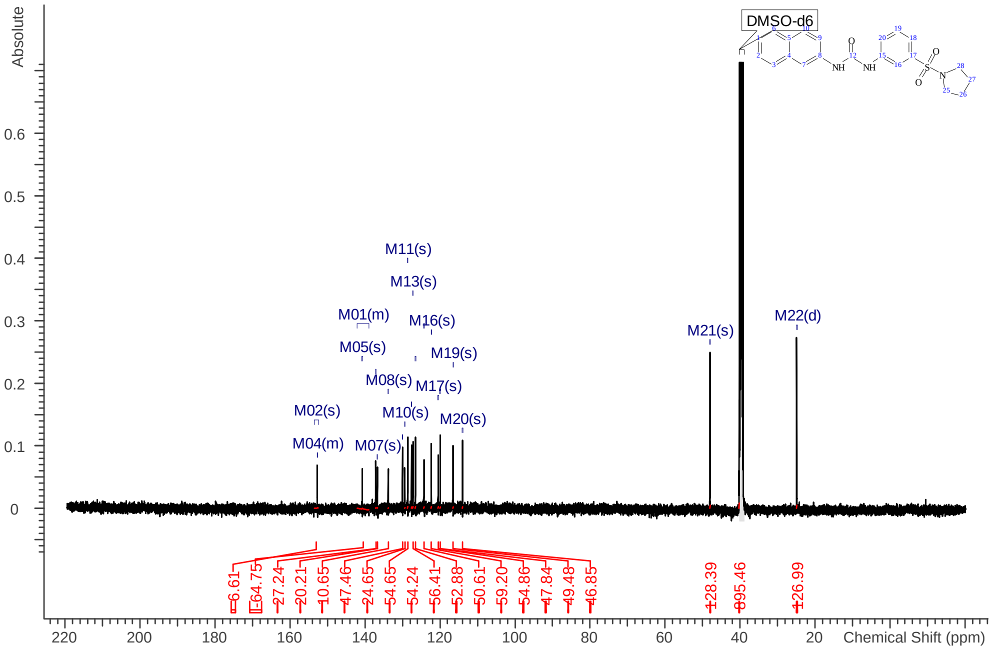
DMSO-d6
M11(s)
M13(s)
M01(m)
M16(s)
M05(s)
M19(s)
M08(s)
M17(s)
M02(s)
M10(s)
M20(s)
M04(m)
M07(s)
M21(s)
M22(d)
Absolute
Chemical Shift (ppm)

16

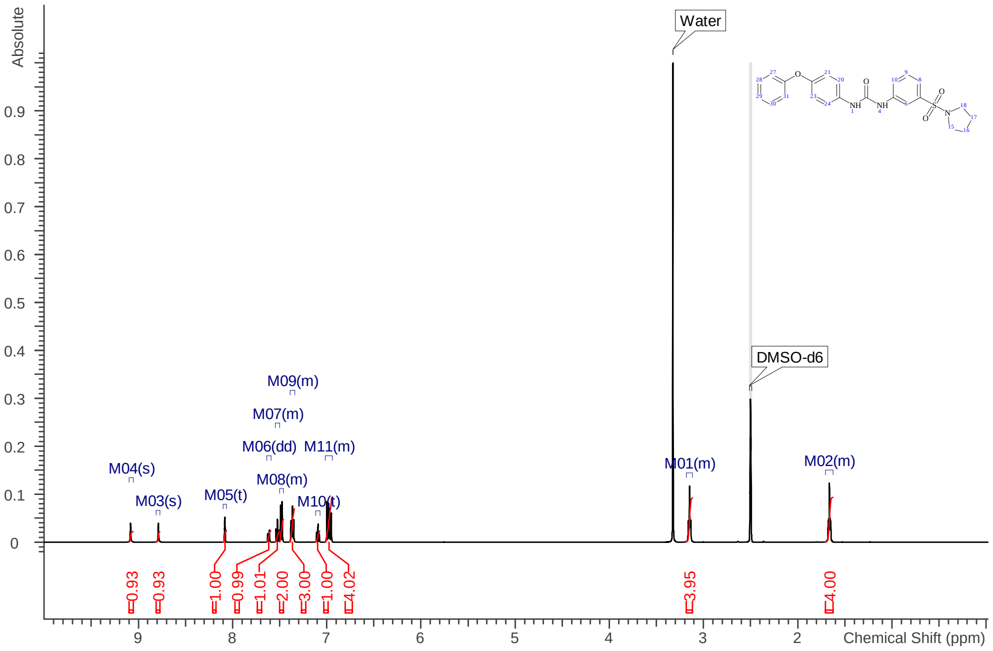

Absolute
Water
DMSO-d6
M04(s)
M03(s)
M05(t)
M06(dd)
M07(m)
M08(m)
M09(m)
M10(t)
M11(m)
M01(m)
M02(m)
0.93
0.93
1.00
0.99
1.01
2.00
3.00
1.00
4.02
3.95
4.00
Chemical Shift (ppm)

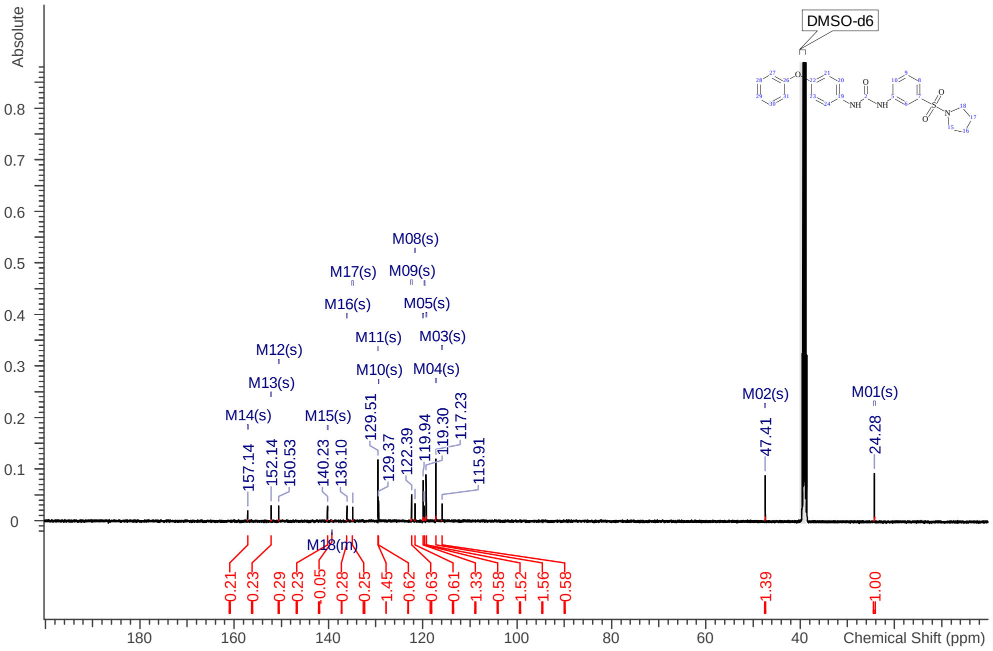

Absolute
DMSO-d6
M14(s)
M13(s)
M12(s)
M15(s)
M16(s)
M17(s)
M18(m)
M10(s)
M11(s)
M08(s)
M09(s)
M05(s)
M03(s)
M04(s)
M02(s)
M01(s)
157.14
152.14
150.53
140.23
136.10
129.51
129.37
122.39
119.94
119.30
117.23
115.91
47.41
24.28
0.21
0.23
0.29
0.23
0.05
0.28
0.25
1.45
0.62
0.63
0.61
1.33
0.58
1.52
1.56
0.58
1.39
1.00
Chemical Shift (ppm)

17

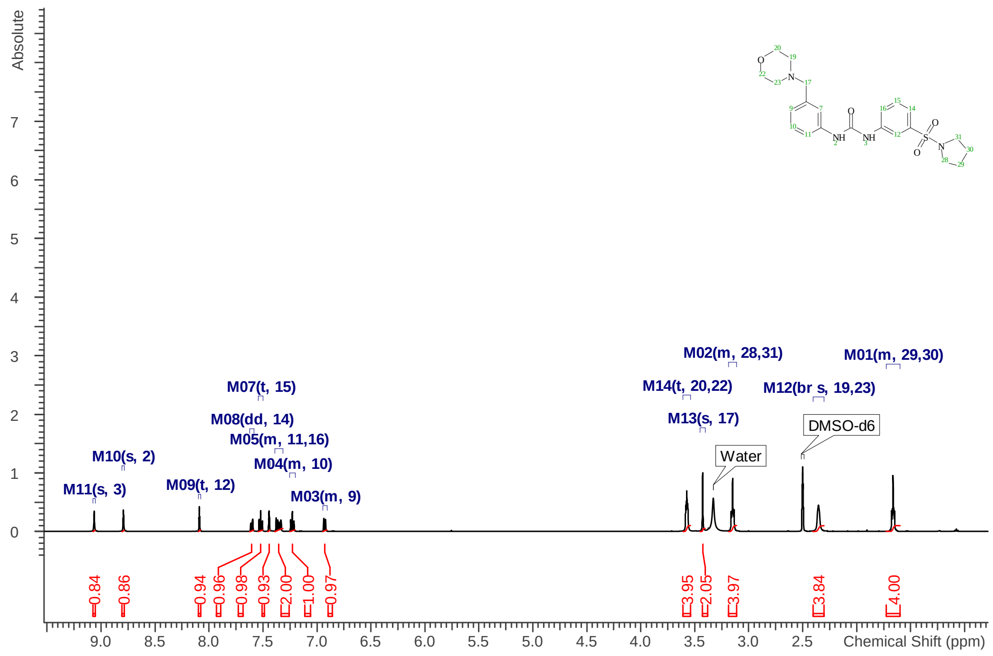

Absolute
M11(s, 3)
M10(s, 2)
M09(t, 12)
M08(dd, 14)
M07(t, 15)
M05(m, 11,16)
M04(m, 10)
M03(m, 9)
M14(t, 20,22)
M13(s, 17)
M02(m, 28,31)
M12(br s, 19,23)
M01(m, 29,30)
Water
DMSO-d6
0.84
0.86
0.94
0.96
0.98
0.93
2.00
1.00
0.97
3.95
2.05
3.97
3.84
4.00
Chemical Shift (ppm)

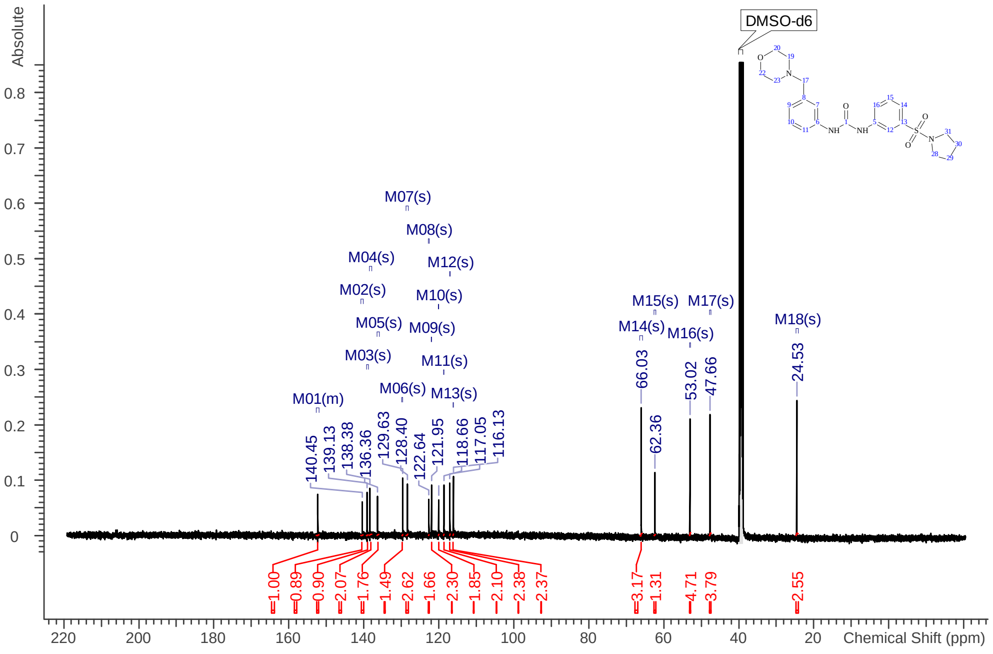

Absolute
DMSO-d6
M01(m)
M02(s)
M03(s)
M04(s)
M05(s)
M06(s)
M07(s)
M08(s)
M09(s)
M10(s)
M11(s)
M12(s)
M13(s)
M14(s)
M15(s)
M16(s)
M17(s)
M18(s)
140.45
139.13
138.38
136.36
129.63
128.40
122.64
121.95
118.66
117.05
116.13
66.03
62.36
53.02
47.66
24.53
1.00
0.89
0.90
2.07
1.76
1.49
2.62
1.66
2.30
1.85
2.10
2.38
2.37
3.17
1.31
4.71
3.79
2.55
Chemical Shift (ppm)

18

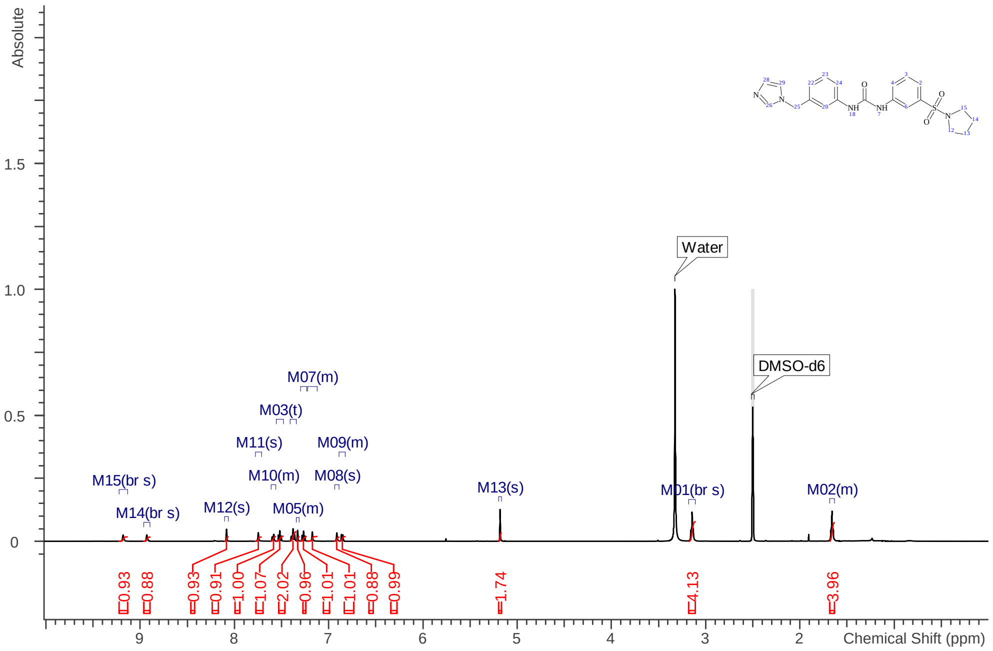

Absolute
1.5
1.0
0.5
0
Water
DMSO-d6
M07(m)
M03(t)
M11(s)
M09(m)
M15(br s)
M10(m)
M08(s)
M13(s)
M01(br s)
M02(m)
M14(br s)
M12(s)
M05(m)
0.93
0.88
0.93
0.91
1.00
1.07
2.02
0.96
1.01
1.01
0.88
0.99
1.74
4.13
3.96
9
8
7
6
5
4
3
2
Chemical Shift (ppm)

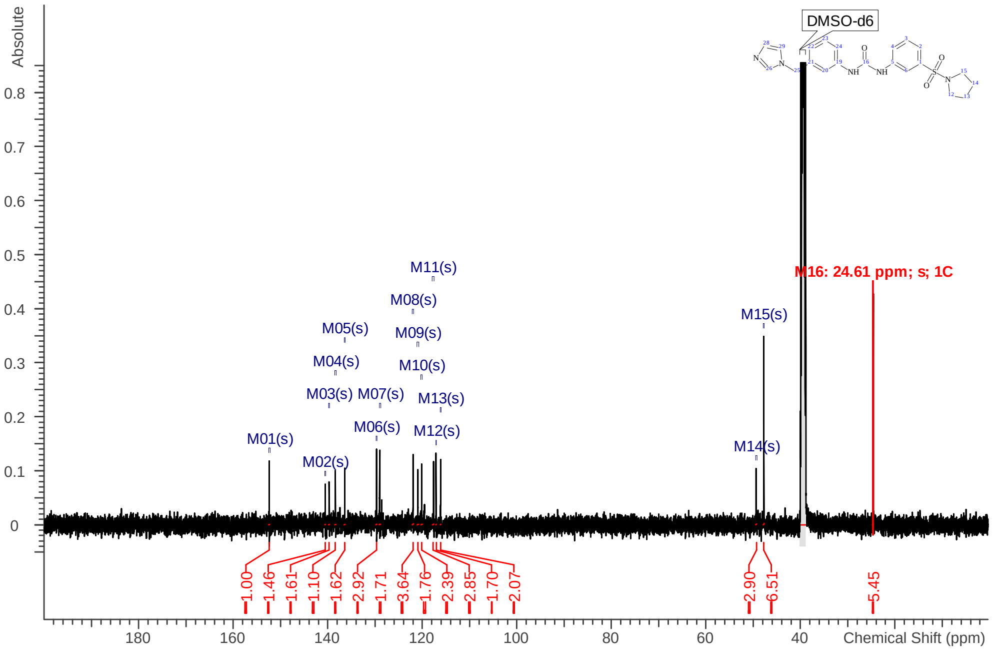

Absolute
0.8
0.7
0.6
0.5
0.4
0.3
0.2
0.1
0
DMSO-d6
M11(s)
M16: 24.61 ppm; s; 1C
M08(s)
M05(s)
M09(s)
M15(s)
M04(s)
M10(s)
M03(s)
M07(s)
M13(s)
M06(s)
M12(s)
M01(s)
M14(s)
M02(s)
1.00
1.46
1.61
1.10
1.62
2.92
1.71
3.64
1.76
2.39
2.85
1.70
2.07
2.90
6.51
5.45
180
160
140
120
100
80
60
40
Chemical Shift (ppm)

21

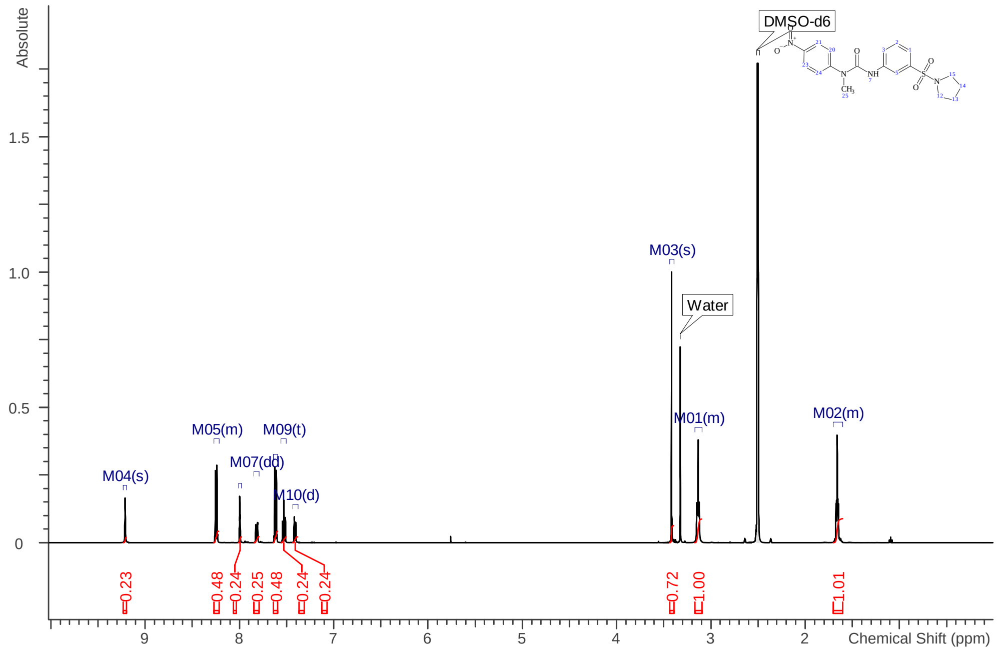

DMSO-d6
Water
M04(s)
M05(m)
M07(dd)
M09(t)
M10(d)
M03(s)
M01(m)
M02(m)
0.23
0.48
0.24
0.25
0.48
0.24
0.24
0.72
1.00
1.01
Absolute
Chemical Shift (ppm)

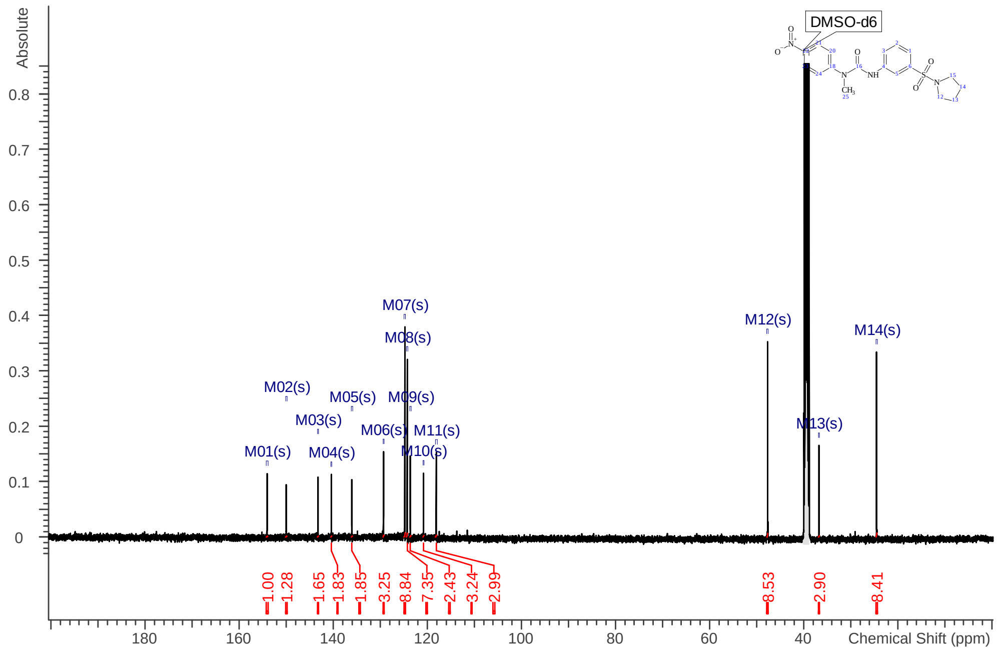

DMSO-d6
M01(s)
M02(s)
M03(s)
M04(s)
M05(s)
M06(s)
M07(s)
M08(s)
M09(s)
M10(s)
M11(s)
M12(s)
M13(s)
M14(s)
1.00
1.28
1.65
1.83
1.85
3.25
8.84
7.35
2.43
3.24
2.99
8.53
2.90
8.41
Absolute
Chemical Shift (ppm)

21

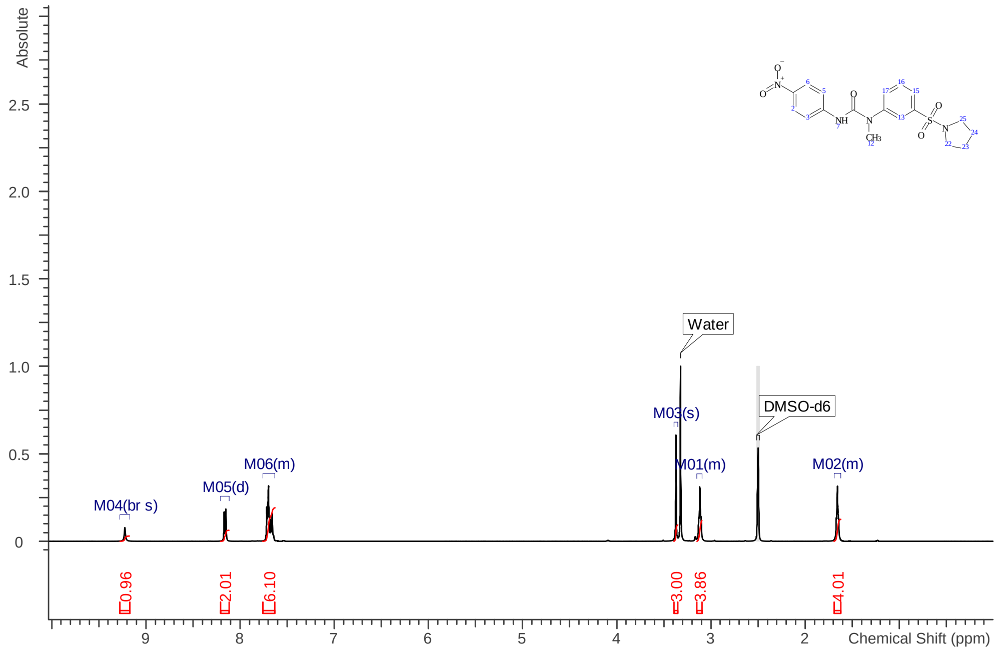

Absolute
Water
DMSO-d6
M04(br s)
M05(d)
M06(m)
M03(s)
M01(m)
M02(m)
0.96
2.01
6.10
3.00
3.86
4.01
Chemical Shift (ppm)

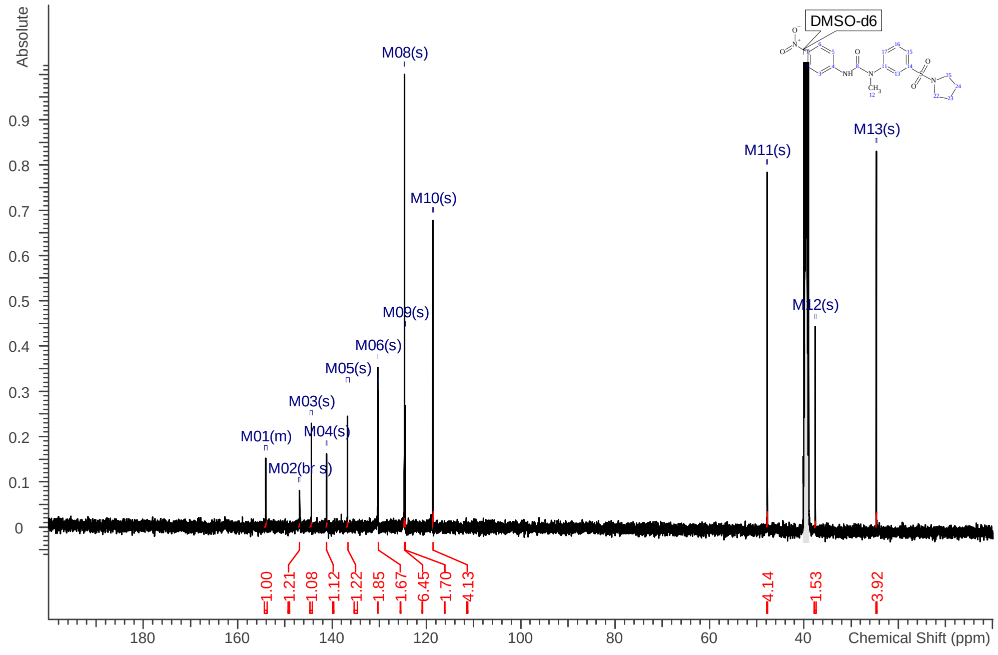

Absolute
DMSO-d6
M08(s)
M13(s)
M11(s)
M10(s)
M12(s)
M09(s)
M06(s)
M05(s)
M03(s)
M04(s)
M01(m)
M02(br s)
1.00
1.21
1.08
1.12
1.22
1.85
1.67
6.45
1.70
4.13
4.14
1.53
3.92
Chemical Shift (ppm)

23

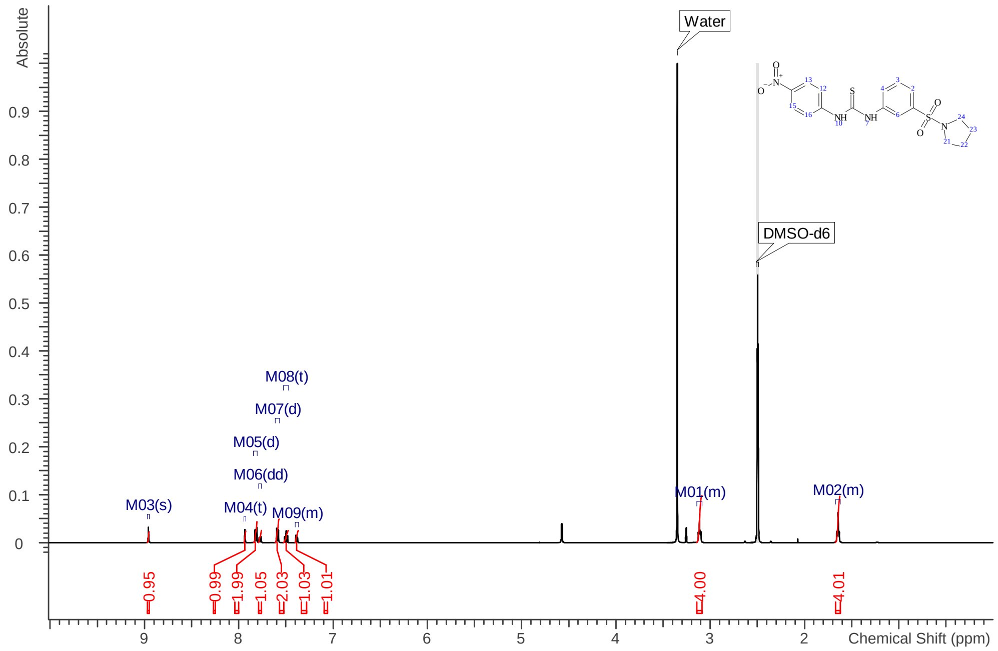

Absolute
Water
DMSO-d6
M08(t)
M07(d)
M05(d)
M06(dd)
M03(s)
M04(t)
M09(m)
M01(m)
M02(m)
0.95
0.99
1.99
1.05
2.03
1.03
1.01
4.00
4.01
Chemical Shift (ppm)

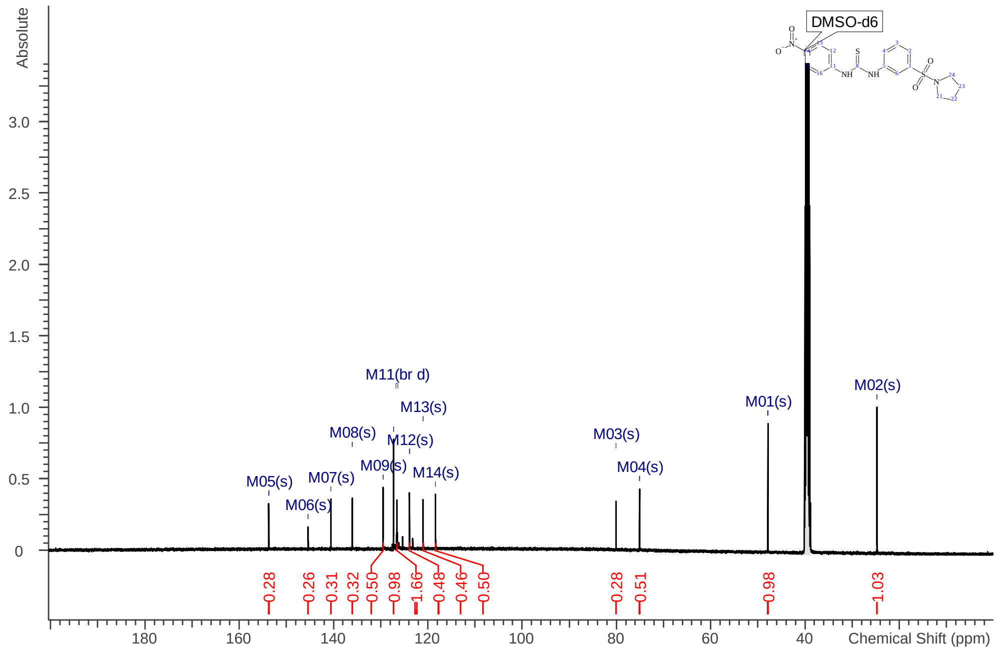

Absolute
DMSO-d6
M11(br d)
M13(s)
M08(s)
M12(s)
M09(s)
M14(s)
M05(s)
M07(s)
M06(s)
M03(s)
M04(s)
M01(s)
M02(s)
0.28
0.26
0.31
0.32
0.50
0.98
1.66
0.48
0.46
0.50
0.28
0.51
0.98
1.03
Chemical Shift (ppm)

24

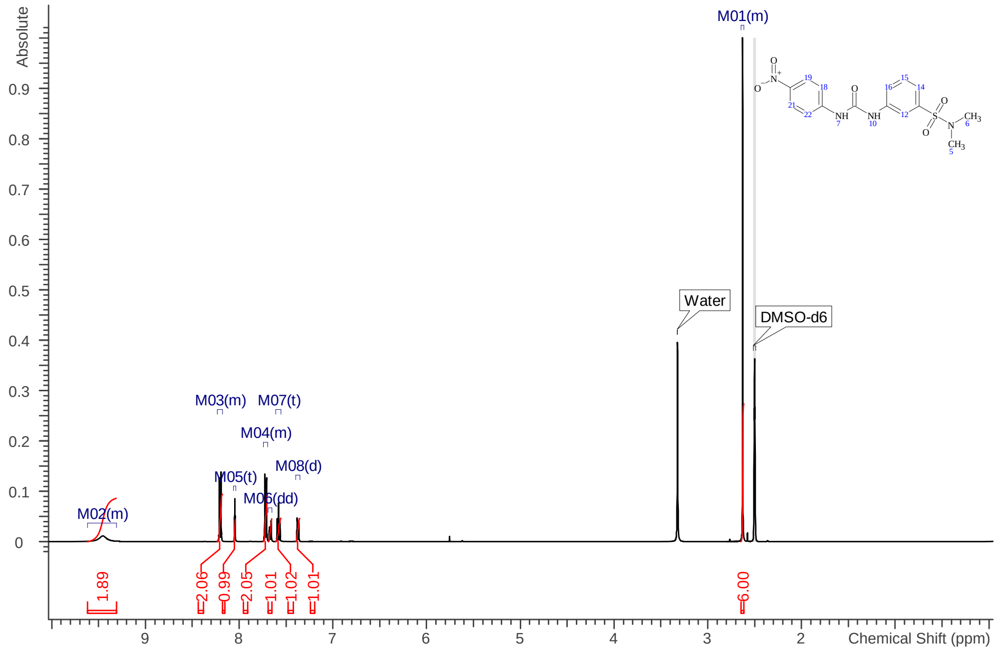

Absolute
Chemical Shift (ppm)
M01(m)
M02(m)
M03(m)
M04(m)
M05(t)
M06(dd)
M07(t)
M08(d)
Water
DMSO-d6
1.89
2.06
0.99
2.05
1.01
1.02
1.01
6.00

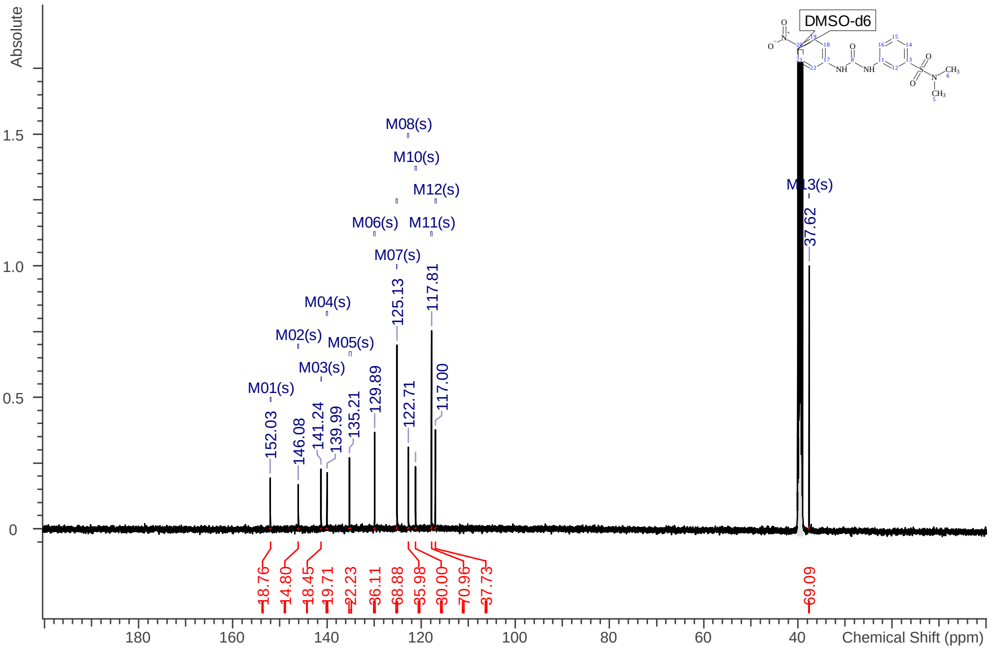

Absolute
Chemical Shift (ppm)
DMSO-d6
M01(s)
M02(s)
M03(s)
M04(s)
M05(s)
M06(s)
M07(s)
M08(s)
M10(s)
M11(s)
M12(s)
M13(s)
152.03
146.08
141.24
139.99
135.21
129.89
125.13
122.71
117.81
117.00
37.62

25

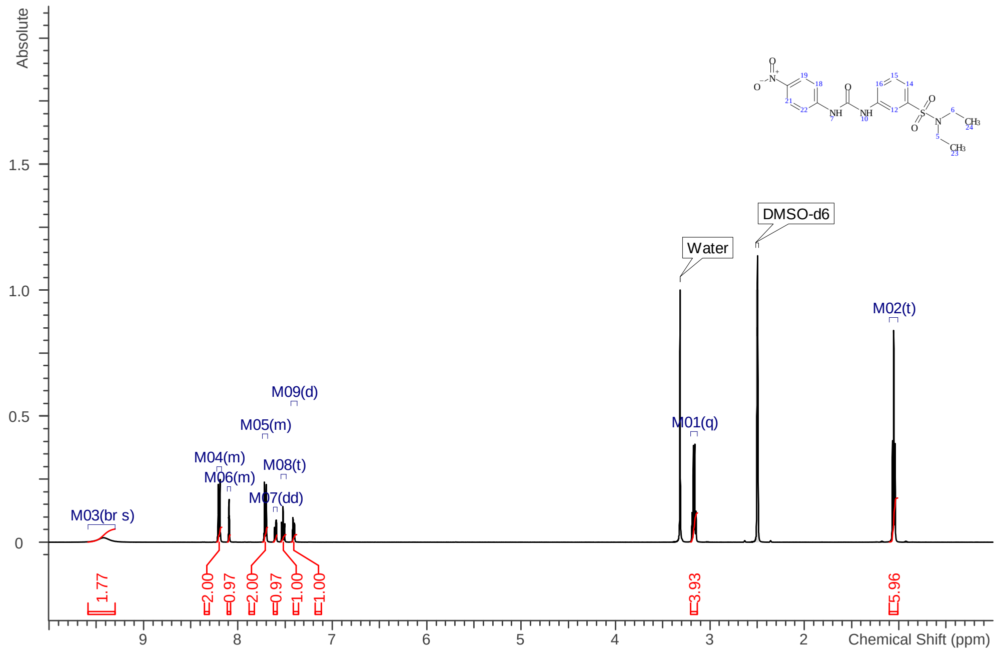
Absolute
Water
DMSO-d6
M02(t)
M09(d)
M05(m)
M01(q)
M04(m)
M06(m)
M08(t)
M07(dd)
M03(br s)
1.77
2.00
0.97
2.00
0.97
1.00
1.00
3.93
5.96
Chemical Shift (ppm)

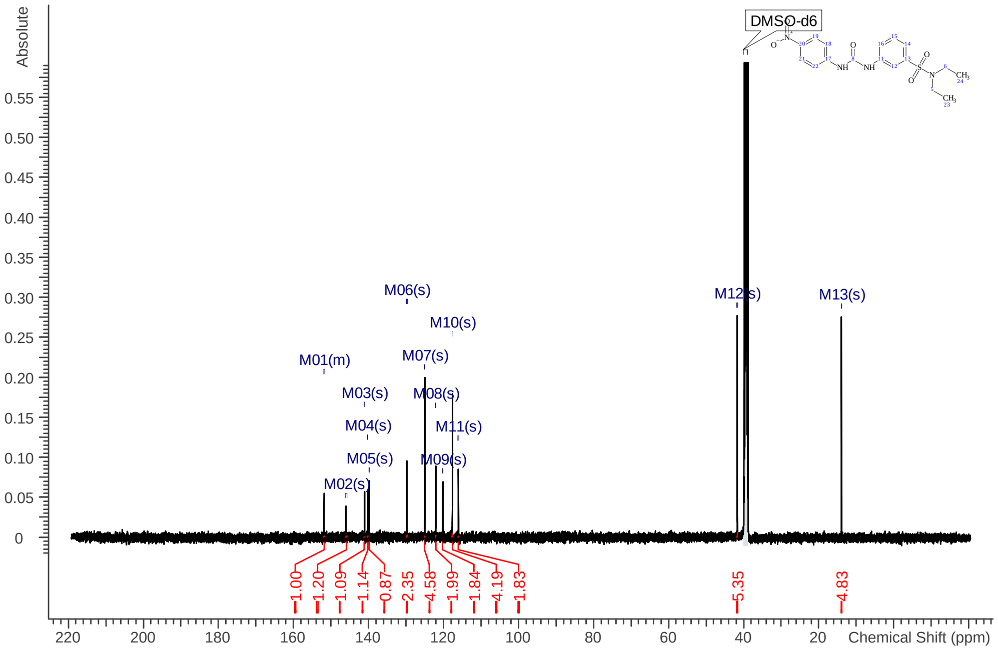
Absolute
DMSO-d6
M06(s)
M12(s)
M13(s)
M10(s)
M01(m)
M07(s)
M03(s)
M08(s)
M04(s)
M11(s)
M05(s)
M09(s)
M02(s)
1.00
1.20
1.09
1.14
0.87
2.35
4.58
1.99
1.84
4.19
1.83
5.35
4.83
Chemical Shift (ppm)

26

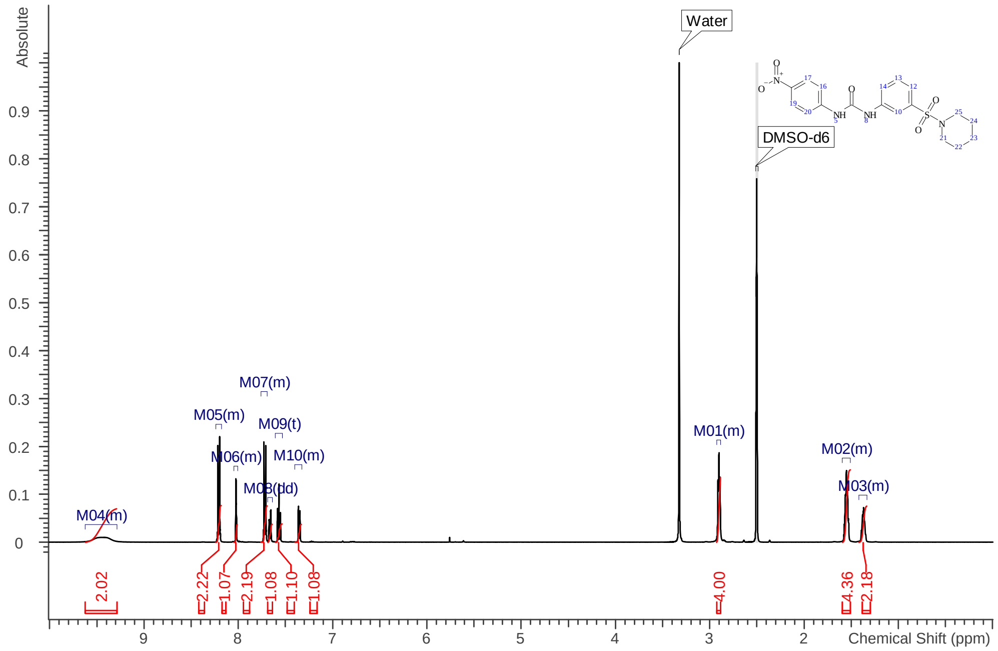

Absolute
Water
DMSO-d6
M07(m)
M05(m)
M09(t)
M06(m)
M10(m)
M08(dd)
M04(m)
M01(m)
M02(m)
M03(m)
2.02
2.22
1.07
2.19
1.08
1.10
1.08
4.00
4.36
2.18
Chemical Shift (ppm)

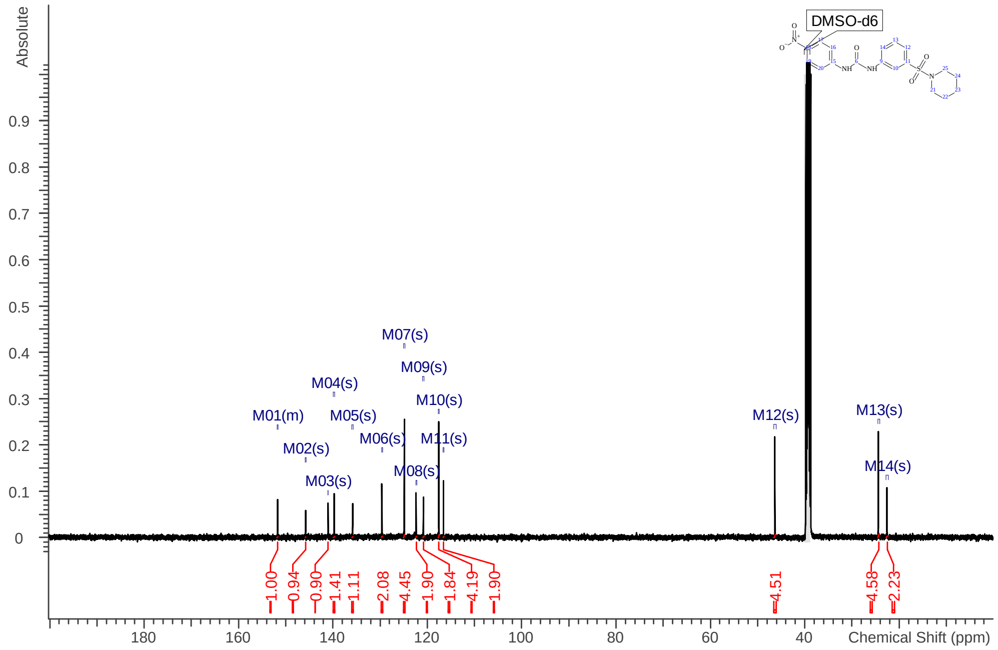

Absolute
DMSO-d6
M07(s)
M09(s)
M04(s)
M10(s)
M01(m)
M05(s)
M06(s)
M11(s)
M02(s)
M08(s)
M03(s)
M12(s)
M13(s)
M14(s)
1.00
0.94
0.90
1.41
1.11
2.08
4.45
1.90
1.84
4.19
1.90
4.51
4.58
2.23
Chemical Shift (ppm)

27

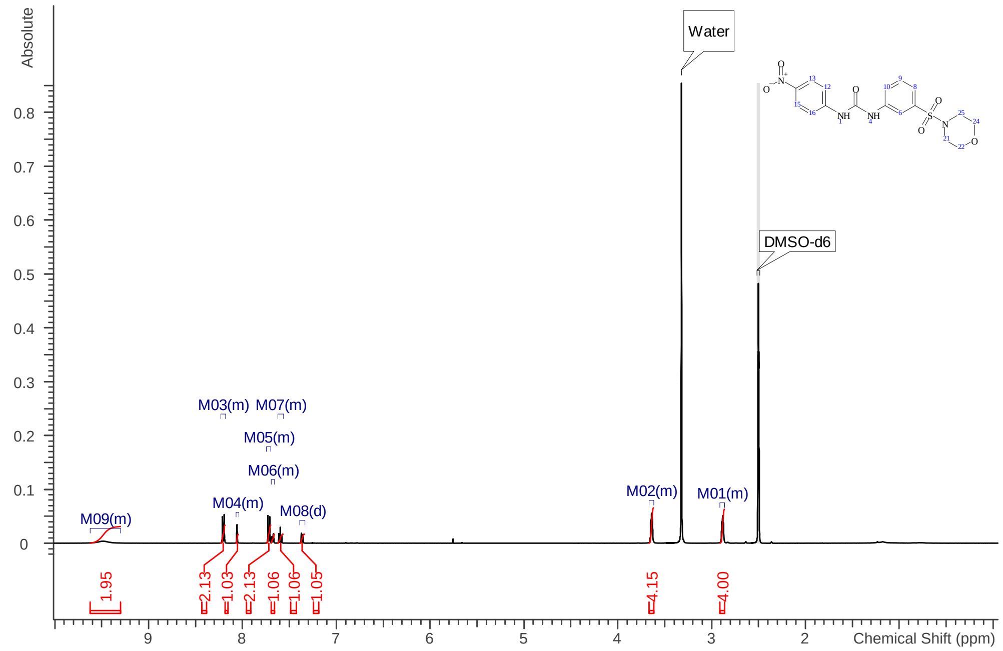
Absolute
Water
DMSO-d6
M03(m) M07(m)
M05(m)
M06(m)
M04(m)
M08(d)
M09(m)
M02(m)
M01(m)
1.95
2.13
1.03
2.13
1.06
1.06
1.05
4.15
4.00
Chemical Shift (ppm)

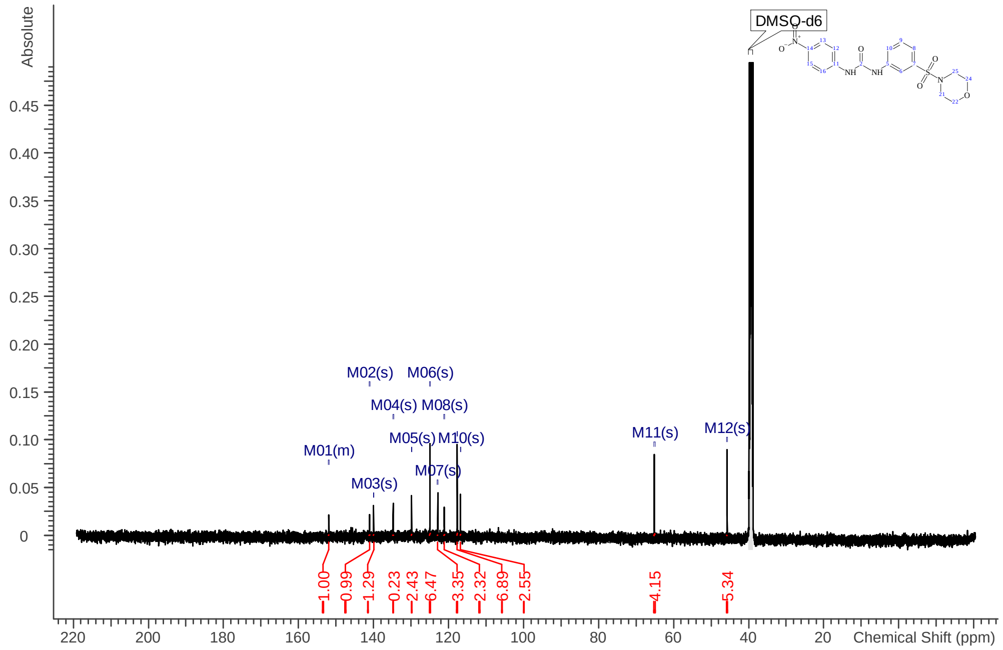
Absolute
DMSO-d6
M02(s) M06(s)
M04(s) M08(s)
M05(s) M10(s)
M01(m)
M07(s)
M03(s)
M11(s)
M12(s)
1.00
0.99
1.29
0.23
2.43
6.47
3.35
2.32
6.89
2.55
4.15
5.34
Chemical Shift (ppm)

28

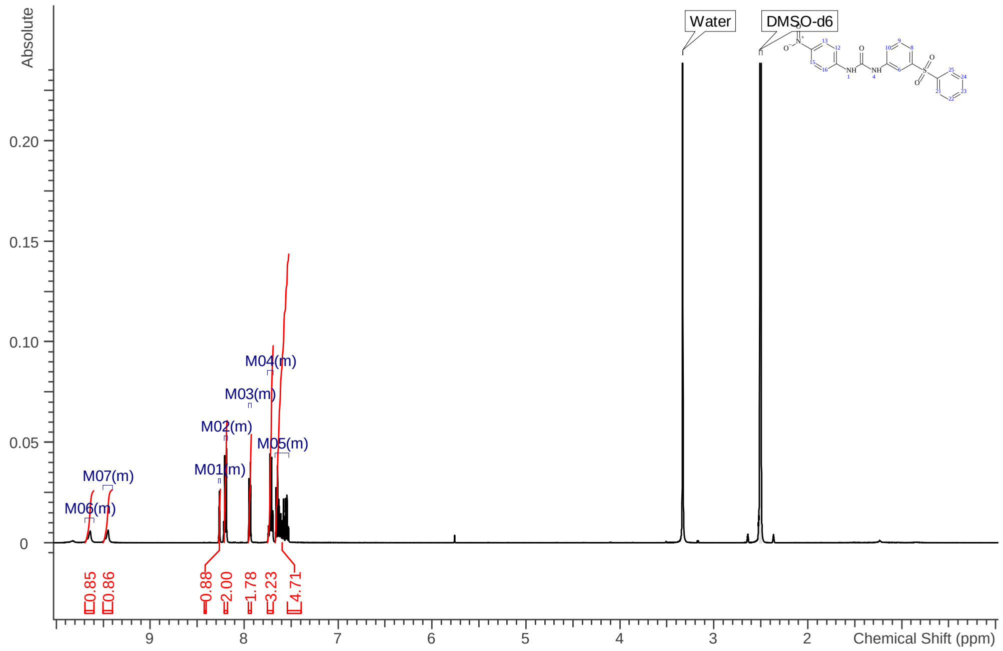
Absolute
Water
DMSO-d6
M01(m)
M02(m)
M03(m)
M04(m)
M05(m)
M06(m)
M07(m)
0.85
0.86
0.88
2.00
1.78
3.23
4.71
Chemical Shift (ppm)

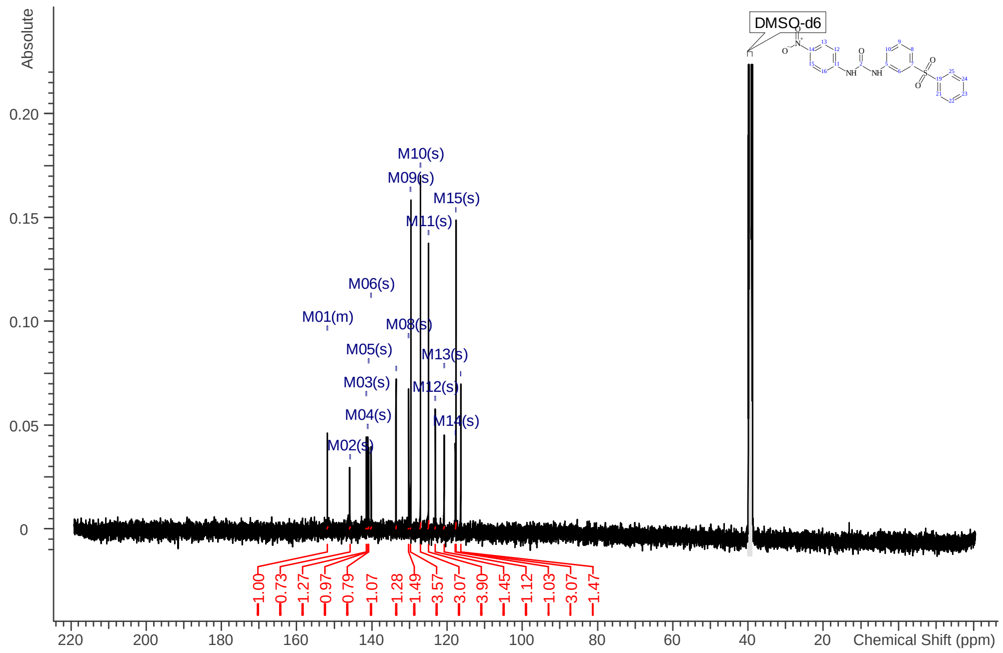
Absolute
DMSO-d6
M01(m)
M02(s)
M03(s)
M04(s)
M05(s)
M06(s)
M08(s)
M09(s)
M10(s)
M11(s)
M12(s)
M13(s)
M14(s)
M15(s)
1.00
0.73
1.27
0.97
0.79
1.07
1.28
1.49
3.57
3.07
3.90
1.45
1.12
1.03
3.07
1.47
Chemical Shift (ppm)

29

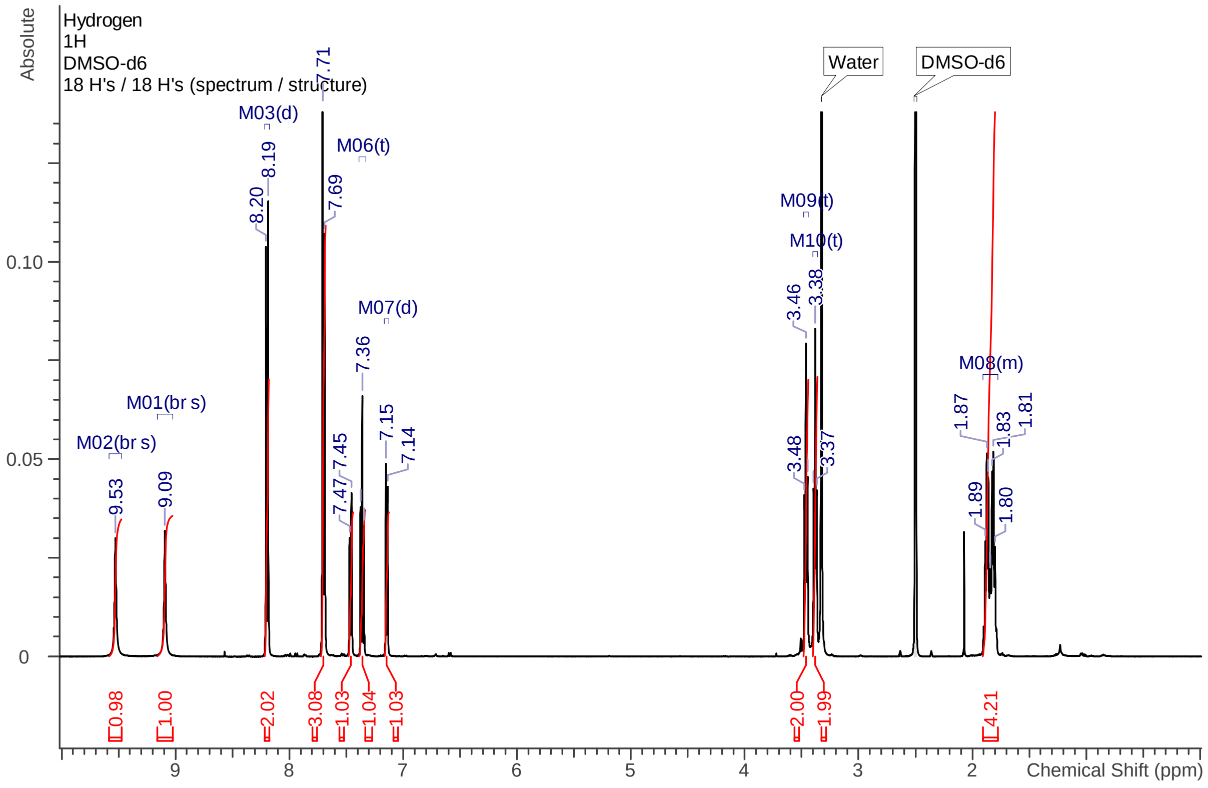
Hydrogen
1H
DMSO-d6
18 H's / 18 H's (spectrum / structure)
Absolute
Water
DMSO-d6
M01(br s)
M02(br s)
M03(d)
M06(t)
M07(d)
M08(m)
M09(t)
M10(t)
0.98
1.00
2.02
3.08
1.03
1.04
1.03
2.00
1.99
4.21
Chemical Shift (ppm)

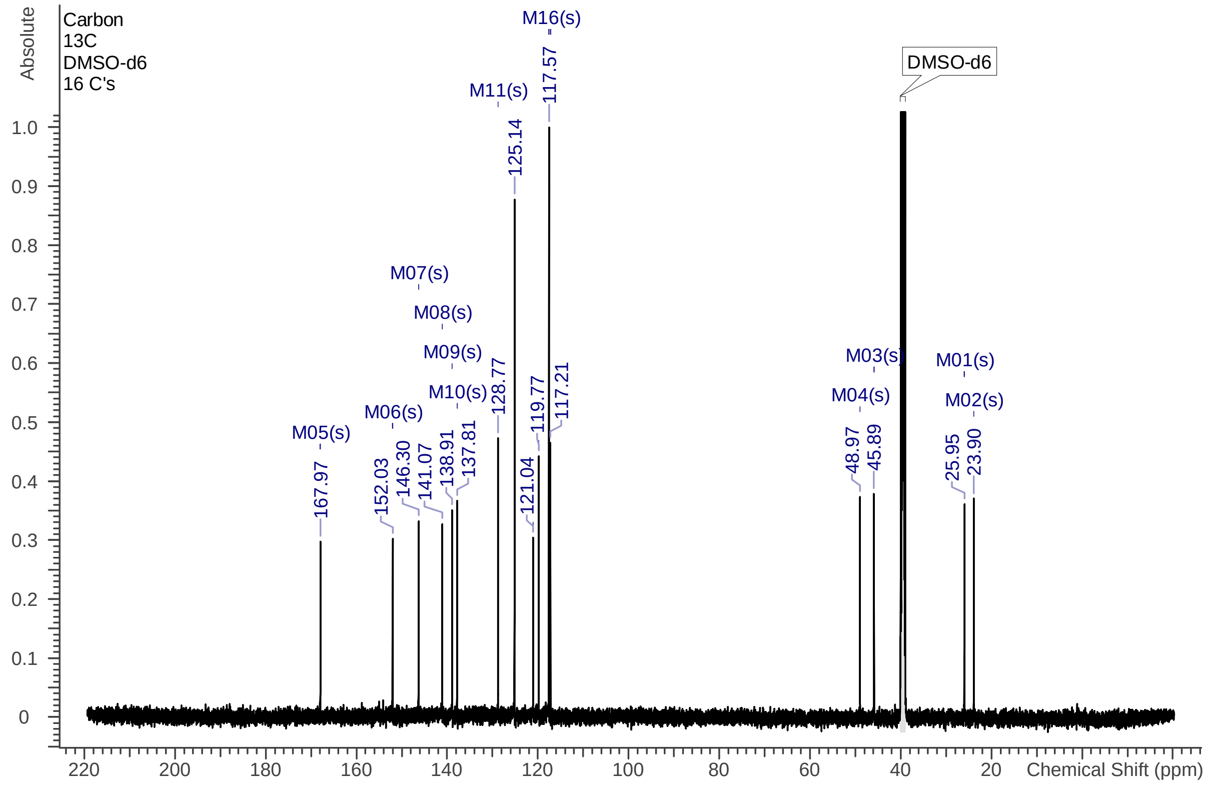
Carbon
13C
DMSO-d6
16 C's
Absolute
DMSO-d6
M01(s)
M02(s)
M03(s)
M04(s)
M05(s)
M06(s)
M07(s)
M08(s)
M09(s)
M10(s)
M11(s)
M16(s)
167.97
152.03
146.30
141.07
138.91
137.81
128.77
125.14
121.04
119.77
117.57
117.21
48.97
45.89
25.95
23.90
Chemical Shift (ppm)

30

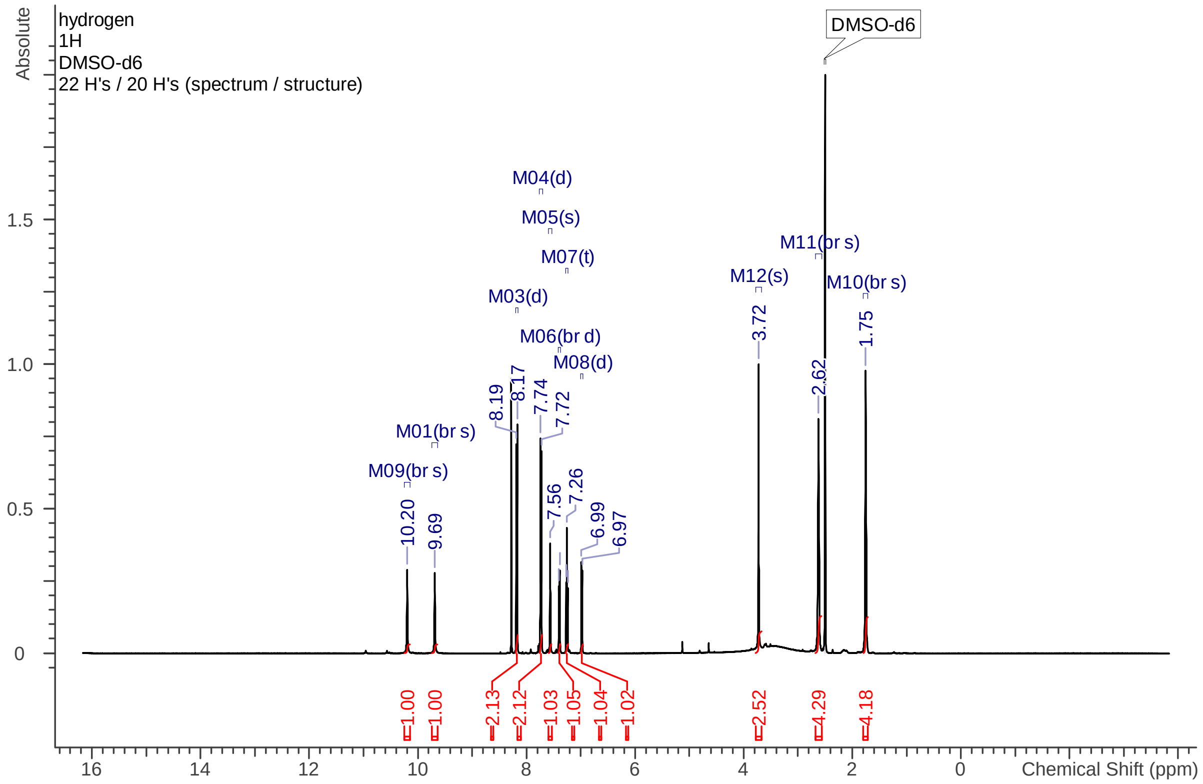
hydrogen
1H
DMSO-d6
22 H's / 20 H's (spectrum / structure)
DMSO-d6
Absolute
M04(d)
M05(s)
M07(t)
M03(d)
M06(br d)
M08(d)
M01(br s)
M09(br s)
M12(s)
M11(br s)
M10(br s)
10.20
9.69
8.19
8.17
7.74
7.72
7.56
7.26
6.99
6.97
3.72
2.62
1.75
1.00
1.00
2.13
2.12
1.03
1.05
1.04
1.02
2.52
4.29
4.18
Chemical Shift (ppm)

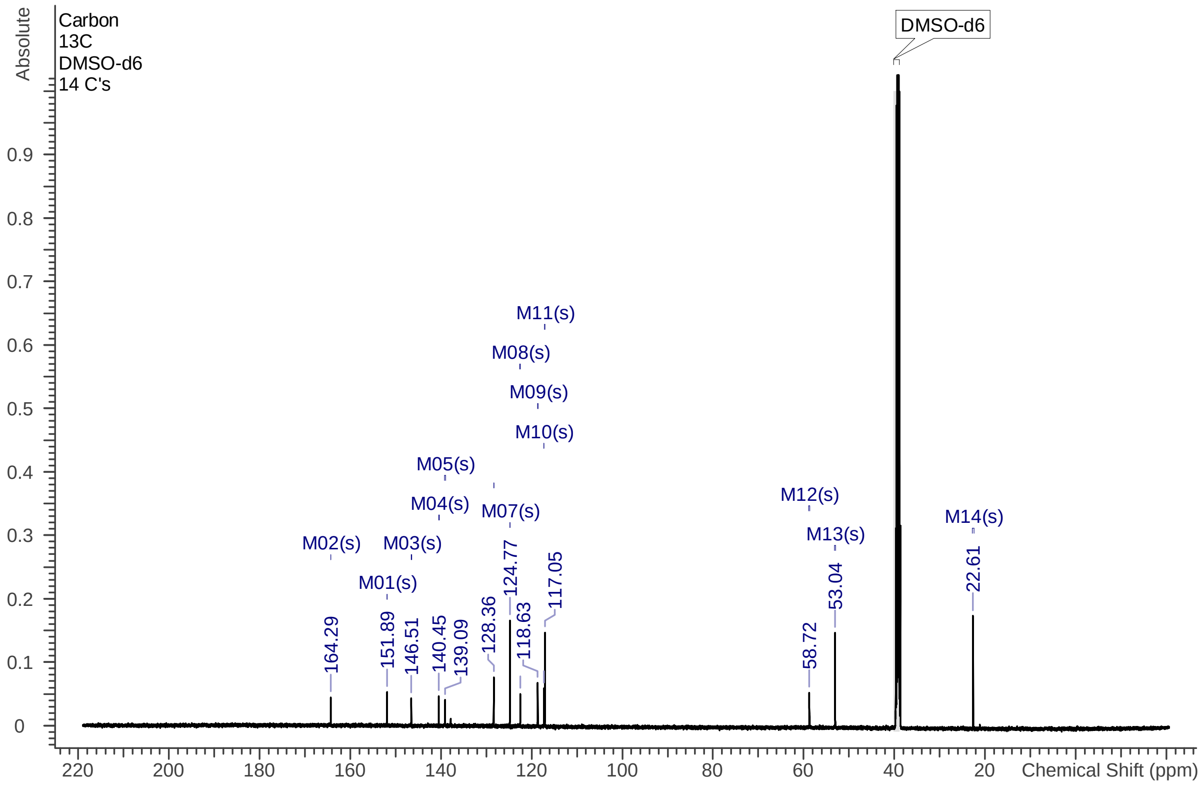
Carbon
13C
DMSO-d6
14 C's
DMSO-d6
Absolute
M11(s)
M08(s)
M09(s)
M10(s)
M05(s)
M04(s)
M07(s)
M02(s)
M03(s)
M01(s)
M12(s)
M13(s)
M14(s)
164.29
151.89
146.51
140.45
139.09
128.36
124.77
118.63
117.05
58.72
53.04
22.61
Chemical Shift (ppm)

31

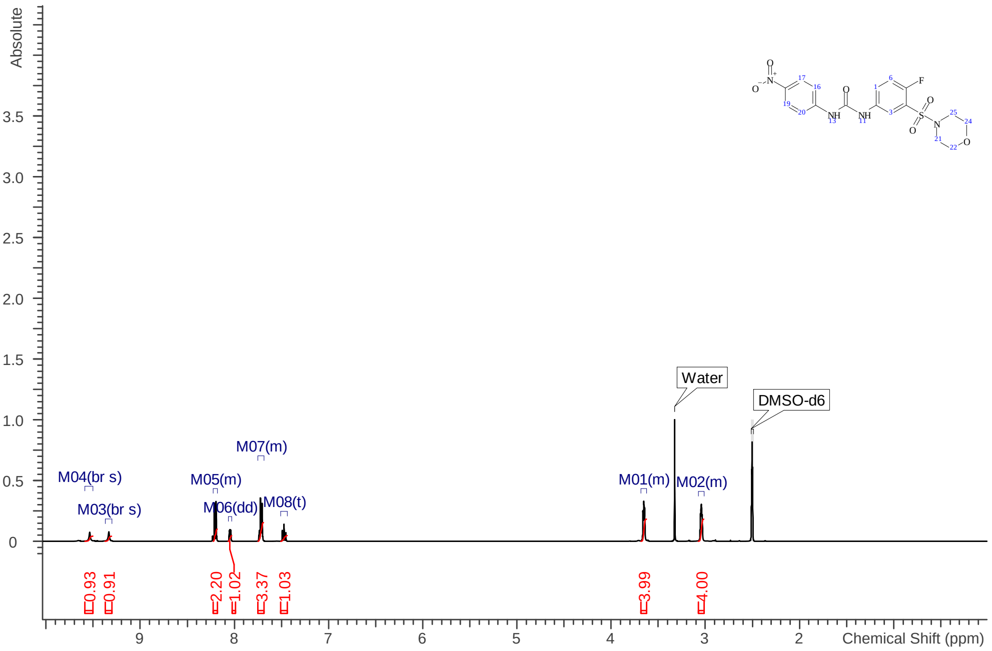
Absolute
Water
DMSO-d6
M04(br s)
M03(br s)
M05(m)
M06(dd)
M07(m)
M08(t)
M01(m)
M02(m)
0.93
0.91
2.20
1.02
3.37
1.03
3.99
4.00
Chemical Shift (ppm)

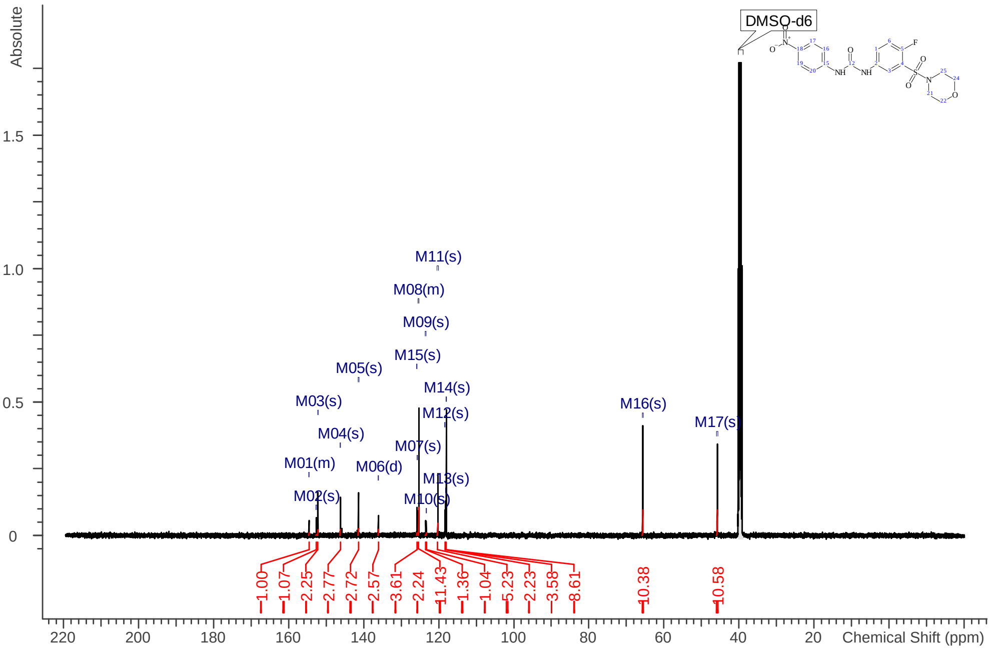
Absolute
DMSO-d6
M11(s)
M08(m)
M09(s)
M15(s)
M05(s)
M14(s)
M03(s)
M12(s)
M04(s)
M07(s)
M01(m)
M06(d)
M13(s)
M02(s)
M10(s)
M16(s)
M17(s)
1.00
1.07
2.25
2.77
2.72
2.57
3.61
2.24
11.43
1.36
1.04
5.23
2.23
3.58
8.61
10.38
10.58
Chemical Shift (ppm)

32

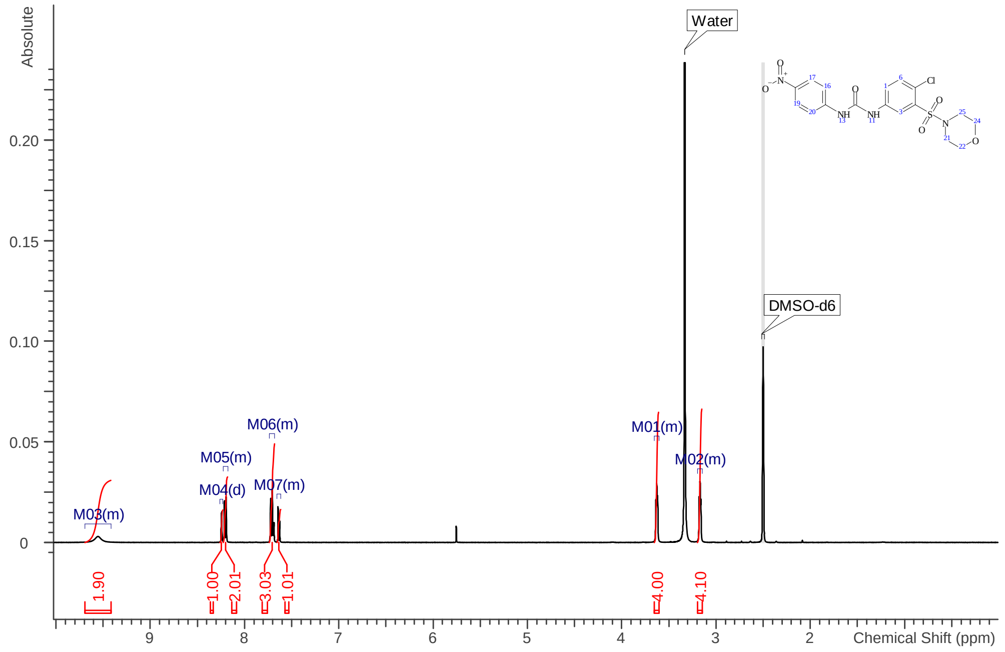
Absolute
Water
DMSO-d6
M06(m)
M05(m)
M04(d)
M07(m)
M03(m)
M01(m)
M02(m)
1.90
1.00
2.01
3.03
1.01
4.00
4.10
Chemical Shift (ppm)

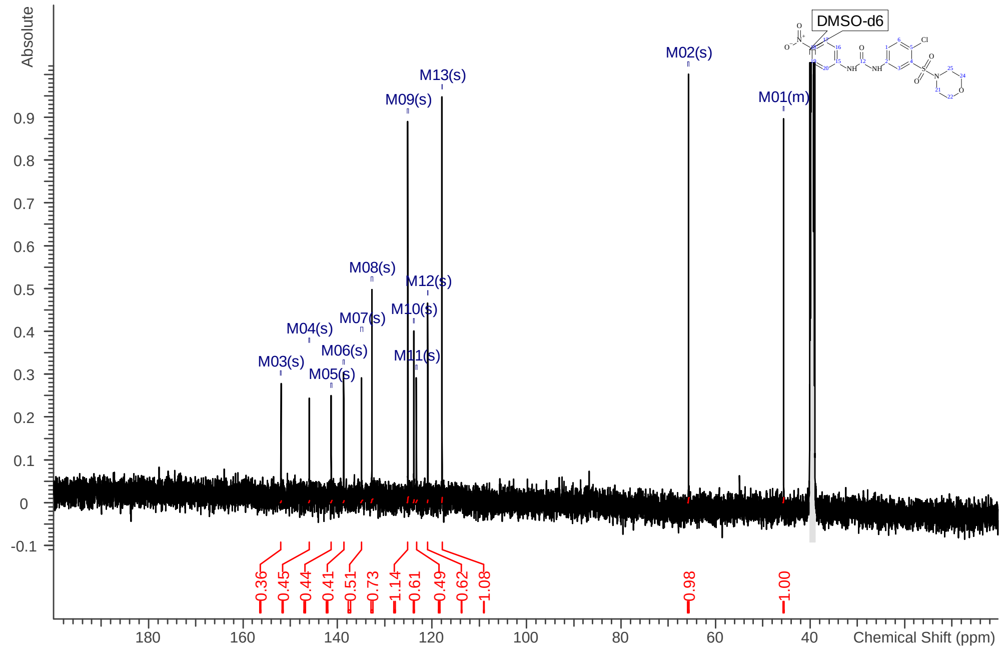
Absolute
DMSO-d6
M02(s)
M13(s)
M09(s)
M01(m)
M08(s)
M12(s)
M10(s)
M04(s)
M07(s)
M06(s)
M03(s)
M05(s)
M11(s)
0.36
0.45
0.44
0.41
0.51
0.73
1.14
0.61
0.49
0.62
1.08
0.98
1.00
Chemical Shift (ppm)

33

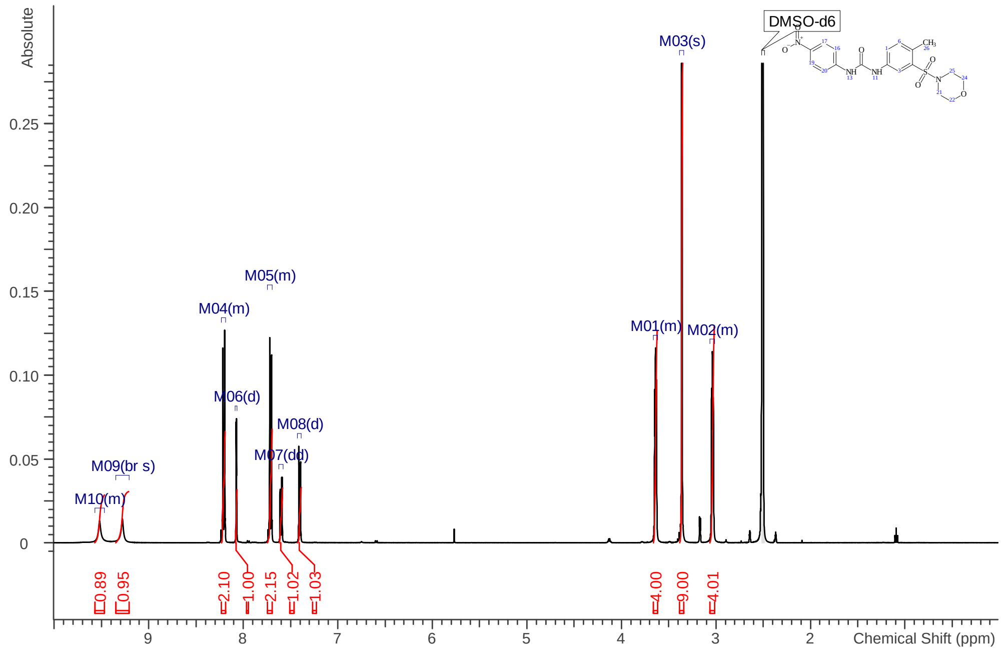
Absolute
DMSO-d6
M03(s)
M05(m)
M04(m)
M01(m)
M02(m)
M06(d)
M08(d)
M07(dd)
M09(br s)
M10(m)
0.89
0.95
2.10
1.00
2.15
1.02
1.03
4.00
9.00
4.01
Chemical Shift (ppm)

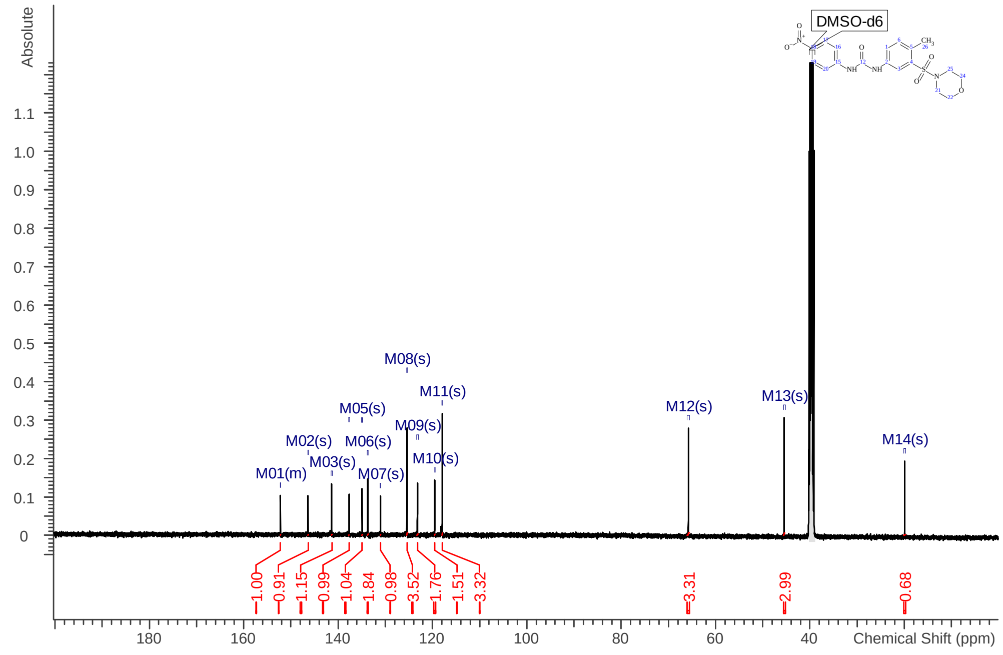
Absolute
DMSO-d6
M08(s)
M11(s)
M05(s)
M12(s)
M13(s)
M09(s)
M02(s)
M06(s)
M14(s)
M03(s)
M10(s)
M01(m)
M07(s)
1.00
0.91
1.15
0.99
1.04
1.84
0.98
3.52
1.76
1.51
3.32
3.31
2.99
0.68
Chemical Shift (ppm)

34

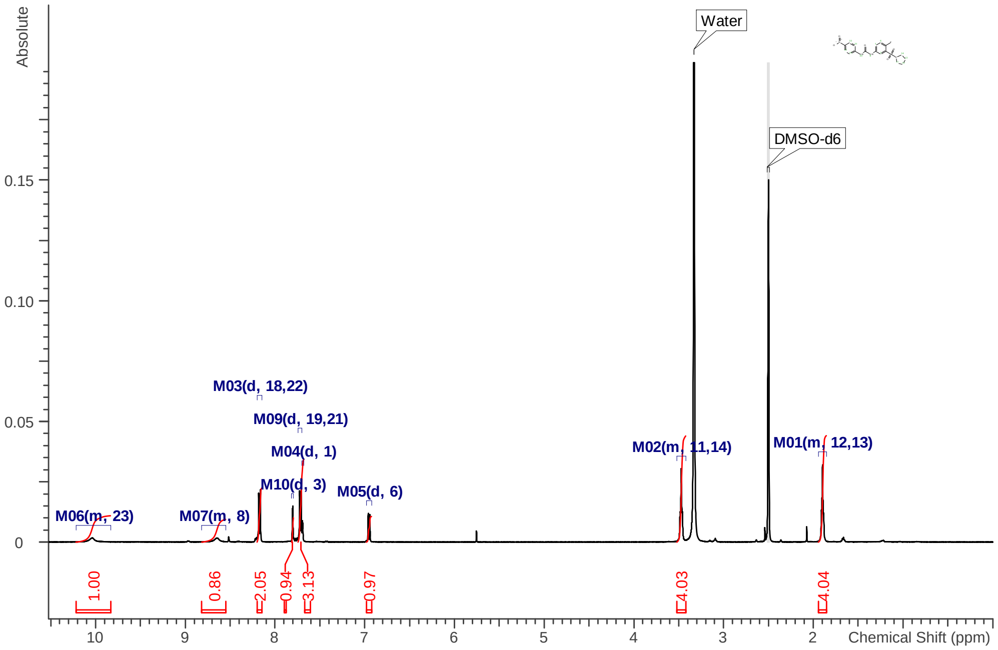

Absolute
Water
DMSO-d6
M03(d, 18,22)
M09(d, 19,21)
M04(d, 1)
M10(d, 3)
M05(d, 6)
M06(m, 23)
M07(m, 8)
M02(m, 11,14)
M01(m, 12,13)
1.00
0.86
2.05
0.94
3.13
0.97
4.03
4.04
Chemical Shift (ppm)

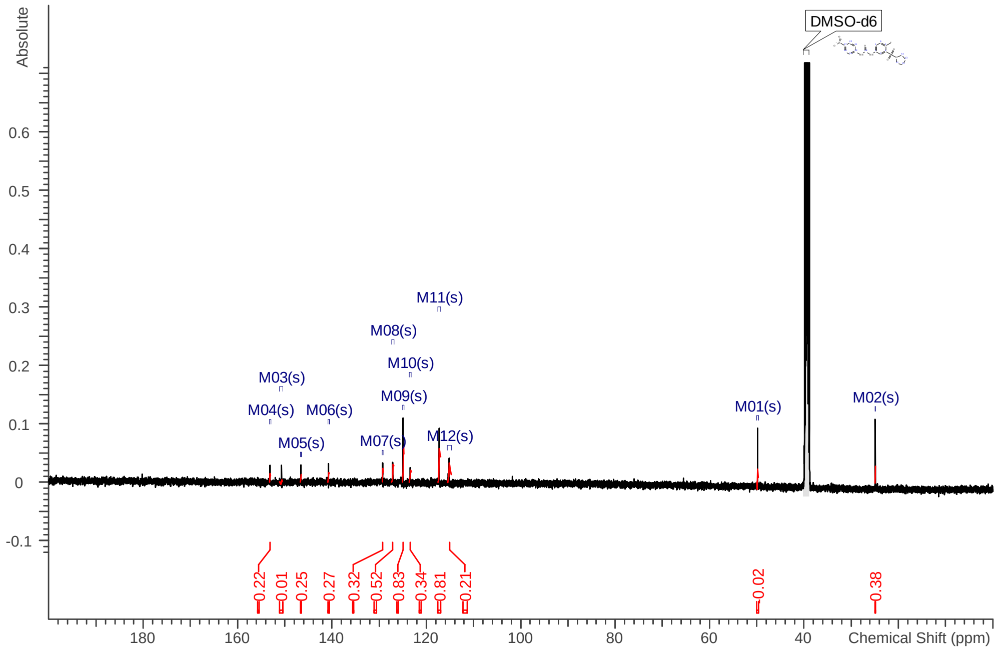

Absolute
DMSO-d6
M11(s)
M08(s)
M10(s)
M03(s)
M09(s)
M04(s)
M06(s)
M01(s)
M02(s)
M05(s)
M07(s)
M12(s)
0.22
0.01
0.25
0.27
0.32
0.52
0.83
0.34
0.81
0.21
0.02
0.38
Chemical Shift (ppm)

35

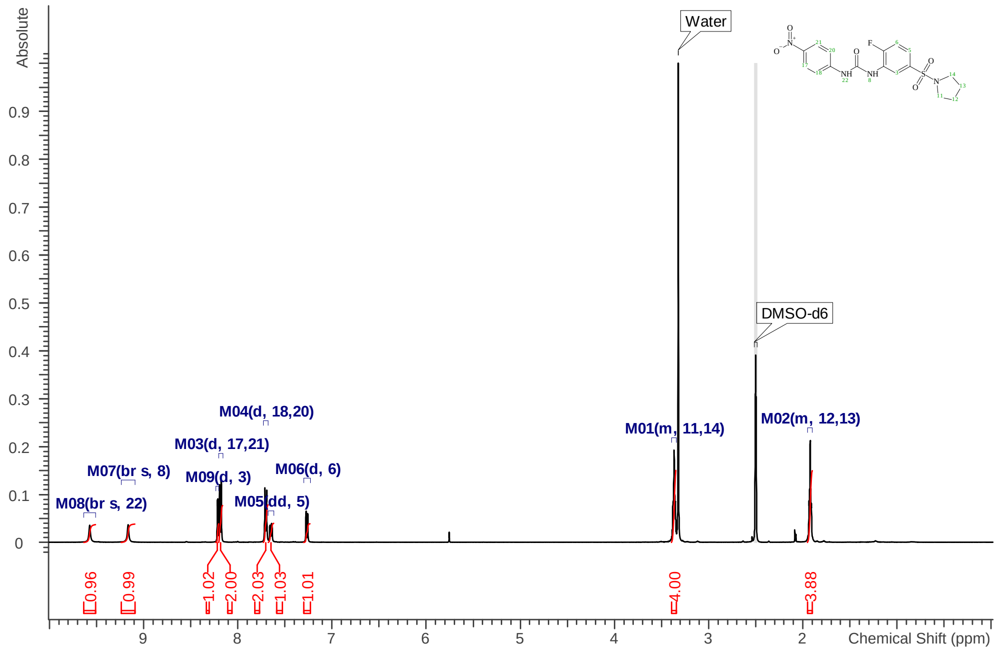

Water
DMSO-d6
M08(br s, 22)
M07(br s, 8)
M03(d, 17,21)
M04(d, 18,20)
M09(d, 3)
M05(dd, 5)
M06(d, 6)
M01(m, 11,14)
M02(m, 12,13)
0.96
0.99
1.02
2.00
2.03
1.03
1.01
4.00
3.88
Absolute
Chemical Shift (ppm)

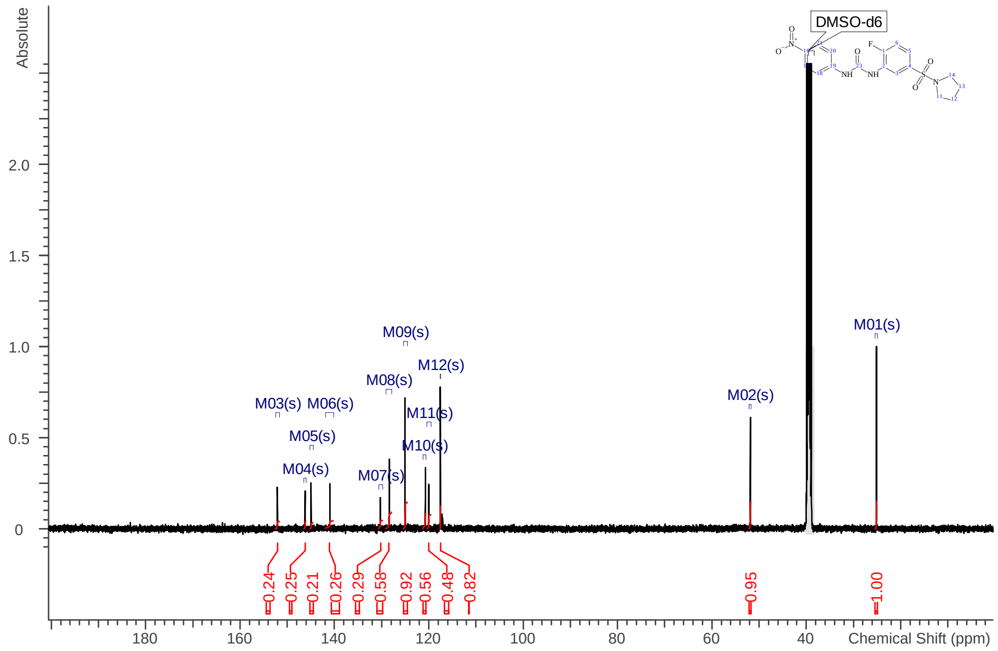

DMSO-d6
M03(s)
M06(s)
M05(s)
M04(s)
M07(s)
M08(s)
M09(s)
M12(s)
M11(s)
M10(s)
M02(s)
M01(s)
0.24
0.25
0.21
0.26
0.29
0.58
0.92
0.56
0.48
0.82
0.95
1.00
Absolute
Chemical Shift (ppm)

36

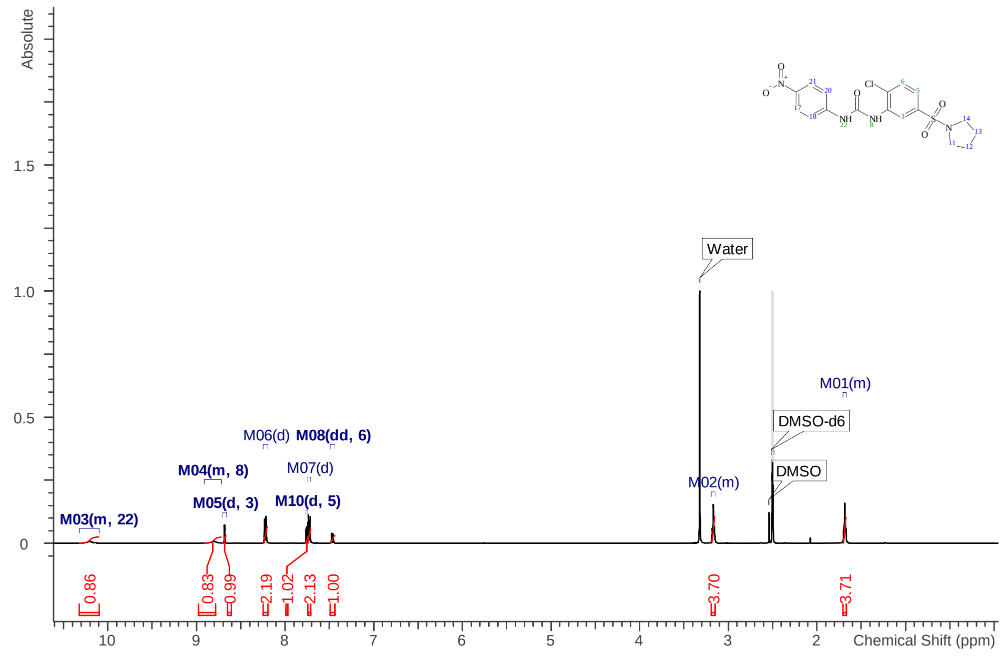

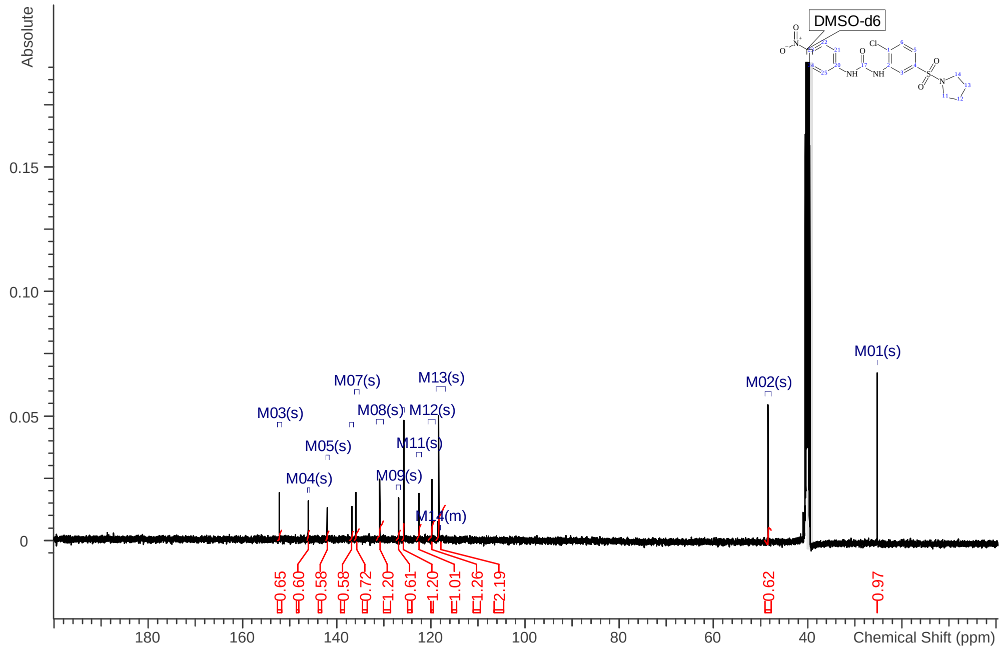

37

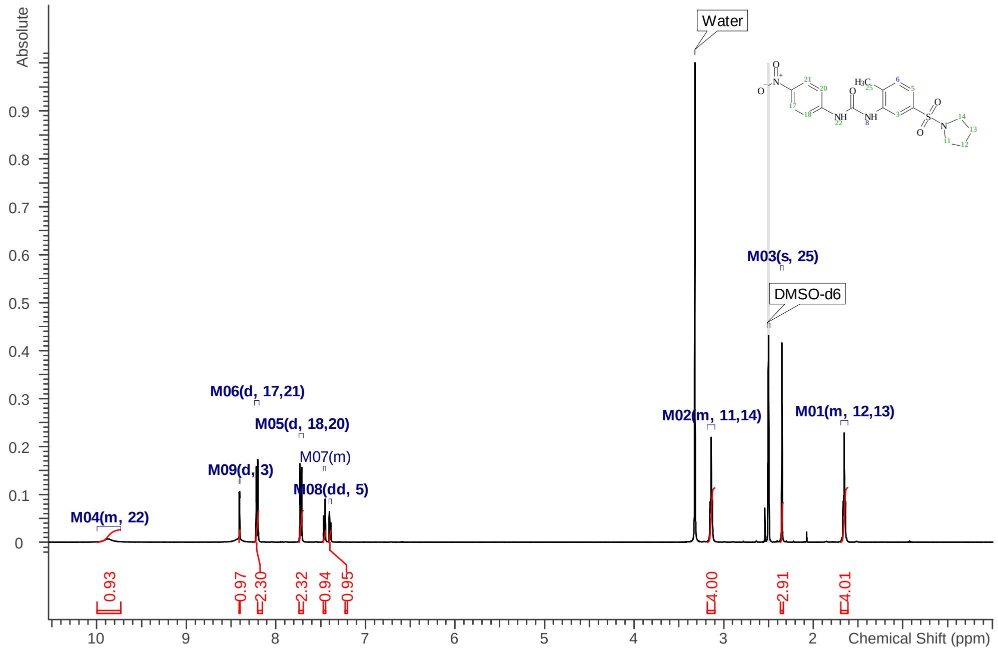

Absolute
Water
DMSO-d6
M03(s, 25)
M06(d, 17,21)
M05(d, 18,20)
M07(m)
M09(d, 3)
M08(dd, 5)
M04(m, 22)
M02(m, 11,14)
M01(m, 12,13)
0.93
0.97
2.30
2.32
0.94
0.95
4.00
2.91
4.01
Chemical Shift (ppm)

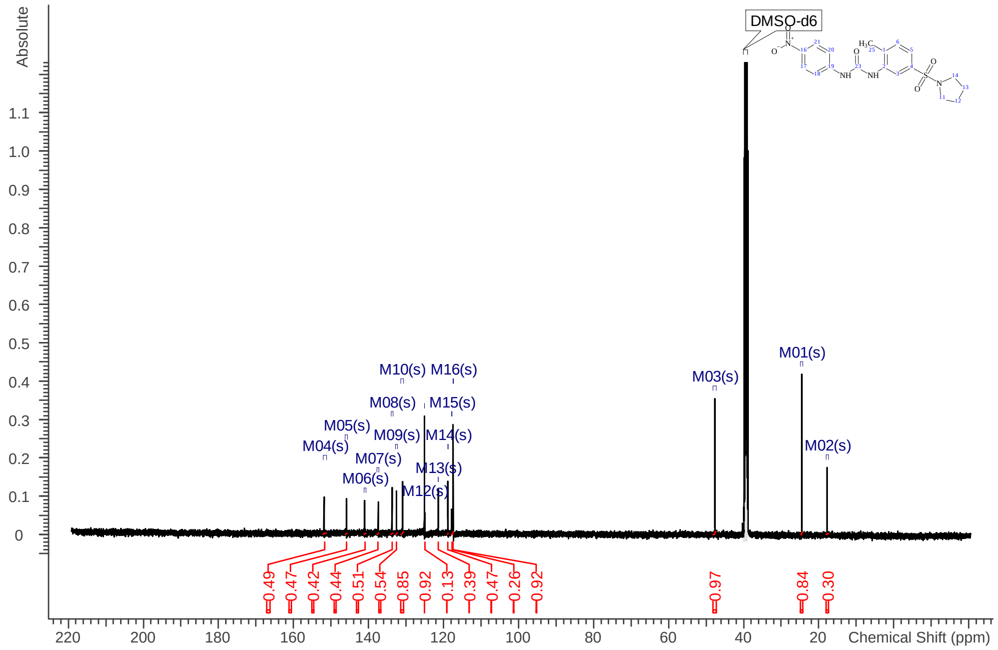

Absolute
DMSO-d6
M10(s) M16(s)
M08(s)
M15(s)
M05(s)
M09(s)
M14(s)
M04(s)
M07(s)
M13(s)
M06(s)
M12(s)
M03(s)
M01(s)
M02(s)
0.49
0.47
0.42
0.44
0.51
0.54
0.85
0.92
0.13
0.39
0.47
0.26
0.92
0.97
0.84
0.30
Chemical Shift (ppm)

38

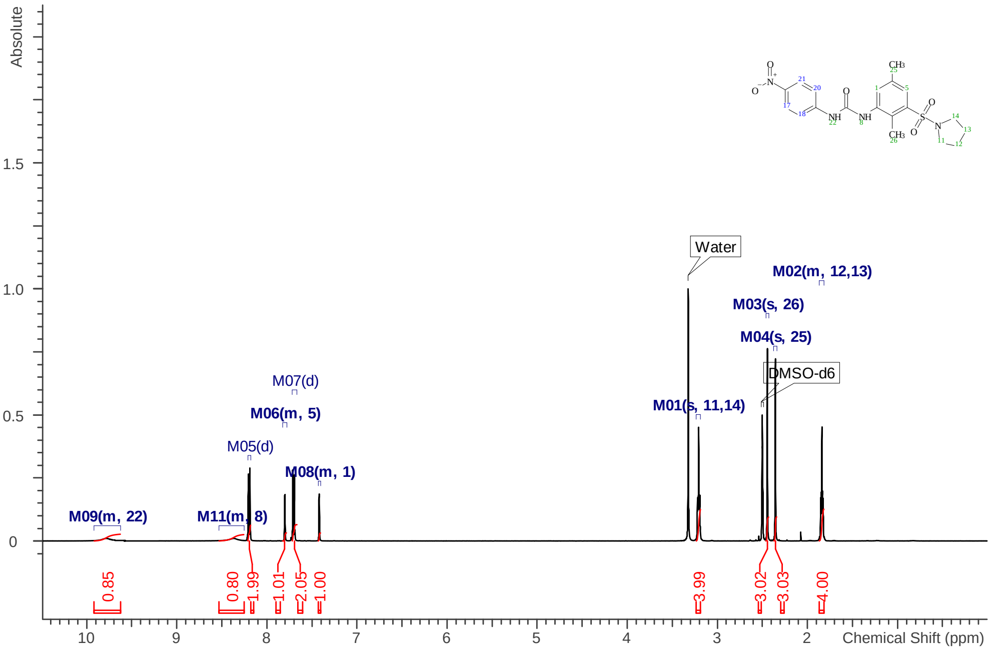

Absolute
Water
DMSO-d6
M02(m, 12,13)
M03(s, 26)
M04(s, 25)
M01(s, 11,14)
M07(d)
M06(m, 5)
M05(d)
M08(m, 1)
M09(m, 22)
M11(m, 8)
0.85
0.80
1.99
1.01
2.05
1.00
3.99
3.02
3.03
4.00
Chemical Shift (ppm)

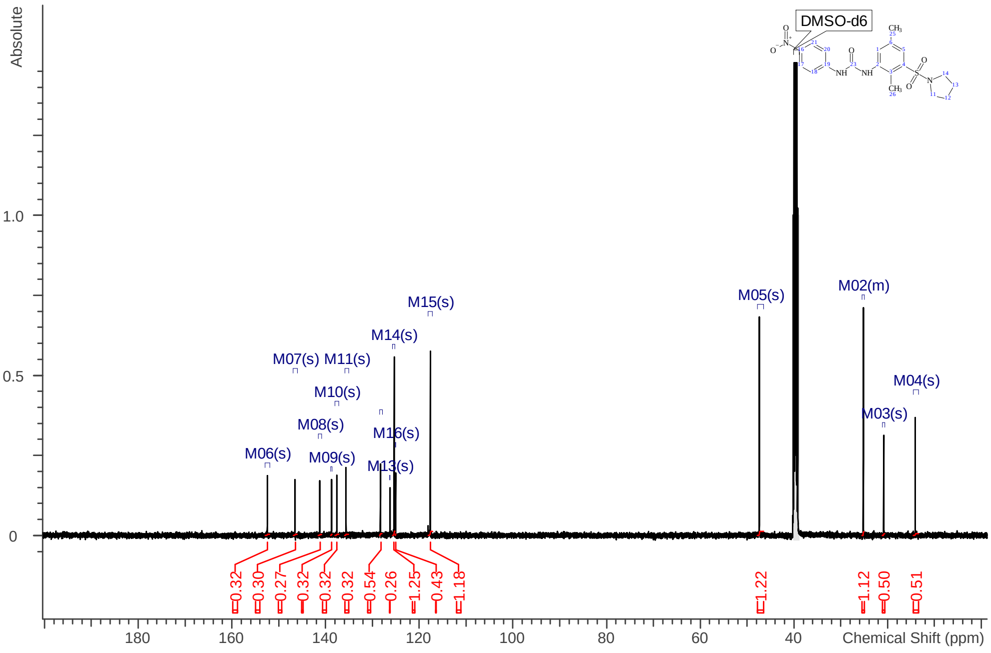

Absolute
DMSO-d6
M15(s)
M14(s)
M07(s) M11(s)
M10(s)
M08(s)
M16(s)
M06(s)
M09(s)
M13(s)
M05(s)
M02(m)
M04(s)
M03(s)
0.32
0.30
0.27
0.32
0.32
0.32
0.54
0.26
1.25
0.43
1.18
1.22
1.12
0.50
0.51
Chemical Shift (ppm)

39

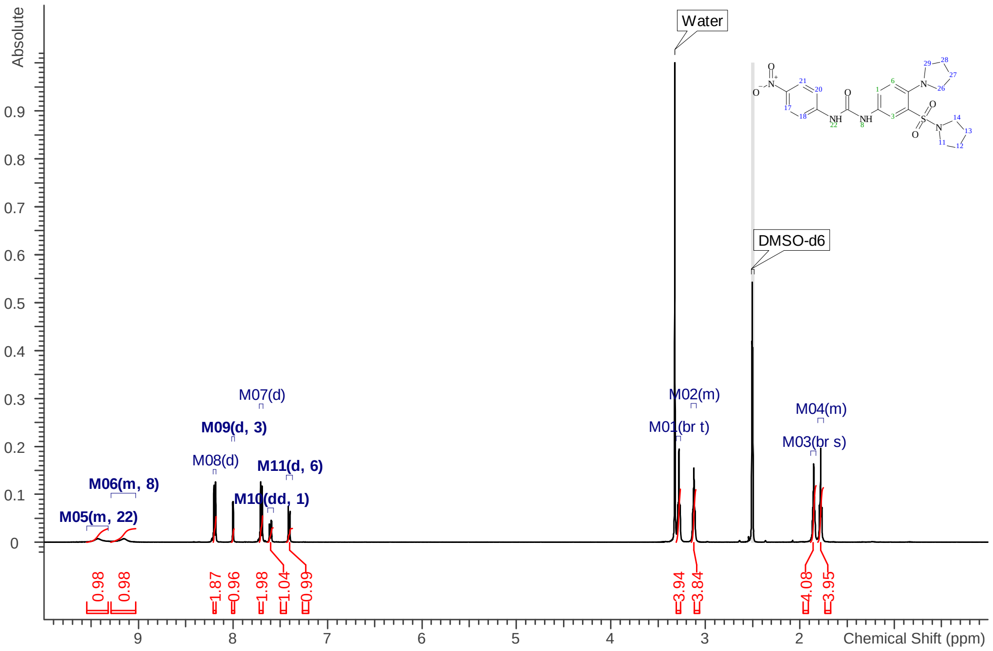

Absolute
Water
DMSO-d6
M07(d)
M09(d, 3)
M08(d)
M11(d, 6)
M06(m, 8)
M10(dd, 1)
M05(m, 22)
M02(m)
M01(br t)
M04(m)
M03(br s)
0.98
0.98
1.87
0.96
1.98
1.04
0.99
3.94
3.84
4.08
3.95
Chemical Shift (ppm)

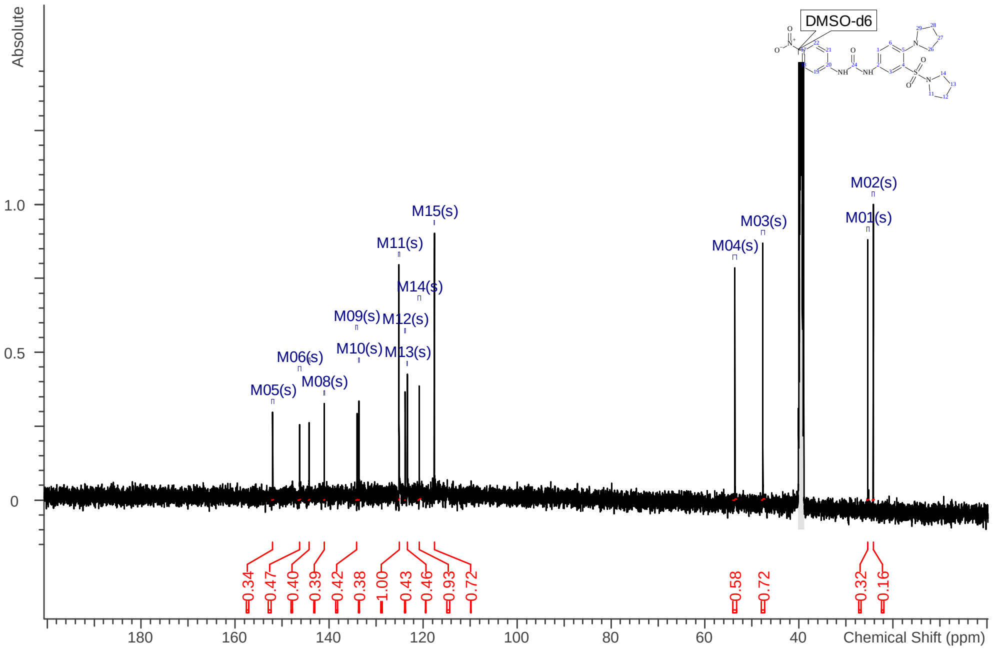

Absolute
DMSO-d6
M02(s)
M15(s)
M03(s)
M01(s)
M11(s)
M04(s)
M14(s)
M09(s)
M12(s)
M06(s)
M10(s)
M13(s)
M08(s)
M05(s)
0.34
0.47
0.40
0.39
0.42
0.38
1.00
0.43
0.46
0.93
0.72
0.58
0.72
0.32
0.16
Chemical Shift (ppm)

40

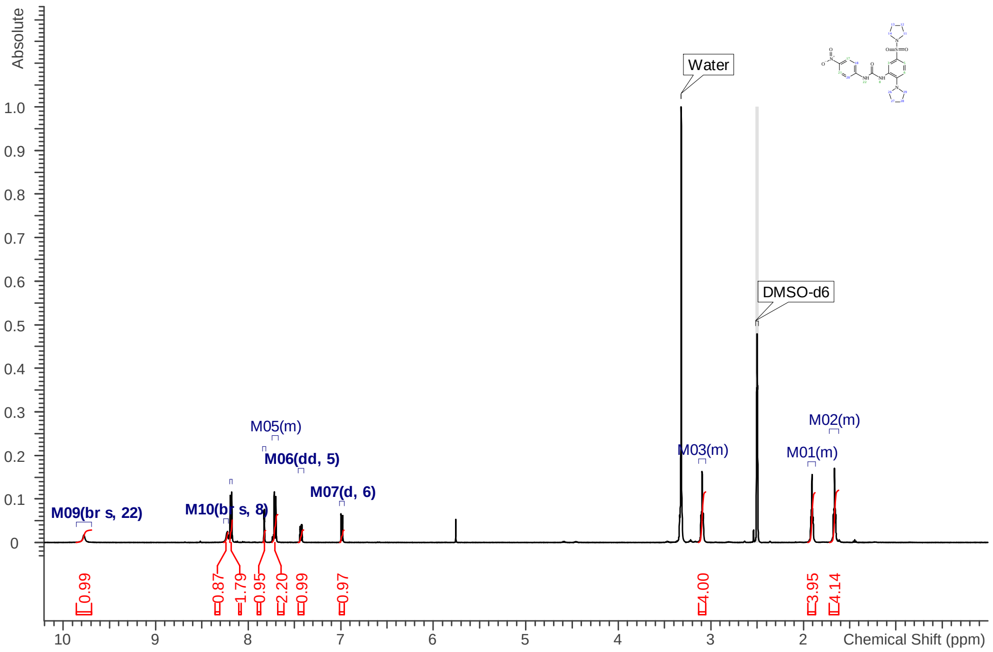

Absolute
Water
DMSO-d6
M05(m)
M06(dd, 5)
M07(d, 6)
M09(br s, 22)
M10(br s, 8)
M03(m)
M02(m)
M01(m)
0.99
0.87
1.79
0.95
2.20
0.99
0.97
4.00
3.95
4.14
Chemical Shift (ppm)

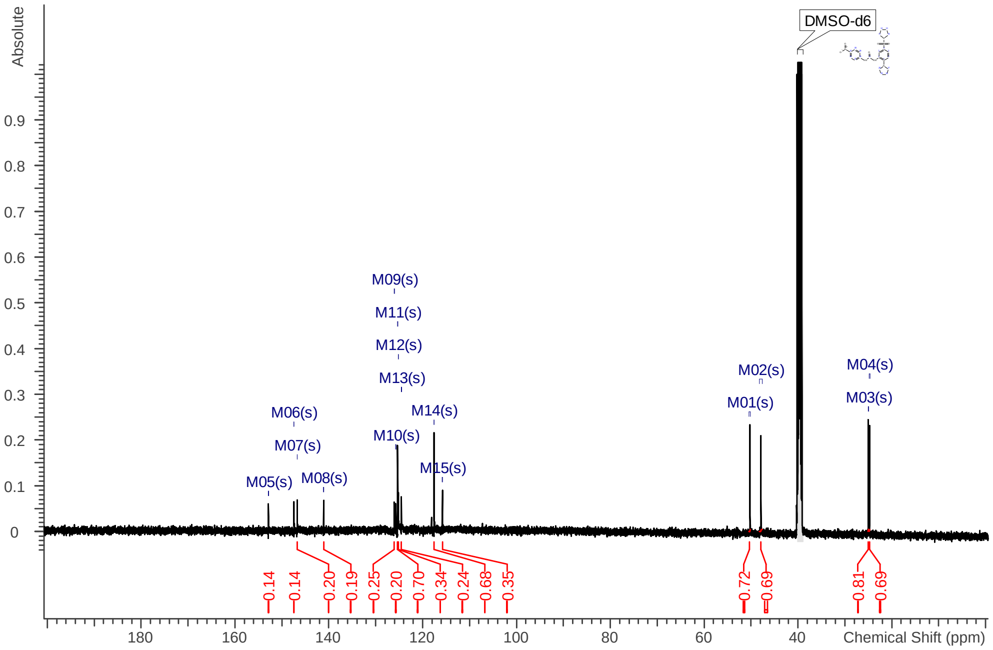

Absolute
DMSO-d6
M09(s)
M11(s)
M12(s)
M13(s)
M14(s)
M10(s)
M15(s)
M06(s)
M07(s)
M05(s)
M08(s)
M02(s)
M01(s)
M04(s)
M03(s)
Chemical Shift (ppm)

4

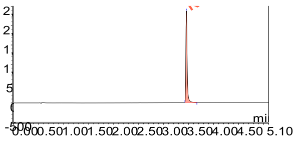

5

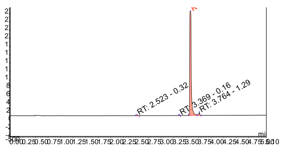

6

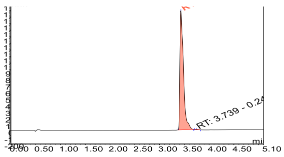

7

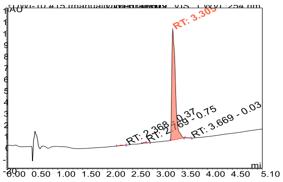

8

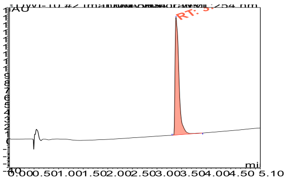

9

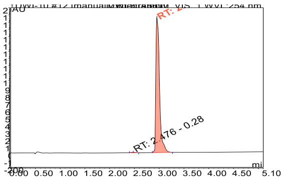

10

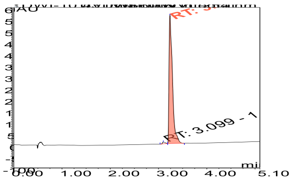
mAU
RT: 3.099 - 1
0.00
1.00
2.00
3.00
4.00
5.10
mi

11

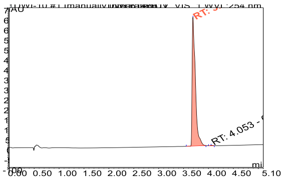
mAU
RT: 4.053 -
0.00
0.50
1.00
1.50
2.00
2.50
3.00
3.50
4.00
4.50
5.10
mi

12

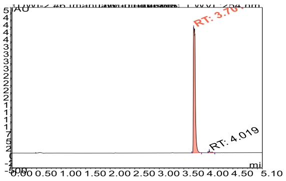
RT: 4.019
0.00 0.50 1.00 1.50 2.00 2.50 3.00 3.50 4.00 4.50 5.10

13

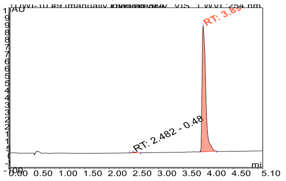
0.00 0.50 1.00 1.50 2.00 2.50 3.00 3.50 4.00 4.50 5.10

14

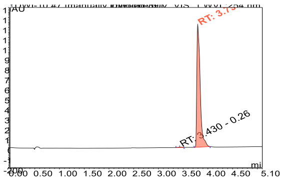
RT: 3.430 - 0.26
0.00 0.50 1.00 1.50 2.00 2.50 3.00 3.50 4.00 4.50 5.10

15

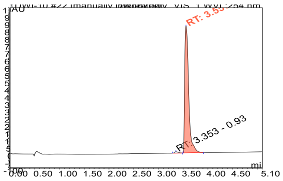
RT: 3.353 - 0.93
0.00 0.50 1.00 1.50 2.00 2.50 3.00 3.50 4.00 4.50 5.10

16

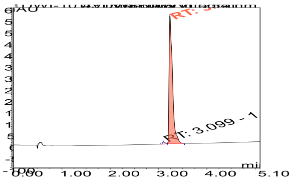

17

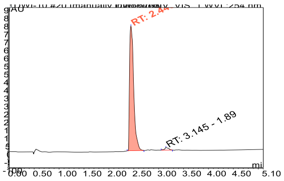

18

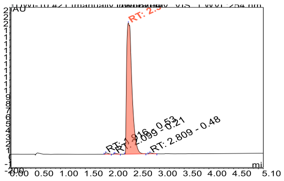

20

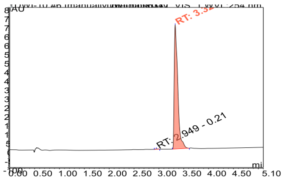

21

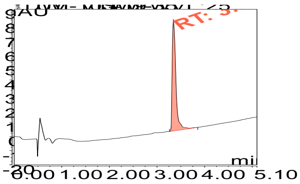

23

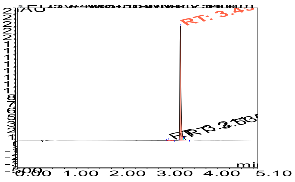

24

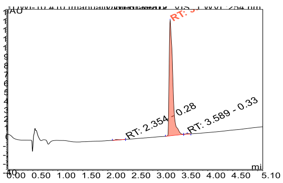
RT: 2.354 - 0.28
RT: 3.589 - 0.33
0.00 0.50 1.00 1.50 2.00 2.50 3.00 3.50 4.00 4.50 5.10

25

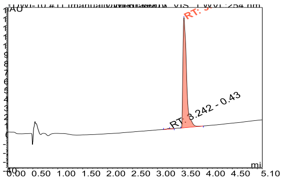
RT: 3.242 - 0.43
0.00 0.50 1.00 1.50 2.00 2.50 3.00 3.50 4.00 4.50 5.10

26

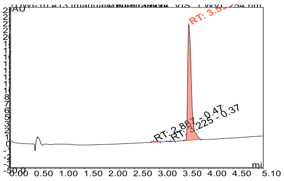
mAU
RT: 2.887 - 0.47
RT: 3.225 - 0.37
min
0.00 0.50 1.00 1.50 2.00 2.50 3.00 3.50 4.00 4.50 5.10

27

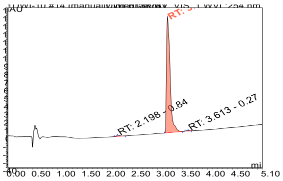
mAU
RT: 2.198 - 0.84
RT: 3.613 - 0.27
min
0.00 0.50 1.00 1.50 2.00 2.50 3.00 3.50 4.00 4.50 5.10

28

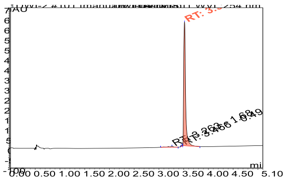

29

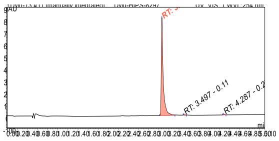

30

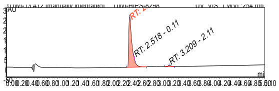

31

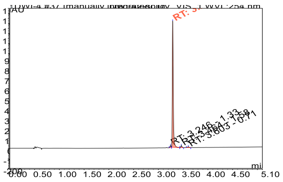

32

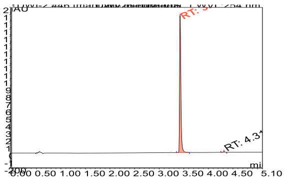

33

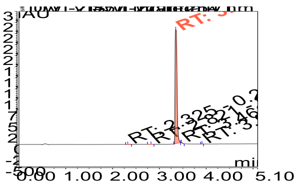

34

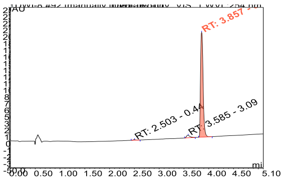

35

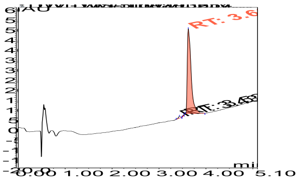

36

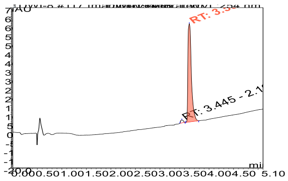

37

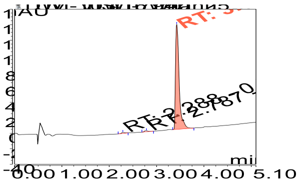

38

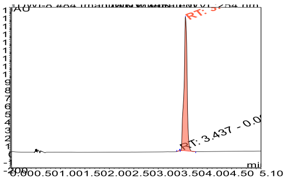

39

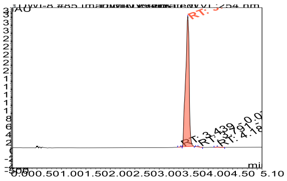

40

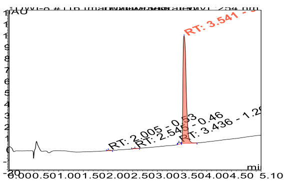

Supplement: Supplementary file 1 — jm4c00212_si_001.pdf [file jm4c00212_si_001.pdf]
